# Copper-catalyzed alkyne oxidation/Büchner-type ring-expansion to access benzo[6,7]azepino[2,3-*b*]quinolines and pyridine-based diones

**DOI:** 10.1038/s42004-023-00840-6

**Published:** 2023-02-20

**Authors:** Xiao-Lei Jiang, Qing Liu, Kua-Fei Wei, Ting-Ting Zhang, Guang Ma, Xiu-Hong Zhu, Guang-Xin Ru, Lijie Liu, Lian-Rui Hu, Wen-Bo Shen

**Affiliations:** 1 College of Sciences and College of Forestry, Henan Agricultural University, Zhengzhou, 450002 China; 2 Sanmenxia Polytechnic, Sanmenxia, Henan 472000 China; 3 Shanghai Key Laboratory of Green Chemistry and Chemical Processes, Shanghai Frontiers Science Center of Molecule Intelligent Syntheses, School of Chemistry and Molecular Engineering, East China Normal University, Shanghai, 200062 China

**Keywords:** Synthetic chemistry methodology, Reaction mechanisms, Organocatalysis, Homogeneous catalysis

## Abstract

General access to highly valuable seven-membered rings via Büchner-type reaction remains a formidable challenge. Here we report a Cu-catalyzed intermolecular oxidation of alkynes using N-oxides as oxidants, which enables expedient preparation of valuable benzo[6,7]azepino[2,3-b]quinolines and pyridine-based diones. Importantly, in contrast to the well-established gold-catalyzed intermolecular alkyne oxidation, the dissociated pyridine or quinoline partner could be further utilized to construct N-heterocycles in this system and the reaction most likely proceeds by a Büchner-type ring expansion pathway. A mechanistic rationale for this cascade cyclization is supported by DFT calculations.

## Abstract (web summary)

Organic molecules containing seven-membered rings are important structural motifs for drug candidates, but the construction of seven-membered rings can be challenging through traditional cyclization pathways. Here, the authors report a copper-catalyzed alkyne oxidation and Büchner-type ring-expansion to generate benzo[6,7]azepino[2,3-b]quinolines and pyridine-based diones.

## Introduction

Medium-sized ring-containing organic molecules, especially the seven-membered rings, are important structural motifs that are found in drug candidates as well as in bioactive molecules^1–7^. However, due to entropic effects and transannular interactions, such frameworks are regarded as difficult structures to access^8,9^. Compared to the synthesis of five- and six-membered rings, the construction of seven-membered rings can be more challenging through traditional cyclization pathways. Thus, the development of an efficient method to build these seven-membered rings has attracted a significant amount of research attention. Among the numerous methods developed so far, the Büchner-type ring-expansion reaction which has become an effective method for the preparation of seven-membered rings has attracted much attention during the last decade^10–14^. Traditionally, the Büchner reaction is triggered by the cyclopropanation of the benzene ring to give a norcaradiene, and then the electrocyclic ring opening provides a cycloheptatriene. However, this strategy is hindered by the nature of these α-diazo ketone precursors, which are hazardous, not easily accessible, and potentially explosive. Consequently, the development of catalytic approaches is highly desirable, especially from readily and generally available precursors.

Recently, gold-catalyzed intermolecular alkyne oxidation presumably involving α-oxo gold carbenes has burgeoned, as it avoids the use of difficult and hazardous diazo compounds^15–26^.

**Fig. 1:**
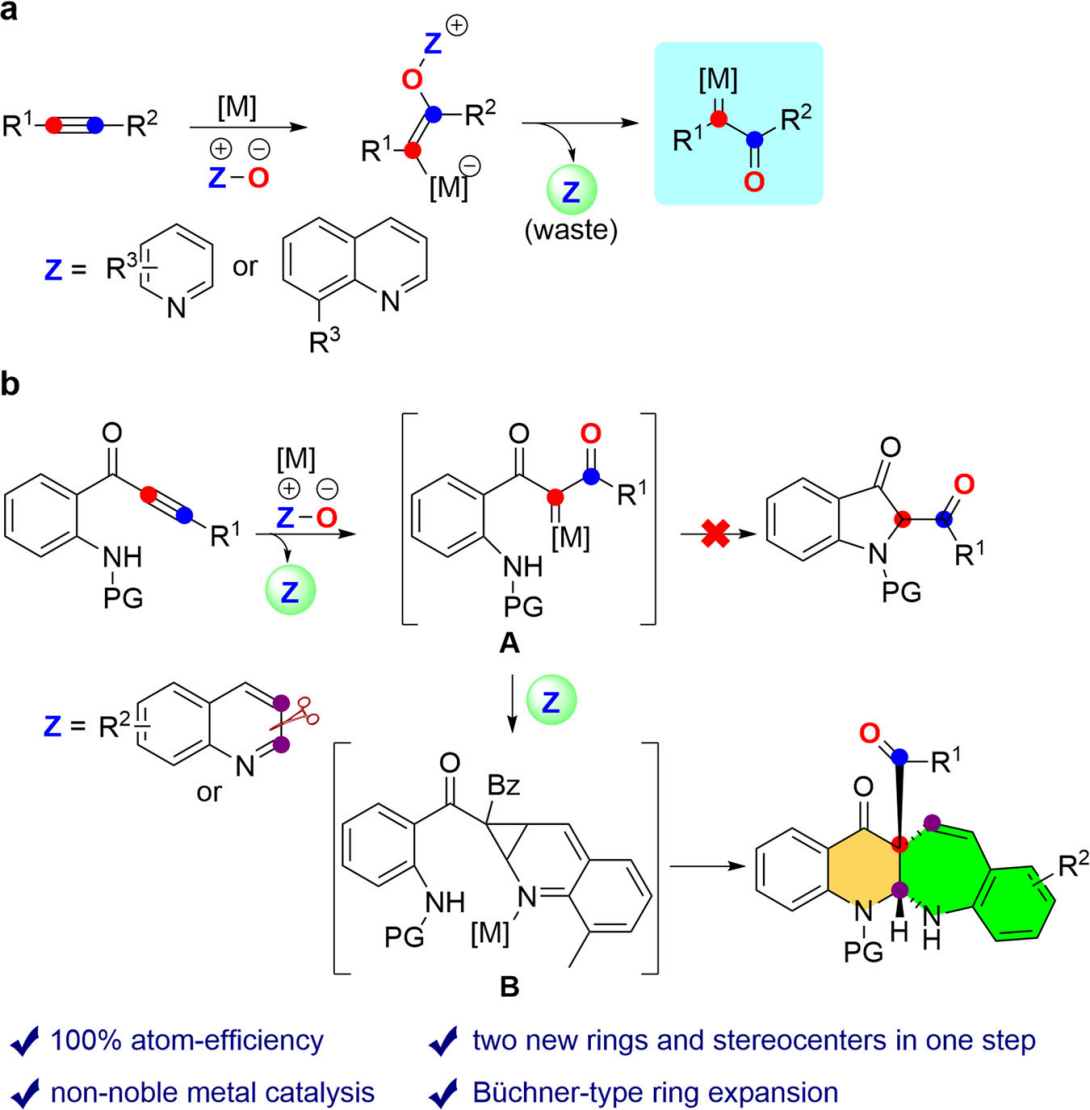
Typical ways for the generation of α-oxo metal carbenes. **a** Previous work. **b** Our initial design. M metal.

In 2010, Zhang et al.^27^ disclosed an elegant protocol for the gold-catalyzed intermolecular oxidation of alkynes via a reactive α-oxo gold carbene intermediate (Fig. 1). In addition, the Tang group described that rhodium could also catalyze such an intermolecular alkyne oxidation^28^. Following this notion, numerous efficient synthetic methods have also been disclosed by Hashmi^29,30^, Liu^31–34^, Ye^35,36^ and others based on this strategy, affording functionalized heterocycles^37–49^. Despite these great successes, this intermolecular pathway is evidently not atom-economical due to the fact that the procedure generates a stoichiometric amount of pyridines or quinolines, as waste, which may coordinate and poison metal catalysts. Furthermore, a noble-metal catalyst usually is required, and may severely hamper the practical application of this strategy owing to the high cost and toxicity of the catalyst. In our ongoing program of expanding copper catalysis into alkyne transformation^50–56^, we develop a copper-catalyzed alkyne oxidation/Büchner-type ring-expansion sequence, leading to the benzo[6,7]azepino[2,3-b]quinolines and pyridine-based diones. In particular, the pyridine or quinoline partner could be further utilized to construct N-heterocycles in such an oxidative copper catalysis. Most importantly, mechanistic studies and theoretical calculations demonstrate that the reaction presumably proceeds by a Büchner-type pathway, which is distinctively different from the related gold-catalyzed oxidative cyclization. General access to highly valuable seven-membered rings via Büchner-type reaction remains a formidable challenge. Herein, we describe a copper-catalyzed alkyne oxidation/Büchner-type ring-expansion sequence, thus providing practical access to synthetically useful fused seven-membered ring cyclic compounds. Cyclopropanations of heteroarenes are shown in an intermolecular Büchner-type reaction, while circumventing the use of hazardous diazo carbonyl substrates.

## Results and discussion

### Optimization of the reaction conditions

At the outset, alkynone **1a** and 8-methylquinoline *N*-oxide **2a** were chosen as model substrates and a range of experiments were executed in order to authenticate our opinion. As documented in Table 1, our initial examination focused on the reaction of the alkynone **1a** with 8-methylquinoline *N*-oxide **2a** in DCE at 80 °C in the presence of a copper catalyst. To our pleasure, the expected benzo[6,7]azepino[2,3-*b*]quinoline **3a** was certainly formed in 23% yield, albeit with a lower yield (Table 1, entry 1). The molecular structure of **3a** was further confirmed by X-ray diffraction^57^. Subsequent other copper catalyst screenings indicated that the Cu(hfacac)_2_ performed obviously better (entries 2–6). Other Lewis acids, including Zn(OTf)_2_, Y(OTf)_3_ and Sc(OTf)_3_, failed to further improve the reaction efficiency (entries 7–9). In addition, the desired **3a** was detected in 43% yield when the solvent was changed from DCE to toluene (entry 10). Raising the reaction temperature to 120 °C improved the product yield considerably to 58% (entries 11–12). Doubling of catalyst loading led to an even better yield, affording **3a** in 69% yield (entry 13). It should be mentioned that the reaction failed to give even a trace of **3a** in the absence of the catalyst (entry 14). Finally, the addition of 4 Å MS led to a slight increase in the yield, forming **3a** in 76% yield (entry 15).

**Table 1:** Optimization of reaction conditions^a^.


Entry	Cat. (*x* mol %)	Conditions	Yield (%)^b^
1	Cu(CH_3_CN)_4_PF_6_ (10)	DCE, 80 °C, 55 h	23
2	Cu(CH_3_CN)_4_BF_4_ (10)	DCE, 80 °C, 60 h	19
3	CuOTf (10)	DCE, 80 °C, 60 h	26
4	Cu(PPh_3_)_3_Br (10)	DCE, 80 °C, 72 h	<1
5	Cu(OTf)_2_ (10)	DCE, 80 °C, 62 h	25
6	Cu(hfacac)_2_ (10)	DCE, 80 °C, 48 h	37
7	Zn(OTf)_2_ (10)	DCE, 80 °C, 29 h	<1
8	Y(OTf)_3_ (10)	DCE, 80 °C, 75 h	27
9	Sc(OTf)_3_ (10)	DCE, 80 °C, 80 h	18
10	Cu(hfacac)_2_ (10)	Toluene, 80 °C, 46 h	43
11	Cu(hfacac)_2_ (10)	Toluene, 100 °C, 20 h	51
12	Cu(hfacac)_2_ (10)	Toluene, 120 °C, 9 h	58
13	Cu(hfacac)_2_ (20)	Toluene, 120 °C, 8 h	69
14	None	Toluene, 120 °C, 8 h	<1
**15**^**c**^	**Cu(hfacac)**_**2**_ **(20)**	**Toluene, 120** **°C, 6** **h**	**76**

^a^Reaction conditions: **1a** (0.1 mmol), **2a** (0.2 mmol), catalyst (5–20 mol %), 0.05 M, 80–120 °C, in vials.

^b^Measured by ^1^H NMR using diethyl phthalate as the internal standard.

^c^Using 4 Å molecular sieves (20 mg/0.1 mmol) as an additive.

Bold text highlights the optimal reaction condition.

### Substrate scope with different alkynones

**Fig. 2:**
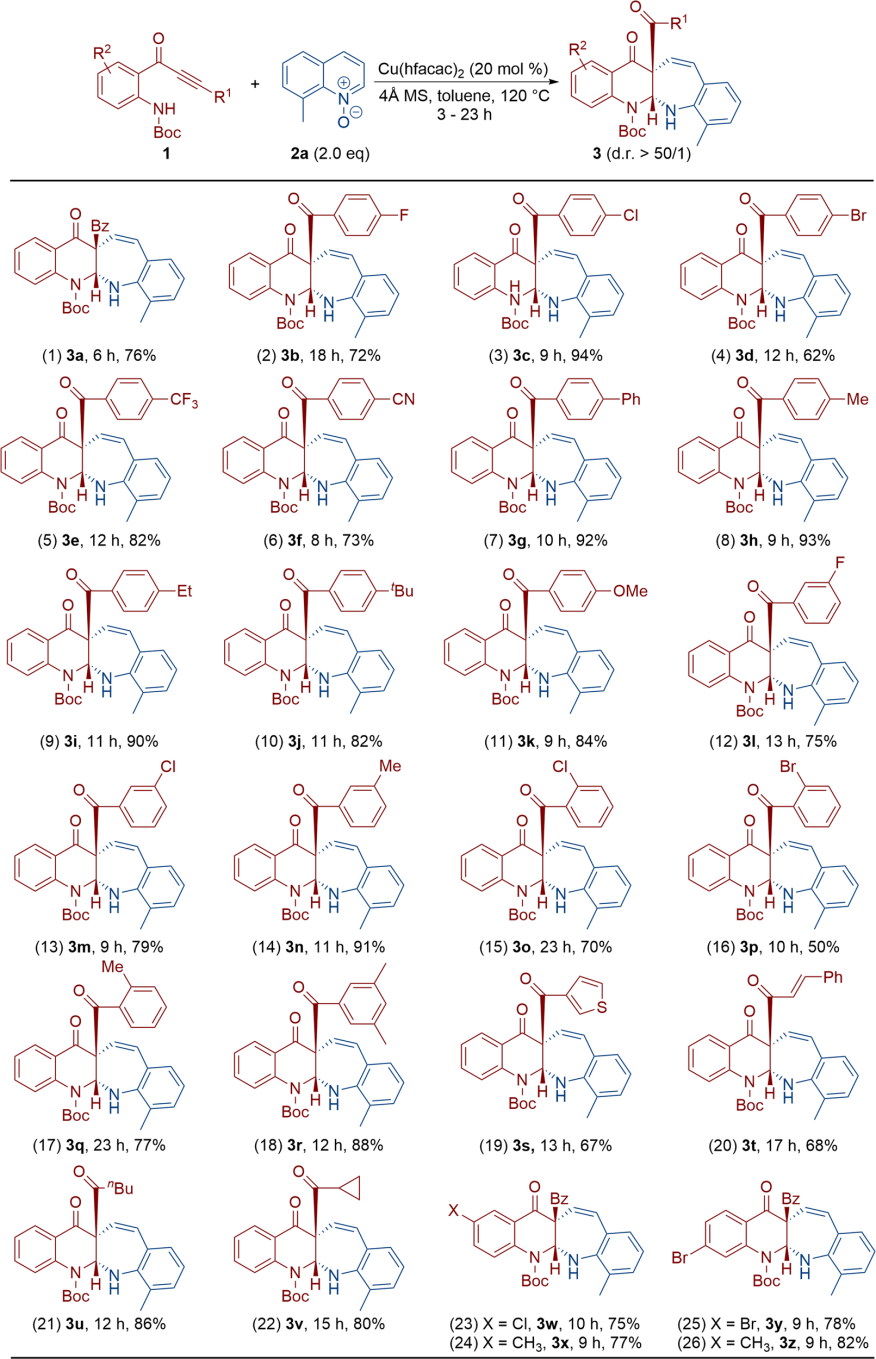
Reaction scope with different alkynones 1. Reaction conditions: [**1**] = 0.05 M; yields are those for the isolated products.

With the optimized reaction conditions in hand, we then set out to assess the scope of the reaction by varying alkynones **1**. The results are presented in Fig. 2. Alkynones with varied aryl groups (Ar = 4-XC_6_H_4_, X = F, Cl, Br, CF_3_, CN, Ph, Me, Et, ^*t*^Bu and OMe) at the C-4 position were first examined, delivering the corresponding benzo[6,7]azepino[2,3-*b*]quinolines **3a**–**k** in 62–94% yields (entries 1–11). In addition, aryl-substituted alkynones bearing both electron-withdrawing and electron-donating substituents, such as F, Cl and Me, on the phenyl ring were also compatible with this tandem reaction, thus leading to the resulting benzo[6,7]azepino[2,3-*b*]quinolines **3l**–**r** in 50–91% yields (entries 12–18). In particular, the reaction proceeded smoothly with alkynones bearing sterically hindered *ortho* substituents. The molecular structure of **3r** was further confirmed by X-ray diffraction^57^. To our satisfaction, thiophene, styryl, ^*n*^Bu and cyclopropyl-substituted alkynones were also suitable substrates for this transformation, affording the expected benzo[6,7]azepino[2,3-*b*]quinolines **3s**–**v** in 67–86% yields (entries 19–22). For alkynones bearing different R^2^ substituents, their desired products **3w**–**z** were obtained in 75–82% yields (entries 23–26).

### Substrate scope with different quinoline *N*-oxides

**Fig. 3:**
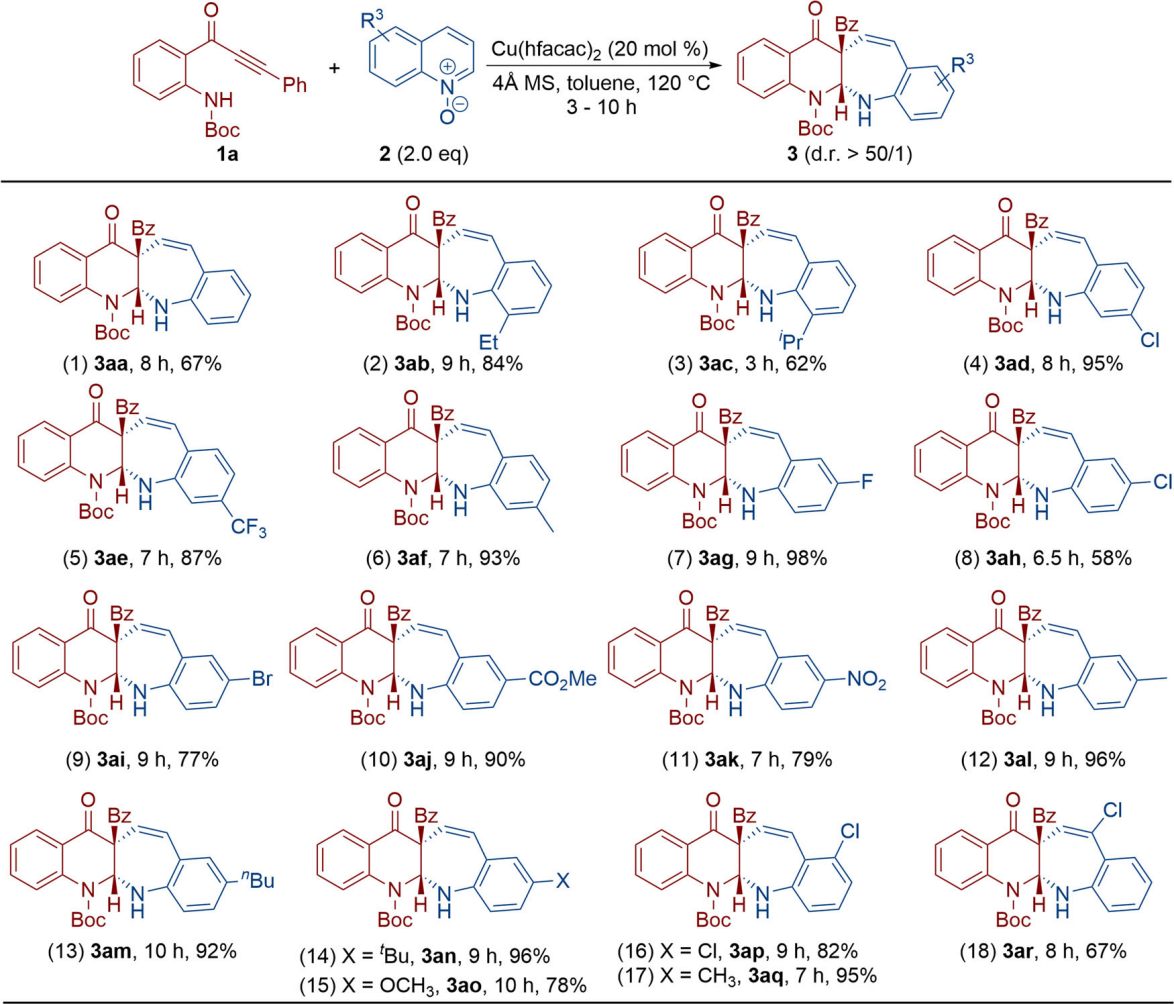
Reaction scope with different quinoline *N*-oxides 2. Reaction conditions: [**1**] = 0.05 M; yields are those for the isolated products.

We next extended the reaction to a variety of quinoline *N*-oxides **2** (Fig. 3). Unsubstituted quinoline *N*-oxide was first performed, giving the corresponding benzo[6,7]azepino[2,3-*b*]quinoline **3aa** in 67% yield (entry 1). The products **3ab** and **3ac** were also formed in 84 and 62% yields, respectively, when 8-methylquinoline *N*-oxide was replaced by 8-ethylquinoline *N*-oxide or 8-isopropylquinoline *N*-oxide (entries 2–3). Compounds **2e**–**g** (*N*-oxide = 7-chloroquinoline *N*-oxide, 7-trifluoromethylquinoline *N*-oxide and 7-methylquinoline *N*-oxide) were converted smoothly into benzo[6,7]azepino[2,3-*b*]quinolines **3ad**–**af** in 87–95% yields (entries 4–6). This tandem reaction also proceeded for 6-substituted quinoline *N*-oxides, including substrates with fluoro, chloro, bromo, methyl formate, nitro, methyl, *n*-butyl, *t*-butyl and methoxy substituents, and the resulting **3ag**–**ao** were obtained in 58–98% yields (entries 7–15). The related reactions of quinoline *N*-oxides with additional substitutions at the 5-position and 4-position were either equally or more efficient, affording the expected benzo[6,7]azepino[2,3-*b*]quinolines **3ap**–**ar** in 67–95% yields (entries 16–18). Accordingly, this approach provided a general and highly efficient strategy for the construction of polycyclic *N*-heterocycles in organic synthesis. Notable is that the reaction substrates were not only limited to 8-alkylquinoline *N*-oxides as the oxidants^58^.

### Reaction scope for the formation of pyridine-based diones

**Fig. 4:**
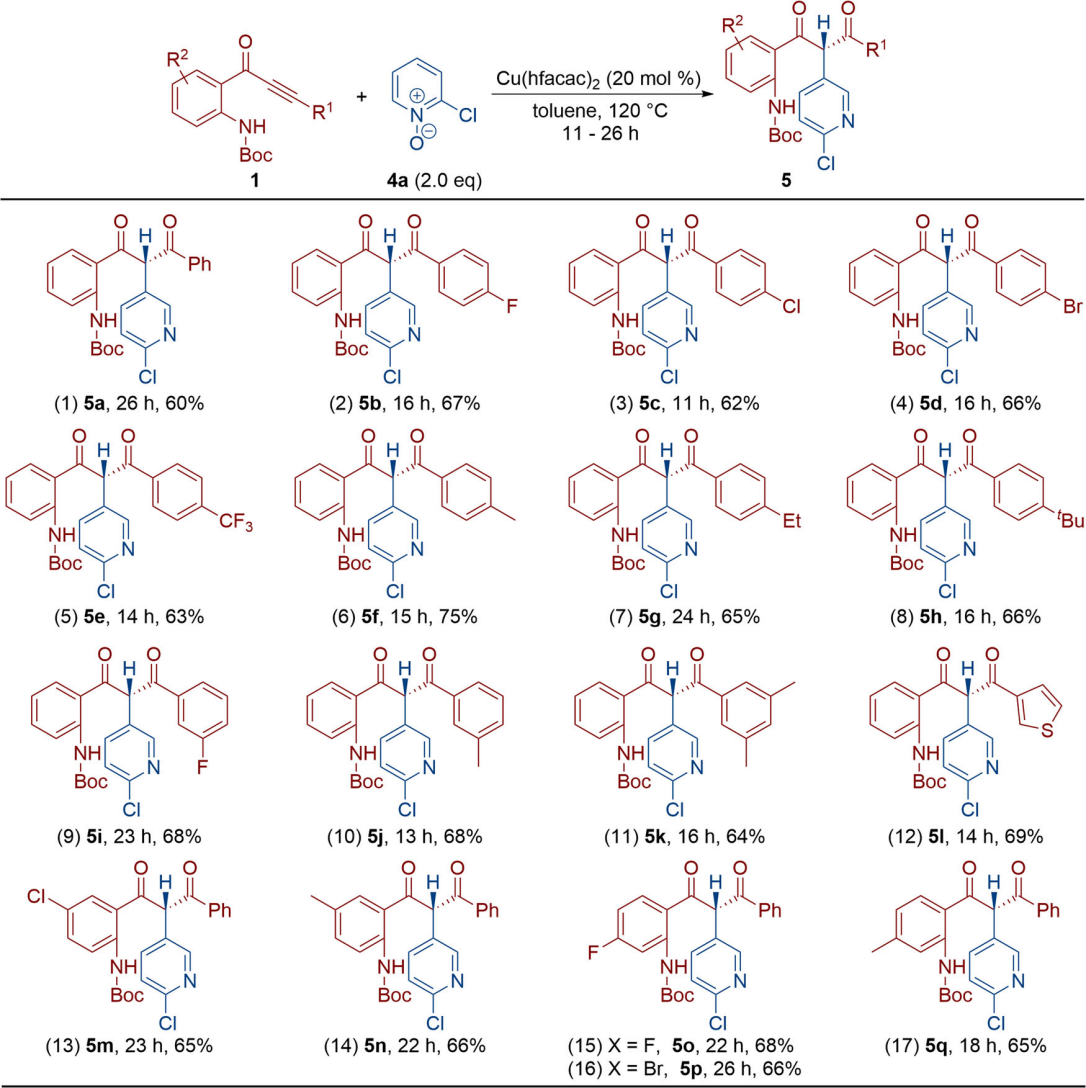
Reaction scope for the formation of pyridine-based diones 5. Reaction conditions: [**1**] = 0.05 M; yields are those for the isolated products.

Besides quinoline *N*-oxides, the reaction also proceeded well with pyridine *N*-oxides to furnish unexpected pyridine-based diones. Thus, the treatment of alkynones **1** with pyridine *N*-oxide **4a** under copper catalysis furnished the resulting pyridine-based diones **5a**–**q** in 60–75% yield (Fig. 4). The molecular structure of **5p** was further confirmed by X-ray diffraction^57^. The reaction presumably involved a copper-catalyzed oxidation-initiated tandem alkyne oxidation/Büchner-type/[1,2]-H shift, and the formation of pyridine-based diones instead of the previous benzo[6,7]azepino[2,3-*b*]quinolines was attributed to the relatively lower activity (mechanism for the formation of **5a** is depicted in Supplementary Information).

**Fig. 5:**
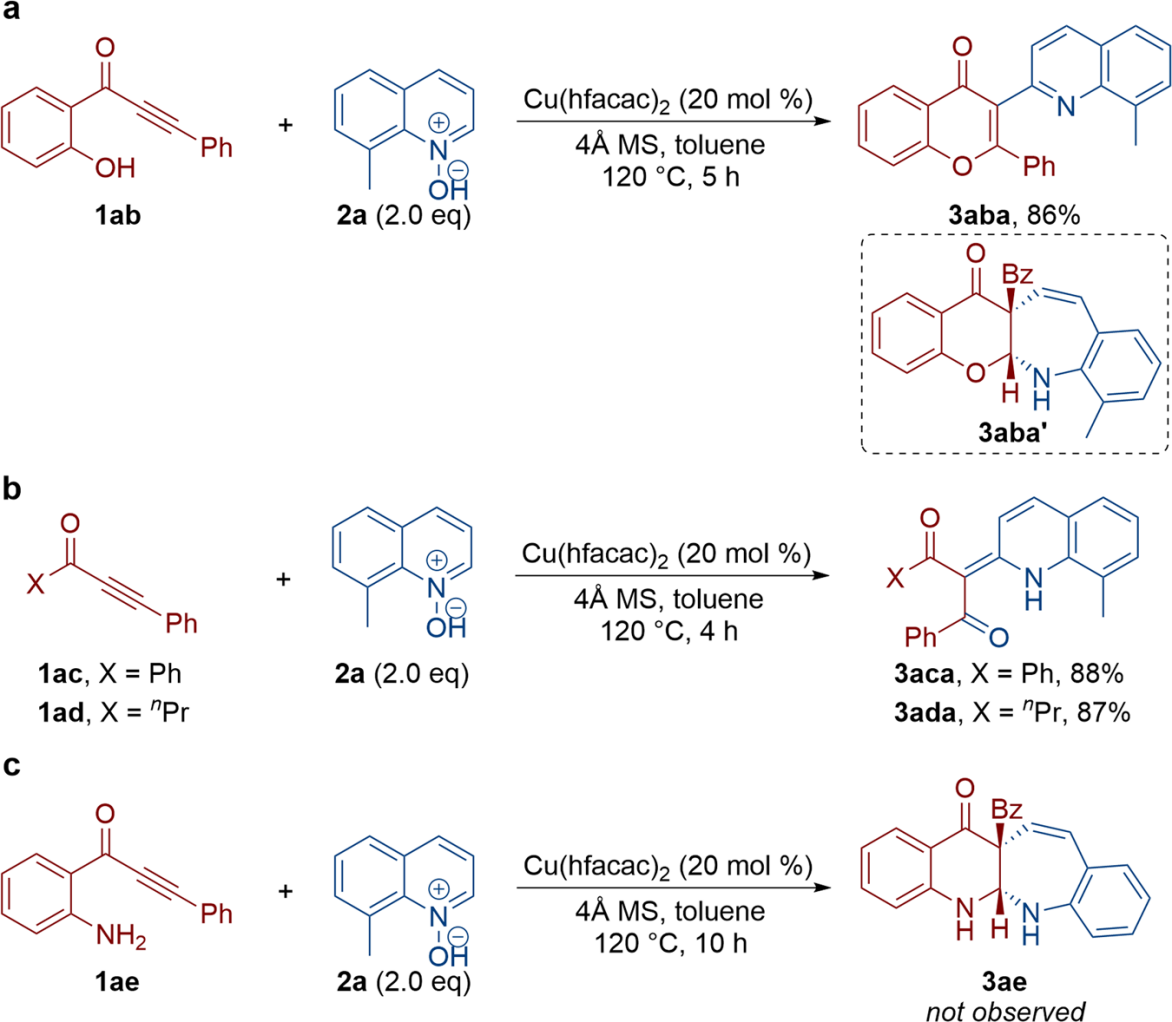
Control experiments. **a** Hydroxyl-substituted alkynone was used. **b** Unsubstituted alkynone was used. **c** Alkynone without Boc at the amino group was used.

To understand the mechanism of these cyclizations, several control experiments were first conducted (Fig. 5). Our attempts to extend the reaction to hydroxyl-substituted alkynone resulted in the formation of 3-(8-methylquinolin-2-yl)-2-phenyl-4*H*-chromen-4-one **3aba** in 86% yield, and no corresponding azepine compound **3aba’** formation was detected. The molecular structure of **3aba** was further confirmed by X-ray diffraction^12^. Furthermore, when unsubstituted alkynone **1ac** and **1ad** were subjected to this copper-catalyzed oxidative cyclization, 1,3-diones **3aca** and **3ada** were generated in 88 and 87% yields, respectively. These results showed that the amino group was crucial for the formation of benzo[6,7]azepino[2,3-*b*]quinolines. Alkynone without Boc at the amino group was then examined, and no corresponding azepine compound **3ae** was obtained, likely due to coordinate and poison copper catalysts.

### Synthetic application

**Fig. 6:**
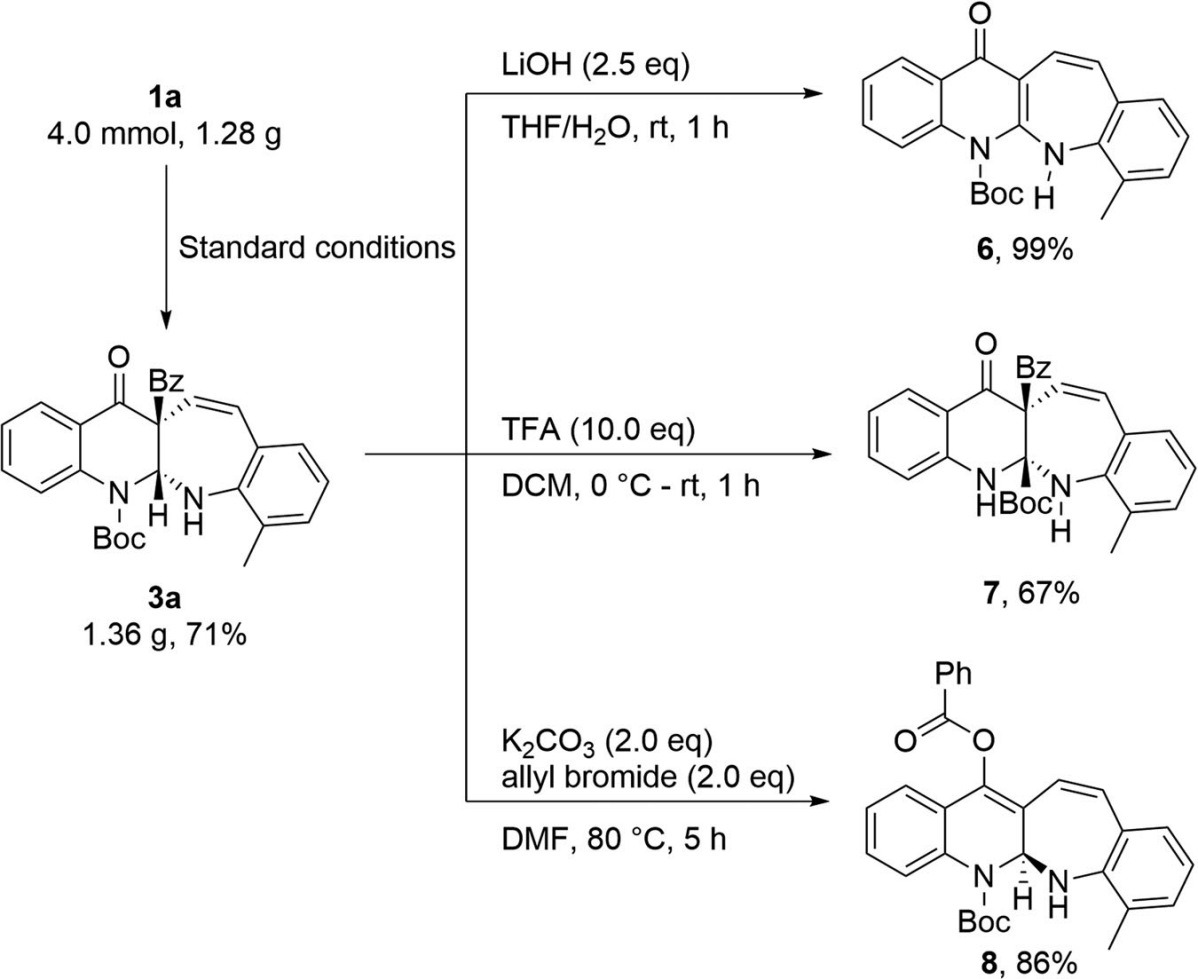
Synthetic applications. The synthetic utility of the **3a** was examined.

The synthetic utility of the benzo[6,7]azepino[2,3-*b*]quinolines was examined (Fig. 6). Firstly, **3a** was prepared on a gram scale in 71% yield under the optimized reaction conditions. Subsequently, a selective elimination of benzaldehyde of **3a** was achieved with LiOH to furnish benzo[6,7]azepino[2,3-*b*]quinoline derivatives **6** in almost quantitative yield. In addition, **3a** could be transformed into compound **7** bearing two contiguous quaternary carbon stereocenters in 67% yield via a 1,2-Boc shift. Furthermore, **3a** could be readily converted into compound **8** in 86% yield by a 1,3-benzoyl-migration. The molecular structure of **8** was further confirmed by X-ray diffraction^57^.

### Mechanistic studies

**Fig. 7:**
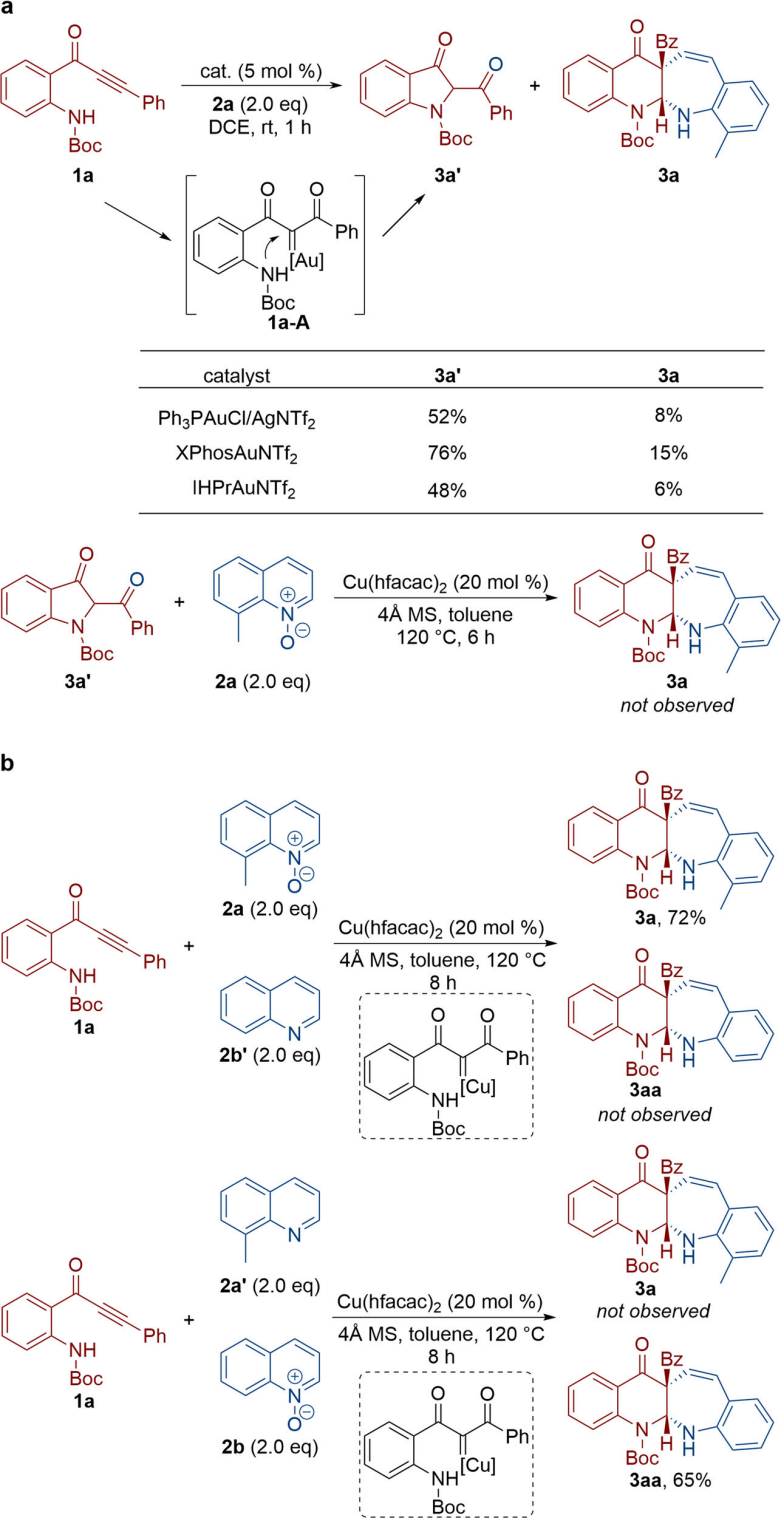
Control experiments. **a** Typical noble-metal catalysts were tested. **b** A 1:1 mixture of quinoline *N*-oxide and quinoline was treated.

Although a detailed description of mechanistic rationale at present is not possible and deserves further detailed exploration, several control experiments were conducted to gain some further information on potential pathways (Fig. 7). Typical noble-metal catalysts were tested. The direct N-H insertion by the gold carbene in **1a-A** was obtained as the main product under gold catalysis conditions. As we considered **3a’** to be possible intermediates in such a tandem sequence, we then subjected **3a’** to the optimal reaction conditions and the formation of **3a** was not observed, thus ruling out **3a’** as a potential intermediate for the formation of **3a**. Besides, we performed further studies using quinoline as the external nucleophiles. A 1:1 mixture of 8-methylquinoline *N*-oxide **2a** and quinoline **2b’** under the optimized reaction conditions only led to the formation of the corresponding **3a** in 72% yield, and no desired **3aa** was obtained. Similarly, when quinoline *N*-oxide **2b** and 8-methylquinoline **2a’** was treated under the optimized reaction conditions, only **3aa** was obtained in 65% yield. These results indicate that α-oxo copper carbene is not presumably involved in such a copper catalysis.

### Proposed mechanism

**Fig. 8:**
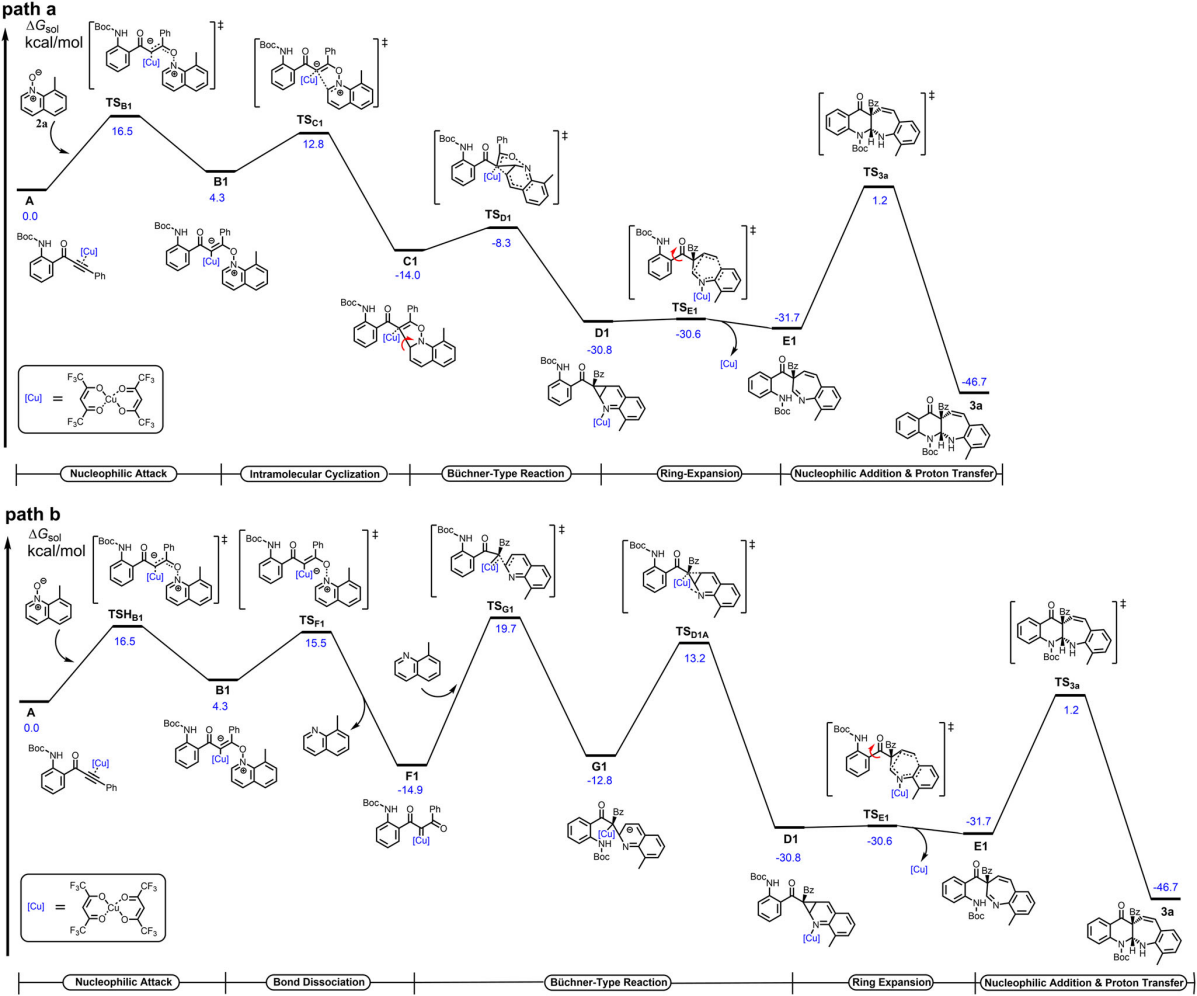
Plausible reaction mechanism for the formation of 3a. **a** α-oxo copper carbene intermediate was not involved. **b** α-oxo copper carbene intermediate was involved.

Based on the above experimental observations and density functional theory (DFT) computations (for details, see the Supplementary Information), a possible mechanism to clarify the formation of **3a** is documented. As depicted in Fig. 8, there are two plausible mechanisms to rationalize the formation of **3a**. It entails an initial copper activation of alkynone **1a** in the form of complex **A**, followed by a nucleophilic attack by 8-methylquinoline *N*-oxide **2a** via the transition state **TS-B1** to furnish the vinyl copper intermediate **B1** by overcoming a barrier of 16.5 kcal/mol. In path a, the intramolecular cyclization occurs efficiently to form the five-membered-ring intermediate **C1**, via transition state **TS-C1** with an activation energy of 8.5 kcal/mol, which undergoes Büchner-type reaction to deliver the norcaradiene intermediate **D1**. It should be mentioned that the stabilization of intermediate **C1** can be attributed to the coordination of the carbonyl oxygen to the copper atom according to the calculations. Subsequently, an electrocyclic step opens the cyclopropane ring to provide the seven-membered ring intermediate **E1** via **TS–E1** with a lower activation energy of 0.2 kcal/mol. Going a step further, intramolecular nucleophilic addition of *N*-Boc to the imine moiety produces the eventual polycyclic *N*-heterocycle **3a**. The whole process is exothermal by 46.7 kcal mol^−1^ in free energy. In path b, upon N-O bond cleavage, **B1** transforms into α-oxo copper carbene intermediate **F1** via transition state **TS-F1** with a higher activation energy of 11.2 kcal/mol, and thus the formation of the α-oxo copper carbene **F1** is unfavorable. Meanwhile, considering the subsequent Büchner-type reaction, via **TS-G1** and via **TS-D1A**, the activation barriers are 34.6 and 26.0 kcal/mol, respectively. Obviously, path a is much favored kinetically over path b. Besides, path a can rationalize our control experiments in Fig. 7 in which α-oxo copper carbene is not presumably involved in such a copper catalysis.

### Optical properties

**Fig. 9:**
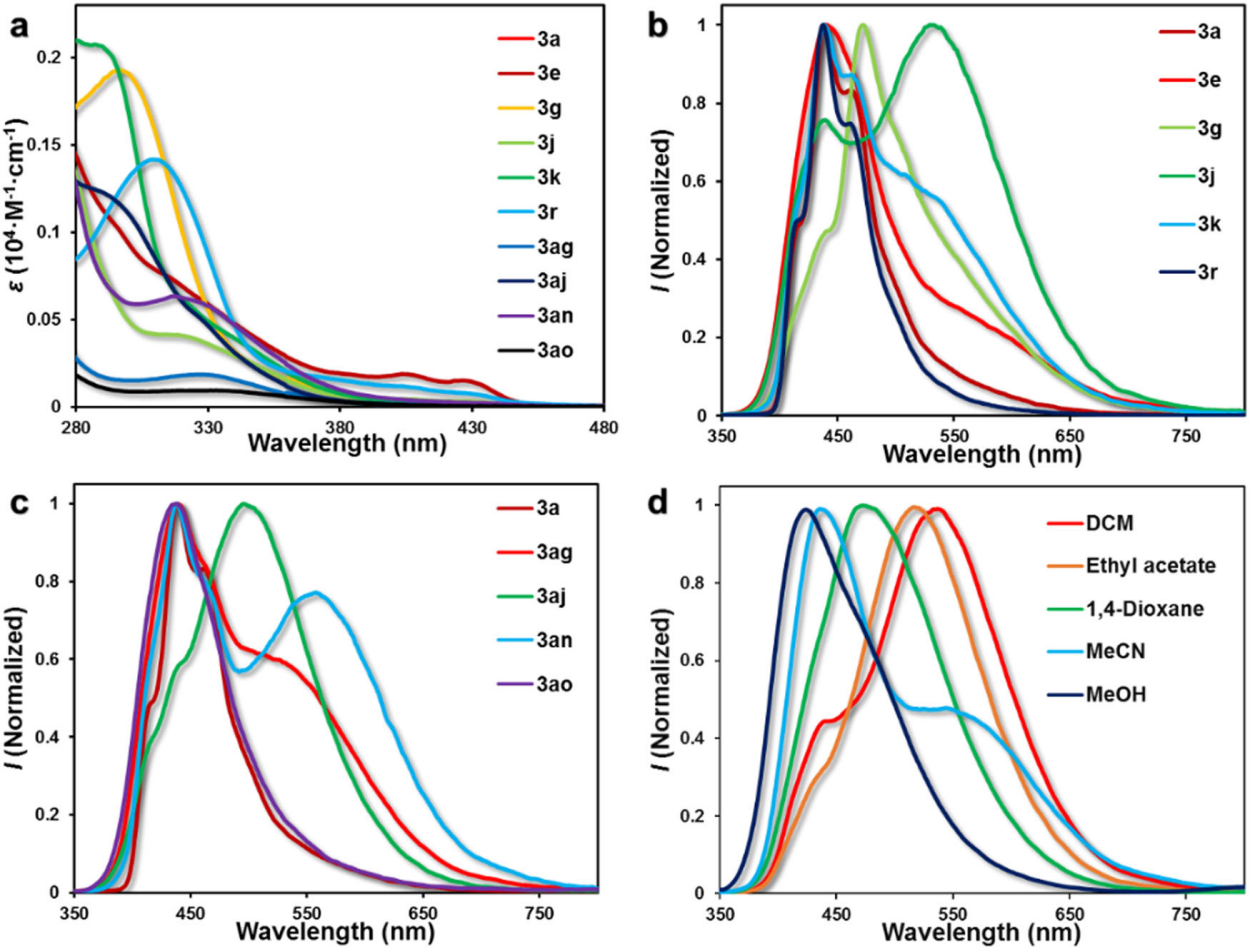
Optical properties of 3. **a** Absorption spectra of benzo[6,7]azepino[2,3-*b*]quinolines in DCM (10 μM). **b**, **c** Emission spectra of benzo[6,7]azepino[2,3-*b*]quinolines in DCM at room temperature (10 μM). **d** Emission spectra of **3r** in solvents of different polarities.

Our next efforts concentrated on the exploration of the optical properties of the obtained benzo [6,7]azepino[2,3-*b*]quinolines (Fig. 9). According to the impact of substituent on the benzene ring, the absorption and emission maxima of these compounds varied from 287 to 432 nm and from 440 to 557 nm, respectively. The absorption was red-shifted by the presence of an electron-withdrawing substituent as in **3e**, and the λ_max_ of **3e** was bathochromically shifted by 83 nm compared to **3k**. A similar effect was detected for the emission spectra of the **3ag**, which showed a longer-wavelength emission band at 536 nm and extended the emission to 750 nm. Furthermore, the fluorescence of **3g** displayed a considerable red-shift from a fluorescence maximum wavelength of 440–474 nm by conjugation with an increased benzene ring. In addition, the emission wavelengths were further red-shifted to 536 and 498 nm by the introduction of ^*t*^Bu substituent as in **3j** and **3an**. These results confirmed that the red-shifting absorption and emission band might be achieved by utilizing the strategy of combining the push–pull design, allowing the facile synthesis of near-infrared polycyclic *N*-heterocycles.

Besides, the effect of the solvent environment on their emission properties was also explored with **3r** (Fig. 9d). Interestingly, the **3r** displayed negative solvatochromism^59–61^, and the emissions of **3r** were blue-shifted from 537 to 423 nm by increasing solvent polarity (the structures of the **3r** were optimized and the dipole moment of **3r** in the excited and ground states were calculated by Gaussian 09 at the B3LYP/6-31G(d) level). The ground state of **3r** was calculated to be slightly more polar (Dipole Moment = 7.28 Debye) than the excited state (Dipole Moment = 6.45 Debye). These results supported the negative solvatochromism of **3r**. The solution fluorescence quantum yield (Φ_F_) was estimated for polycyclic *N*-heterocycles, as shown in Table 2. The Φ_F_ mainly concentrated between 0.01 and 0.05 in DCM. However, the Φ_F_ of **3r** (0.32) was much higher relative to the other compounds, presumably due to the steric effect. The two *meta*-position methyl groups limited the rotation of the benzene ring, which reduced the intramolecular vibration relaxation and improved the stability of the excited state, thus delivering the higher Φ_F_^62,63^.

**Table 2:** Photophysical data of benzo [6,7]azepino[2,3-b]quinolines^a^.

Compd	Absorption	Emission	
	*λ*_max_ (nm), *ε* (10^4^ cm^−1^M^−1^)	*λ*_max_ (nm)	Φ_F_
**3a**	309 (1.51), 329 (1.02), 403 (0.23), 428 (0.16)	440, 463	0.05
**3e**	314 (0.75), 405 (0.19), 428 (0.14)	442	0.02
**3g**	296 (1.93)	474	0.01
**3j**	318 (0.41), 404 (0.04), 431 (0.03)	441, 536	0.01
**3k**	287 (2.04), 345 (0.35)	440, 465, 537	0.04
**3r**	309 (1.15), 403 (0.12), 432 (0.07)	438, 463	0.32
**3ag**	330 (0.18)	441, 536	0.03
**3aj**	290 (1.20), 328 (0.51)	441, 498	0.03
**3an**	319 (0.63)	440, 465, 557	0.03
**3ao**	332 (0.10)	441	0.02

^a^Measured in DCM at 1.0 × 10^−5^ M, RT.

**Fig. 10:**
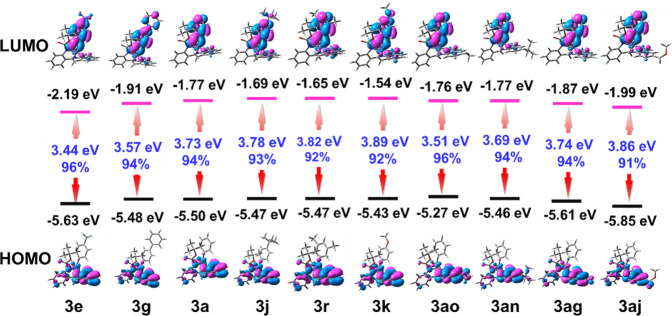
Density functional theory calculations. Molecular orbital and energy levels of benzo [6,7]azepino[2,3-*b*]quinolines, calculated at the B3LYP/6-31G(d) level.

To further explore the structure–property relationship of benzo [6,7]azepino[2,3-*b*]quinoline derivatives, DFT calculations were performed to obtain the optimized geometries and the frontier orbitals distribution of benzo [6,7]azepino[2,3-*b*]quinolines^64,65^. As shown in Fig. 10, due to the non-conjugated and non-planar heterocycle skeleton of benzo [6,7]azepino[2,3-*b*]quinolines, the Boc and benzoyl groups increased the intramolecular steric hindrance. The HOMO and LUMO levels of these compounds were well separated without significant overlap. The HOMOs of the benzo [6,7]azepino[2,3-*b*]quinolines mainly distributed on the backbone of the molecule, and the HOMO level and electron cloud distribution could be regulated by introducing different substituents into the backbone. The introduction of electron-donating substituents significantly improved the HOMO levels, such as **3ao** (−5.27 eV) to **3aj** (−5.85 eV). The LUMOs of benzo [6,7]azepino[2,3-*b*]quinolines were mainly distributed in the benzoyl groups, and the LUMO levels were regularly decreased by introducing the electron-withdrawing and conjugate groups. These results suggested that the luminescence color and band gap of the compounds could be effectively adjusted, which could have great potential applications in biological imaging and optoelectronic devices.

## Conclusions

In conclusion, we have described a copper-catalyzed dearomative annulation between alkynones and quinoline *N*-oxides, delivering the practical and efficient synthesis of fused polycyclic *N*-heterocycles. This strategy provides the first example of non-noble-metal catalyzed intermolecular alkyne oxidation via an atom-economical and environmentally friendly pathway where the quinoline partner could be further utilized to construct *N*-heterocycles instead of the previously reported the departure of a stoichiometric amount of quinolines as waste. Cyclopropanations of heteroarenes are shown in an intermolecular Büchner-type reaction, while circumventing the use of hazardous diazo carbonyl substrates. Of note, mechanistic studies revealed that this copper-catalyzed alkyne oxidation presumably proceeds by a Büchner-type ring-expansion pathway, which is distinctively different from the related gold catalysis. Moreover, such oxidation of alkynes can afford the divergent synthesis of pyridine-based diones with pyridine *N*-oxides as oxidants. Work to exploit enantioselective variants and acquire a deeper mechanistic understanding is underway.

## Methods

### Materials

Unless otherwise noted, materials were obtained commercially and used without further purification. All the solvents were treated according to general methods. Flash column chromatography was performed over silica gel (300–400 mesh). See Supplementary methods for experimental details.

### General methods

^1^H NMR spectra were recorded on a Bruker AV-400 spectrometer in chloroform-d_3_. Chemical shifts are reported in ppm with the internal TMS signal at 0.0 ppm as a standard. The data is being reported as (s = singlet, d = doublet, t = triplet, m = multiplet or unresolved, brs = broad singlet, coupling constant(s) in Hz, integration). ^13^C NMR spectra were recorded on a Bruker AV-400 spectrometer in chloroform-d_3_. Chemical shifts are reported in ppm with the internal chloroform signal at 77.0 ppm as a standard. Infrared spectra were recorded on a Nicolet iS 10 spectrometer as thin film and are reported in reciprocal centimeter (cm^−1^). Mass spectra were recorded with Micromass Q-Exactive Focus mass spectrometer using electron spray ionization. ^1^H NMR, and ^13^C NMR are supplied for all compounds: see Supplementary Figs. 1–68. More mechanism studies are supplied: see Supplementary Figs. 69–74. Representative synthetic procedures for the preparation of alkynones are supplied: see Supplementary Fig. 75. General procedure for the synthesis of benzo[6,7]azepino[2,3-*b*]quinolines **3** is supplied: see Supplementary Fig. 76. General procedure for the synthesis of pyridine-based diones **5** are supplied: see Supplementary Fig. 77. Synthetic applications are supplied: see Supplementary Figs. 78–80. Crystal data are supplied: see Supplementary Tables 1–5. TD-DFT computational data are supplied: see Supplementary Tables 6–15. See Supplementary methods for the characterization data of compounds not listed in this part.

### General procedure for the synthesis of benzo[6,7]azepino[2,3-*b*]quinolines 3

Quinoline *N*-oxide **2** (0.4 mmol), 4 Å molecular sieves (40 mg) and Cu(hfacac)_2_ (0.04 mmol, 19.1 mg) were added in this order to the alkynones **1** (0.2 mmol) in toluene (4.0 mL) at room temperature. The reaction mixture was stirred at 120 °C (120 °C, heating mantle temperature) and the progress of the reaction was monitored by TLC. Upon completion, the mixture was then concentrated and the residue was purified by chromatography on silica gel (eluent: petroleum ether/ethyl acetate) to afford the desired product **3**.

### General procedure for the synthesis of pyridine-based diones 5

2-Chloropyridine *N*-oxide **4a** (0.4 mmol, 51.8 mg) and Cu(hfacac)_2_ (0.04 mmol, 19.1 mg) were added in this order to the alkynones **1** (0.2 mmol) in toluene (4.0 mL) at room temperature. The reaction mixture was stirred at 120 °C (120 °C, heating mantle temperature) and the progress of the reaction was monitored by TLC. Upon completion, the mixture was then concentrated and the residue was purified by chromatography on silica gel (eluent: petroleum ether/ethyl acetate) to afford the desired product **5**.

## Supplementary information

Supplementary Information

Description of Additional Supplementary Files

Supplementary Data 1

Supplementary Data 2

Supplementary Data 3

Supplementary Data 4

Supplementary Data 5

Supplementary Data 6

Supplementary Data 7

## Data Availability

The authors declare that the data supporting the findings of this study are available within the article and the Supplementary Information as well as from the authors upon reasonable request. DFT calculations are available in Supplementary Data 1. The compound characterizations are available in Supplementary Data 2. The X-ray crystallographic coordinates for structures **3a**, **3r**, **3aba**, **5p**, and **8**, reported in this study have been deposited at the Cambridge Crystallographic Data Centre (CCDC), under CCDC 2122556 (**3a**, Supplementary Data 3), 2126067 (**3r**, Supplementary Data 4), 2122557 (**3aba**, Supplementary Data 5), 2144833 (**5p**, Supplementary Data 6) and 2122558 (**8**, Supplementary Data 7), respectively. These data can be obtained free of charge from The Cambridge Crystallographic Data Centre via http://www.ccdc.cam.ac.uk/data_request/cif.

## References

[1] Schultz C (1999). Paullones, a series of cyclin-dependent kinase inhibitors: synthesis, evaluation of CDK1/Cyclin B inhibition, and in vitro antitumor activity. J. Med. Chem..

[2] Sharma SK, Sharma S, Agarwal PK, Kundu B (2009). Application of 7-endo-trig Pictet–Spengler cyclization to the formation of the benzazepine ring: synthesis of benzazepinoindoles. Eur. J. Org. Chem..

[3] Magolan J, Kerr MA (2006). Expanding the scope of Mn(OAc)_3_-mediated cyclizations: synthesis of the tetracyclic core of tronocarpine. Org. Lett..

[4] Wang L-L (2020). Chalcone-based pyridinium salts and their diastereoselective dearomatization to access bibridged benzoazepines. Org. Lett..

[5] Watson MD, Fechtenkötter A, Müllen K (2001). Big is beautiful−“aromaticity” revisited from the viewpoint of macromolecular and supramolecular benzene chemistry. Chem. Rev..

[6] Anthony JE (2006). Functionalized acenes and heteroacenes for organic electronics. Chem. Rev..

[7] Georgakilas V, Perman JA, Tucek J, Zboril R (2015). Broad family of carbon nanoallotropes: classification, chemistry, and applications of fullerenes, carbon dots, nanotubes, graphene, nanodiamonds, and combined superstructures. Chem. Rev..

[8] Yet L (2000). Metal-mediated synthesis of medium-sized rings. Chem. Rev..

[9] Nubbemeyer U (2001). Synthesis of medium-sized ring lactams. Top. Curr. Chem..

[10] Nakayama H, Harada S, Kono M, Nemoto T (2017). Chemoselective asymmetric intramolecular dearomatization of phenols with α-diazoacetamides catalyzed by silver phosphate. J. Am. Chem. Soc..

[11] Ji K, Zhang L (2018). Cyclopropanation of benzene rings by oxidatively generated α-oxo gold carbene: one-pot access to tetrahydropyranone-fused cycloheptatrienes from propargyl benzyl ethers. Adv. Synth. Catal..

[12] Zhu D, Cao T, Chen K, Zhu S (2022). Rh_2_(II)-catalyzed enantioselective intramolecular Büchner reaction and aromatic substitution of donor–donor carbenes. Chem. Sci..

[13] Yuan D-F (2022). Hypervalent iodine promoted the synthesis of cycloheptatrienes and cyclopropanes. Chem. Sci..

[14] Zeng Q (2019). Divergent construction of macrocyclic alkynes via catalytic metal carbene C(sp^2^)–H insertion and the buchner reaction. ACS Catal..

[15] Zheng Z (2021). Homogeneous gold-catalyzed oxidation reactions. Chem. Rev..

[16] Ye L-W (2021). Nitrene transfer and carbene transfer in gold catalysis. Chem. Rev..

[17] Zhang L (2014). A non-diazo approach to α-oxo gold carbenes via gold-catalyzed alkyne oxidation. Acc. Chem. Res..

[18] Yeom H-S, Shin S (2014). Catalytic access to α-oxo gold carbenes by N-O bond oxidants. Acc. Chem. Res..

[19] Dorel R, Echavarren AM (2015). Gold(I)-catalyzed activation of alkynes for the construction of molecular complexity. Chem. Rev..

[20] Qian D, Zhang J (2015). Gold-catalyzed cyclopropanation reactions using a carbenoid precursor toolbox. Chem. Soc. Rev..

[21] Shen W-B, Tang X-T (2019). Recent advances in catalytic asymmetric intermolecular oxidation of alkynes. Org. Biomol. Chem..

[22] Ru G-X (2021). Recent progress towards the transition-metal-catalyzed Nazarov cyclization of alkynes via metal carbenes. Org. Biomol. Chem..

[23] Tang X-T (2020). Recent progress in N-heterocyclic carbene gold-catalyzed reactions of alkynes involving oxidation/amination/cycloaddition. Catalysts.

[24] Huple DB, Ghorpade S, Liu R-S (2016). Recent advances in gold-catalyzed N- and O-functionalizations of alkynes with nitrones, nitroso, nitro and nitroxy species. Adv. Synth. Catal..

[25] Xiao J, Li X (2011). Gold α-oxo carbenoids in catalysis: catalytic oxygen-atom transfer to alkynes. Angew. Chem. Int. Ed..

[26] Bhunia S, Ghosh P, Patra SR (2020). Gold-catalyzed oxidative alkyne functionalization by N-O/S-O/C-O bond oxidants. Adv. Synth. Catal..

[27] Ye L, Cui L, Zhang G, Zhang L (2010). Alkynes as equivalents of α-diazo ketones in generating oxo metal carbenes: a gold-catalyzed expedient synthesis of dihydrofuran-3-ones. J. Am. Chem. Soc..

[28] Liu R (2013). Generation of rhodium(I) carbenes from ynamides and their reactions with alkynes and alkenes. J. Am. Chem. Soc..

[29] Nösel P (2013). 1,6-Carbene transfer: gold-catalyzed oxidative diyne cyclizations. J. Am. Chem. Soc..

[30] Wang T (2014). Synthesis of highly substituted 3-formylfurans by a gold(I)-catalyzed oxidation/1,2-alkynyl migration/cyclization cascade. Angew. Chem. Int. Ed..

[31] Vasu D (2011). Gold-catalyzed oxidative cyclization of 1,5-enynes using external oxidants. Angew. Chem. Int. Ed..

[32] Bhunia S, Ghorpade S, Huple DB, Liu R-S (2012). Gold-catalyzed oxidative cyclizations of cis-3-en-1-ynes to form cyclopentenone derivatives. Angew. Chem. Int. Ed..

[33] Ghorpade S, Su M-D, Liu R-S (2013). Gold-catalyzed oxidative cyclizations on 1,4-enynes: evidence for a γ-substituent effect on Wagner-Meerwein rearrangements. Angew. Chem. Int. Ed..

[34] Karad SN, Liu R-S (2014). Gold-catalyzed 1,2-oxoarylations of nitriles with pyridine-derived oxides. Angew. Chem. Int. Ed..

[35] Li L (2014). Generation of gold carbenes in water: efficient intermolecular trapping of the α-oxo gold carbenoids by indoles and anilines. Chem. Sci..

[36] Shu C (2019). Generation of endocyclic vinyl carbene complexes via gold-catalyzed oxidative cyclization of terminal diynes: toward naphthoquinones and carbazolequinones. ACS Catal..

[37] Hamada N, Yamaguchi A, Inuki S, Oishi S, Ohno H (2018). Gold(I)-catalyzed oxidative cascade cyclization of 1,4-diyn-3-ones for the construction of tropone-fused furan scaffolds. Org. Lett..

[38] Qian D (2014). Gold(I)-catalyzed highly diastereo-and enantioselective alkyne oxidation/cyclopropanation of 1,6-enynes. Angew. Chem. Int. Ed..

[39] Wang A, Lu M, Liu Y (2021). Gold-catalyzed oxidative cyclization involving nucleophilic attack to the keto group of α,α′-dioxo gold carbene and 1,2-alkynyl migration: synthesis of furan-3-carboxylates. Org. Lett..

[40] Zhao J (2018). Gold-catalyzed oxidative cyclizations of {o-(alkynyl)phenyl propargyl} silyl ether derivatives involving 1,2-enynyl migration: synthesis of functionalized 1H-isochromenes and 2H-pyrans. Org. Lett..

[41] Ji K, Liu X, Du B, Yang F, Gao J (2015). Gold-catalyzed selective oxidation of 4-oxahepta-1,6-diynes to 2H-pyran-3(6H)-ones and chromen-3(4H)-ones via β-gold vinyl cation intermediates. Chem. Commun..

[42] Li J, Xing H-W, Yang F, Chen Z-S, Ji K (2018). Gold(III)-catalyzed regioselective oxidation/cycloisomerization of diynes: an approach to fused Furan derivatives. Org. Lett..

[43] Wagh SB, Singh RR, Sahani RL, Liu R-S (2019). Gold-catalyzed oxidative hydrative alkenylations of propargyl aryl thioethers with quinoline N‑oxides involving a 1,3-sulfur migration. Org. Lett..

[44] Nan J (2021). Cu^II^-catalyzed coupling with two ynone units by selective triple and sigma C−C and C−H bond cleavages. Org. Lett..

[45] Chen Z-S (2016). Metal-free, site-selective addition to ynones: an approach to synthesize substituted quinoline derivatives. Org. Lett..

[46] Zhang S, Wu C, Zhang Z, Wang T (2019). Metal-free synthesis of 3‑(iso)quinolinyl 4‑chromenones and 3‑(iso)quinolinyl 4-quinolones from (iso)quinoline N‑oxides and ynones. Org. Lett..

[47] Liu J, Ba D, Chen Y, Wen S, Cheng G (2020). Synthesis of 3-(2-quinolyl) chromones from ynones and quinoline N-oxides via tandem reactions under transition metal- and additive-free conditions. Chem. Commun..

[48] Park CP, Nagle A, Yoon CH, Chen C, Jung KW (2009). Formal aromatic C−H insertion for stereoselective isoquinolinone synthesis and studies on mechanistic insights into the C−C bond formation. J. Org. Chem..

[49] Mo S, Xu J (2014). Chemospecific intramolecular Buchner reaction catalyzed by copper(II) acetylacetonate. ChemCatChem.

[50] Ru G-X (2022). Copper catalyzed dearomatization by Michael-type addition of indolyl ynones: divergent synthesis of functionalized spiroindoles and cyclopenta[c]quinolin-3-ones. Org. Chem. Front..

[51] Shen W-B (2021). Copper(I)-catalyzed enyne oxidation/cyclopropanation: divergent and enantioselective synthesis of cyclopropanes. Org. Lett..

[52] Shen W-B (2021). Cu(I)- and Au(I)-catalyzed regioselective oxidation of diynes: divergent synthesis of N-heterocycles. Org. Chem. Front..

[53] Shen W-B (2020). Cu(I)-catalyzed oxidative cyclization of enynamides: regioselective access to cyclopentadiene frameworks and 2-aminofurans. Org. Lett..

[54] Zheng Y, Zhang T-T, Shen W-B (2021). Gold-catalyzed oxidative cyclization of amide-alkynes: access to functionalized γ-lactams. Org. Biomol. Chem..

[55] Shen W-B (2017). Highly site selective formal [5+2] and [4+2] annulations of isoxazoles with heterosubstituted alkynes by platinum catalysis: rapid access to functionalized 1,3-oxazepines and 2,5-dihydropyridines. Angew. Chem. Int. Ed..

[56] Shen W-B (2017). Divergent synthesis of N-heterocycles via controllable cyclization of azido-diynes catalyzed by copper and gold. Nat. Commun..

[57] CCDC 2122556 (**3a**), CCDC 2126067 (**3r**), CCDC 2122557 (**3aba**), CCDC 2144833 (**5p**), and CCDC 212255 (**7**) contain the supplementary crystallographic data for this paper. These data can be obtained free of charge from the Cambridge Crystallographic Data Centre.

[58] Lu B, Li C, Zhang L (2010). Gold-catalyzed highly regioselective oxidation of C-C triple bonds without acid additives: propargyl moieties as masked α,β-unsaturated carbonyls. J. Am. Chem. Soc..

[59] Kim, J. J. et al. Negative solovatochromism of azo dyes derived from (dialkylamino)thiazole dimers. *Chem. Commun*. 753–754 (2000).

[60] Iuliano V (2019). Negative solvatochromism in a *N*-linked *p*-pyridiniumcalix[4]arene derivative. Org. Lett..

[61] Sharma R (2016). High singlet oxygen production and negative solvatochromism of octabrominated 3-pyrrolyl boron dipyrromethenes. RSC Adv..

[62] Zhao Z, Zhang H, Lam JWY, Tang BZ (2020). Synergistic N-heterocyclic carbene/palladium-catalyzed umpolung 1,4-addition of aryl iodides to enals. Angew. Chem. Int. Ed..

[63] Tu Y, Zhao Z, Lam JWY, Tang BZ (2021). Mechanistic connotations of restriction of intramolecular motions (RIM). Nat. Sci. Rev..

[64] Cai X-M (2021). BioAIEgens derived from rosin: how does molecular motion affect their photophysical processes in solid state?. Nat. Commun..

[65] Liu, D., et al. Molecular core–shell structure design: facilitating delayed fluorescence in aggregates toward highly efficient solution-processed OLEDs. *Aggregate* **3**, e164 (2022).

