# Supplementary material for: Copper-catalyzed alkyne oxidation/Büchner-type ring-expansion to access benzo[6,7]azepino[2,3-b]quinolines and pyridine-based diones
Source: Commun Chem. 2023 Feb 20;6:35. doi: 10.1038/s42004-023-00840-6 (PMC9941089; doi:10.1038/s42004-023-00840-6)
Supplement: Supplementary file 1 — Supplementary Information [file 42004_2023_840_MOESM1_ESM.pdf]

Supplementary Table 1. Crystal data and structure refinement for 3a. CCDC Number = 2122556

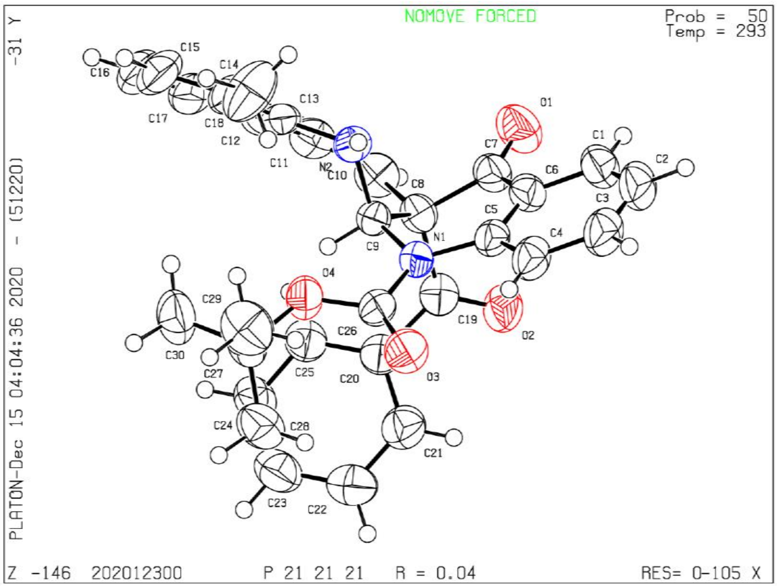

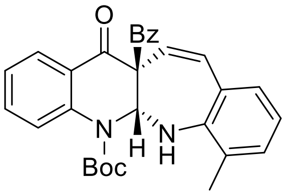

Bond precision: C-C = 0.0054 A Wavelength=1.54184

Cell:	a=11.8047(11)	b=13.4572(15)	c=16.0448(16)
	alpha=90	beta=90	gamma=90
Temperature:	293 K		

	Calculated	Reported
Volume	2548.9(4)	2548.9(4)
Space group	P 21 21 21	P 21 21 21
Hall group	P 2ac 2ab	P 2ac 2ab
Moiety formula	C30 H28 N2 O4	C30 H28 N2 O4
Sum formula	C30 H28 N2 O4	C30 H28 N2 O4
Mr	480.54	480.54
Dx,g cm-3	1.252	1.252
Z	4	4
Mu (mm-1)	0.671	0.671
F000	1016.0	1016.0
F000'	1019.06	
h,k,lmax	14,16,19	14,16,19
Nref	4545[2576]	4546
Tmin,Tmax	0.901,0.935	0.926,1.000
Tmin'	0.892	

Correction method= # Reported T Limits: Tmin=0.926 Tmax=1.000
AbsCorr = MULTI-SCAN

Data completeness= 1.76/1.00 Theta(max)= 67.076

R(reflections)= 0.0431(3686) wR2(reflections)= 0.1060(4546)

S = 1.006 Npar= 333

Supplementary Table 2. Crystal data and structure refinement for 3r. CCDC Number = 2126067

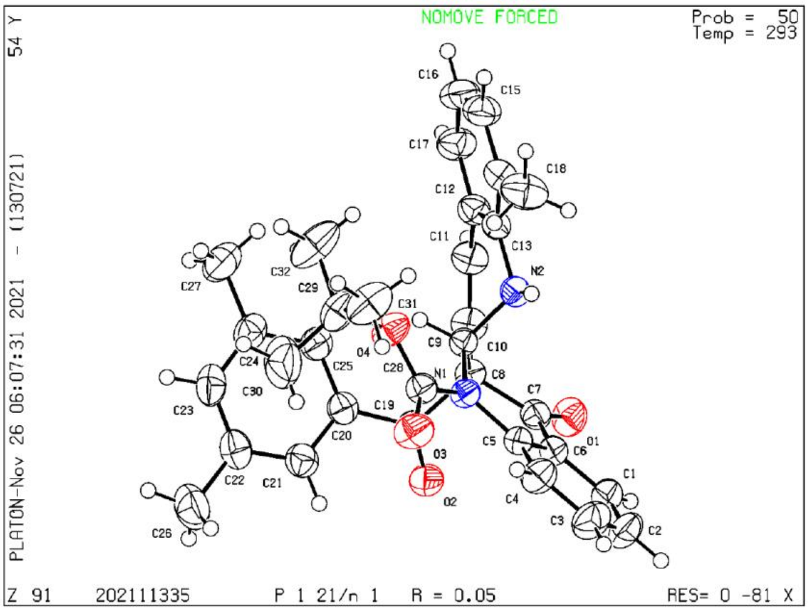

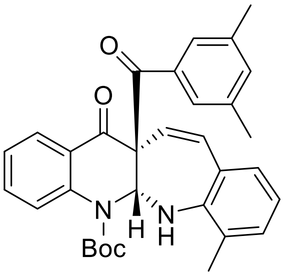

Bond precision: C-C = 0.0032 A Wavelength=1.54184

Cell:	a=10.10206(18)	b=16.9389(3)	c=16.2465(3)
	alpha=90	beta=98.0708(17)	gamma=90
Temperature:	293 K		

	Calculated	Reported
Volume	2752.53(9)	2752.53(9)
Space group	P 21/n	P 1 21/n 1
Hall group	-P 2yn	-P 2yn
Moiety formula	C32 H32 N2 O4	C32 H32 N2 O4
Sum formula	C32 H32 N2 O4	C32 H32 N2 O4
Mr	508.60	508.59
Dx,g cm-3	1.227	1.227
Z	4	4
Mu (mm-1)	0.648	0.648
F000	1080.0	1080.0
F000'	1083.19	
h,k,lmax	12,20,19	12,20,19
Nref	4916	4912
Tmin,Tmax	0.918,0.943	0.970,1.000
Tmin'	0.902	

Correction method= # Reported T Limits: Tmin=0.970 Tmax=1.000
AbsCorr = MULTI-SCAN

Data completeness= 0.999 Theta(max)= 67.078

R(reflections)= 0.0494(3903) wR2(reflections)= 0.1426(4912)

S = 1.029 Npar= 354

Supplementary Table 3. Crystal data and structure refinement for 3aba. CCDC Number = 2122557

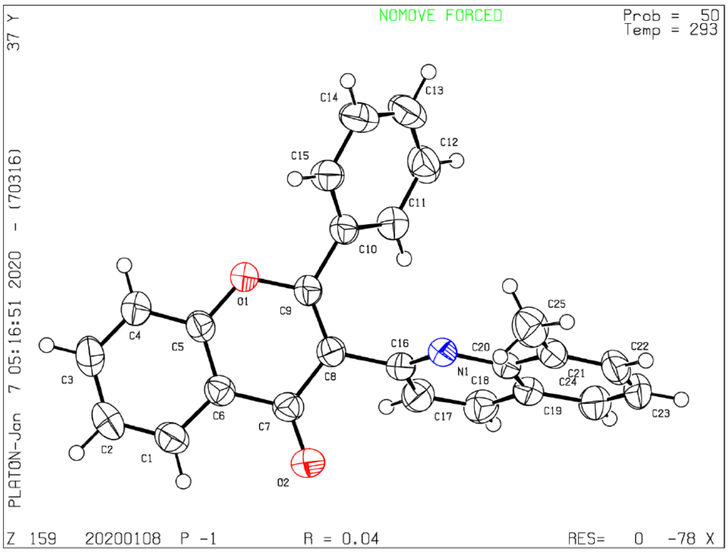

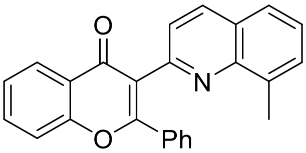

Bond precision: C-C = 0.0024 A   Wavelength=1.54184

Cell: a=8.7981(5) b=9.4244(5) c=12.4880(7)
alpha=100.906(5) beta=109.593(5) gamma=101.021(4)

Temperature: 293 K

	Calculated	Reported
Volume	920.90(10)	920.88(9)
Space group	P -1	P -1
Hall group	-P 1	-P 1
Moiety formula	C25 H17 N O2	C25 H17 N O2
Sum formula	C25 H17 N O2	C25 H17 N O2
Mr	363.40	363.39
Dx,g cm-3	1.311	1.311
Z	2	2
Mu (mm-1)	0.660	0.660
F000	380.0	380.0
F000’	381.11	
h,k,lmax	10,11,14	10,11,14
Nref	3288	3285
Tmin,Tmax	0.917,0.942	0.744,1.000
Tmin’	0.882	

Correction method= # Reported T Limits: Tmin=0.744 Tmax=1.000
AbsCorr = MULTI-SCAN

Data completeness= 0.999   Theta(max)= 67.065

R(reflections)= 0.0402(2746)   wR2(reflections)= 0.1148(3285)

S = 1.033   Npar= 254

Supplementary Table 4. Crystal data and structure refinement for 5p. CCDC Number = 2144833

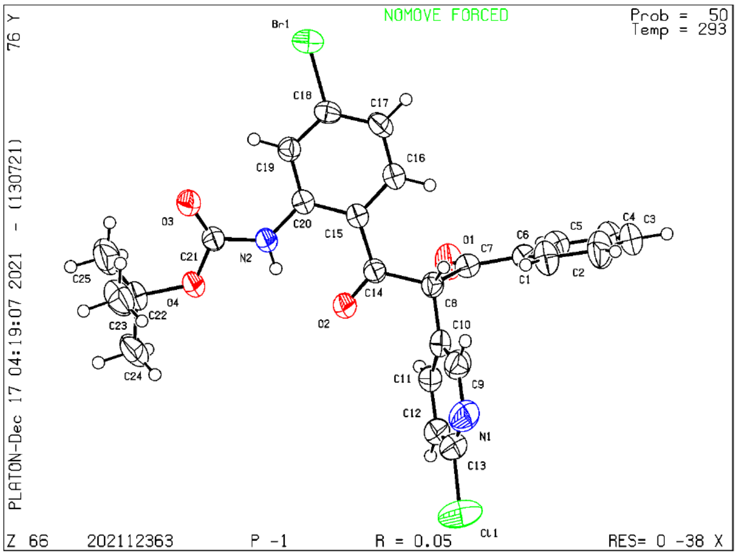

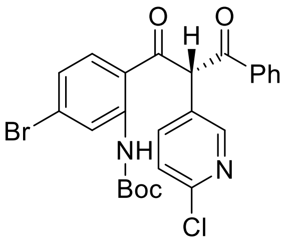

Bond precision: C-C = 0.0053 A Wavelength=1.54184

Cell:	a=6.8476(4)	b=11.6940(7)	c=15.8939(12)
	alpha=91.695(5)	beta=99.775(6)	gamma=106.165(5)
Temperature:	293 K		

	Calculated	Reported
Volume	1200.68(14)	1200.68(14)
Space group	P -1	P -1
Hall group	-P 1	-P 1
Moiety formula	C25 H22 Br Cl N2 O4	C25 H22 Br Cl N2 O4
Sum formula	C25 H22 Br Cl N2 O4	C25 H22 Br Cl N2 O4
Mr	529.80	529.80
Dx,g cm-3	1.465	1.465
Z	2	2
Mu (mm-1)	3.642	3.642
F000	540.0	540.0
F000'	540.74	
h,k,lmax	8,13,18	8,13,18
Nref	4270	4258
Tmin,Tmax	0.678,0.747	0.949,1.000
Tmin'	0.572	

Correction method= # Reported T Limits: Tmin=0.949 Tmax=1.000
AbsCorr = MULTI-SCAN

Data completeness= 0.997 Theta(max)= 67.079

R(reflections)= 0.0454(3434) wR2(reflections)= 0.1300(4258)

S = 1.024 Npar= 305

Supplementary Table 5. Crystal data and structure refinement for 8. CCDC Number = 2122558

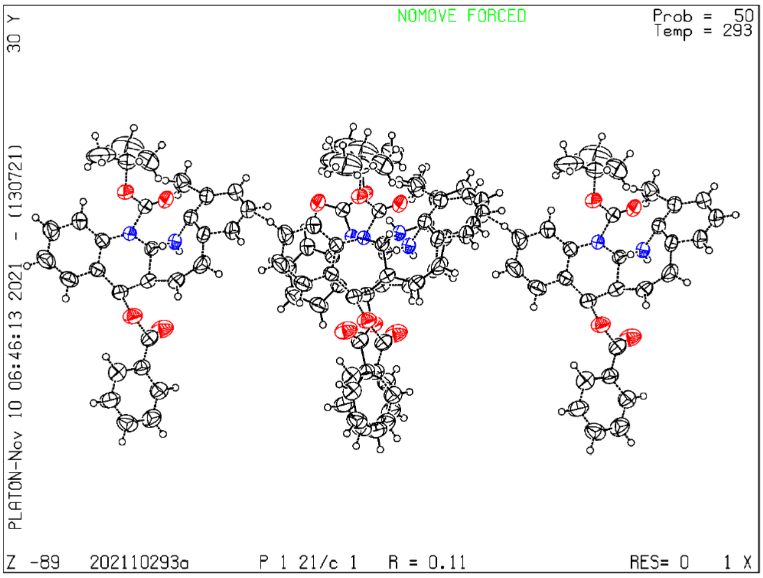

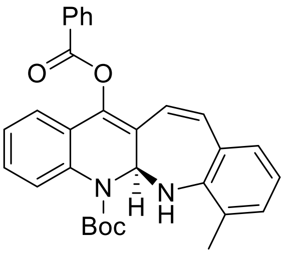

Bond precision: = 0.0000 A   Wavelength=1.54184

Cell:	a=18.9392(6)	b=10.8598(3)	c=12.7501(5)
	alpha=90	beta=94.781(3)	gamma=90
Temperature:	293 K		

	Calculated	Reported
Volume	2613.26(15)	2613.28(16)
Space group	P 21/c	P 1 21/c 1
Hall group	-P 2ybc	-P 2ybc
Moiety formula	C2.62 H2.46 N0.18 O0.30, C27.39 H25.54 N1.82 O3.70	C27.38 H25.53 N1.82 O3.7, C2.62 H2.47 N0.18 O0.3
Sum formula	C30 H28 N2 O4	C30 H28 N2 O4
Mr	480.54	480.54
Dx,g cm-3	1.221	1.221
Z	4	4
Mu (mm-1)	0.655	0.655
F000	1016.0	1016.0
F000'	1019.06	
h,k,lmax	22,12,15	22,12,15
Nref	4652	4621
Tmin,Tmax	0.924,0.974	0.983,1.000
Tmin'	0.906	

Correction method= # Reported T Limits: Tmin=0.983 Tmax=1.000
AbsCorr = MULTI-SCAN

Data completeness= 0.993   Theta(max)= 67.076

R(reflections)= 0.1056(3165)   wR2(reflections)= 0.3217(4621)

S = 1.165   Npar= 405

Supplementary Discussion

More Mechanism Studies

1. To probe the reaction mechanism, control experiments with $H_2{}^{18}O$ isotopic labeling proved that the oxygen atom in the carbonyl group of **3a** originated from *N*-oxide but not from water.

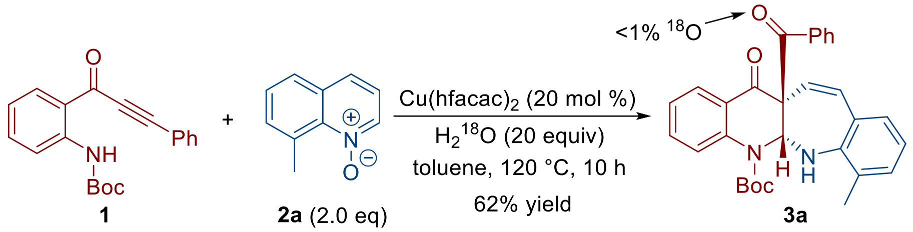

Supplementary Figure 69. The effect of water on the copper-catalyzed cascade reaction.

2. The addition of styrene did not significantly affect this cascade cyclization and no cyclopropane formation was observed under standard conditions.

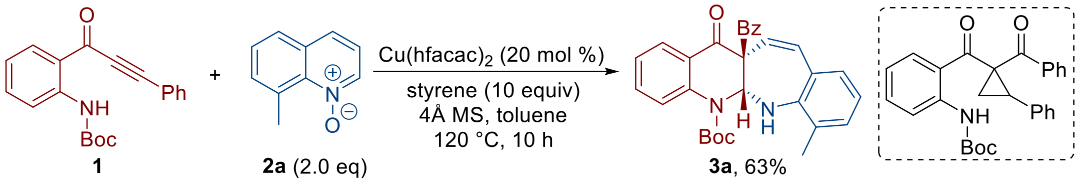

Supplementary Figure 70. Trying to trap the reaction intermediate with styrene.

3. Plausible reaction mechanistic scenario for the formation of **3a**, please see as followed.

a

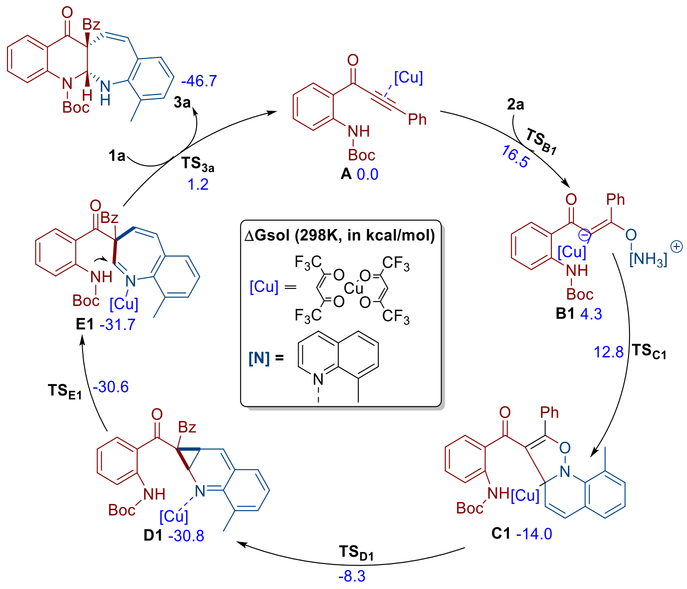

b

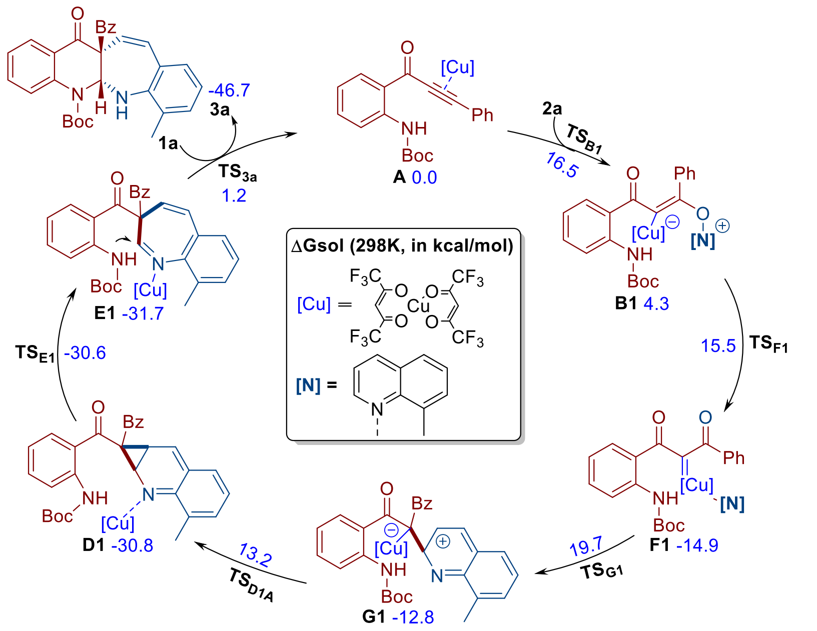

Supplementary Figure 71. Two plausible reaction pathways. **a** α-oxo copper carbene intermediate was not involved. **b** α-oxo copper carbene intermediate was involved.

4. Plausible reaction mechanism for the formation of **5a**, please see as followed.

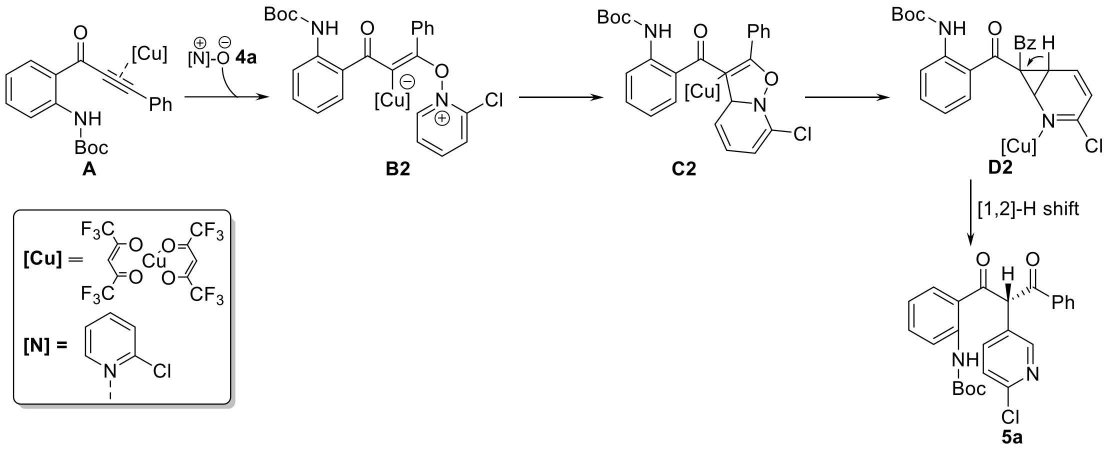

Supplementary Figure 72. Plausible reaction pathway.

5. With regard to quinazoline *N*-oxide, these two classes of reactions work to afford benzo[6,7]azepino[2,3-*b*]quinolines. With regard to pyrazine *N*-oxide, these two classes of reactions work to afford pyridine-based diones. A 1:1 mixture of quinazoline *N*-oxide and pyrazine *N*-oxide under the optimized reaction conditions mostly lead to the formation of the benzo[6,7]azepino[2,3-*b*]quinolines.

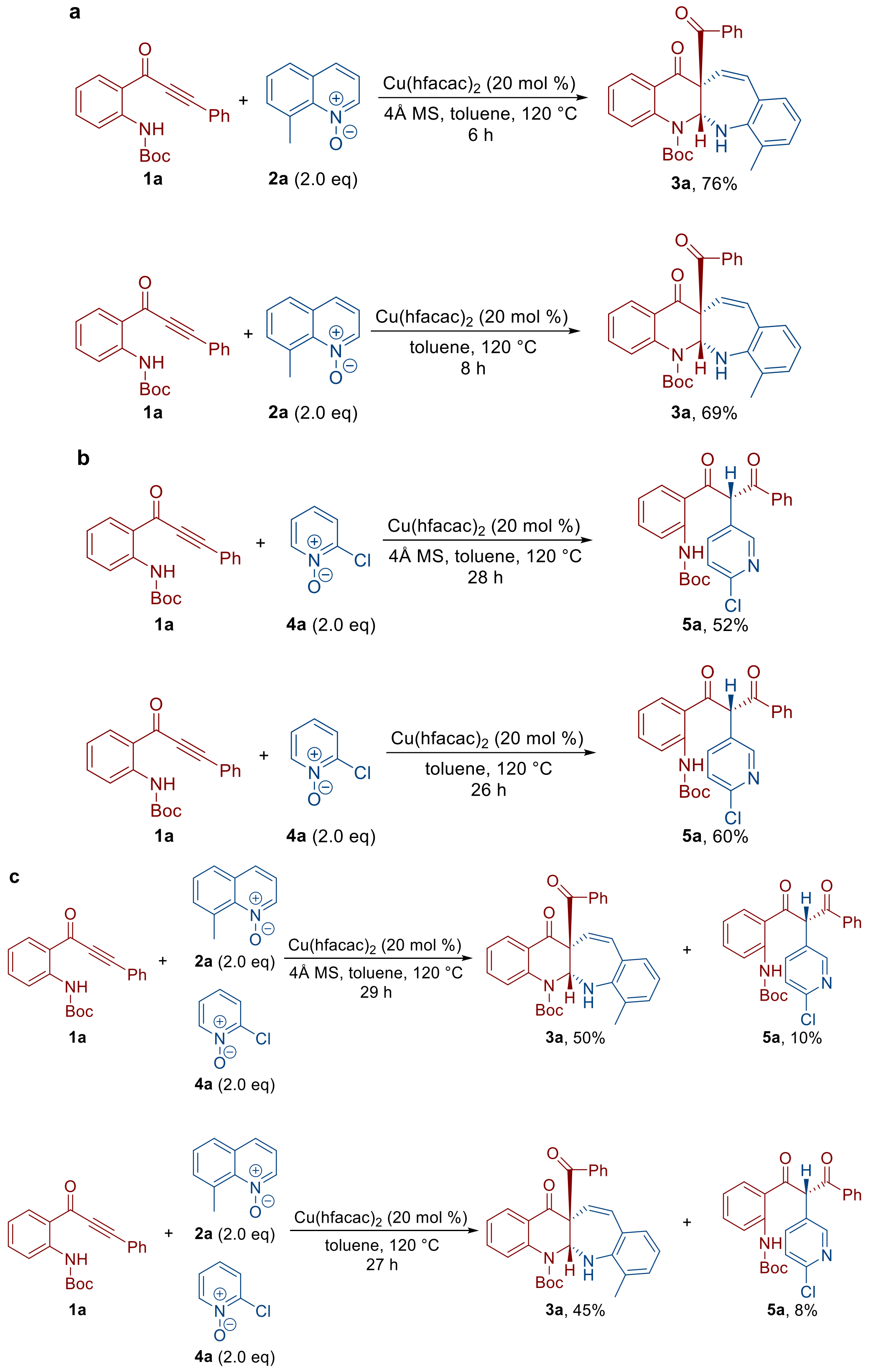

Supplementary Figure 73. Control experiments with quinazoline *N*-oxide and pyrazine *N*-oxide. **a** With regard to quinazoline *N*-oxide. **b** With regard to pyrazine *N*-oxide. **c** A 1:1 mixture of quinazoline *N*-oxide and pyrazine *N*-oxide.

6. Quinoxaline *N*-oxide and pyrazine *N*-oxide were not successful substrates and no corresponding products was observed under standard conditions, respectively.

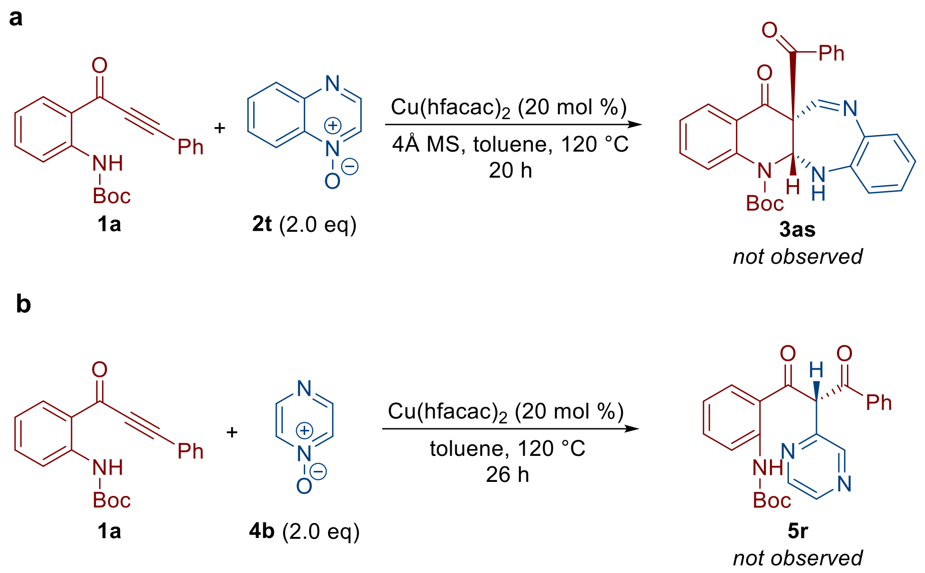

Supplementary Figure 74. Substrate expansion. **a** Quinoxaline N-oxide. **b** Pyrazine N-oxide.

Supplementary Methods

General Information. Ethyl acetate (ACS grade), hexanes (ACS grade) and anhydrous 1,2-dichloroethane (ACS grade) were obtained commercially and used without further purification. Methylene chloride, tetrahydrofuran and diethyl ether were purified according to standard methods unless otherwise noted. Commercially available reagents were used without further purification. Reactions were monitored by thin layer chromatography (TLC) using silicycle pre-coated silica gel plates. Flash column chromatography was performed over silica gel (300-400 mesh). Infrared spectra were recorded on a Nicolet iS 10 spectrometer as thin film and are reported in reciprocal centimeter (cm^{-1}). Mass spectra were recorded with Micromass Q-Exactive Focus mass spectrometer using electron spray ionization.

^{1}H NMR spectra were recorded on a Bruker AV-400 spectrometer in chloroform-d_3. Chemical shifts are reported in ppm with the internal TMS signal at 0.0 ppm as a standard. The data is being reported as (s = singlet, d = doublet, t = triplet, m = multiplet or unresolved, brs = broad singlet, coupling constant(s) in Hz, integration).

^{13}C NMR spectra were recorded on a Bruker AV-400 spectrometer in chloroform-d_3. Chemical shifts are reported in ppm with the internal chloroform signal at 77.0 ppm as a standard.

Experimental Section

Representative synthetic procedures for the preparation of alkynones 1a-1z:

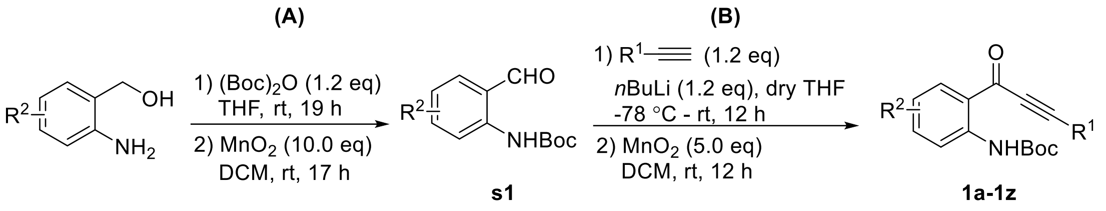

Supplementary Figure 75. Representative synthetic procedures for the preparation of ynamides **1** (**1a-1z**).

(A): $(Boc)_2O$ (12.0 mmol, 2616.0 mg) was slowly added to a solution of 2-aminobenzyl alcohol (10.0 mmol) in 20.0 mL THF at room temperature. The reaction mixture was stirred at room temperature and the progress of the reaction was monitored by TLC. The reaction typically took 19 h. Upon completion, the reaction crude was concentrated under vacuum, and the residue was added into a solution of the MnO_2 (100.0 mmol, 8600.0 mg) in DCM (20.0 mL) at room temperature. The reaction mixture was stirred at room temperature and the progress of the reaction was monitored by TLC. The reaction typically took 17 h. Upon completion, the reaction crude was filtered through a Celite plug and concentrated under vacuum, and the residue was purified by chromatography on silica gel (eluent: petroleum ether/ethyl acetate) to afford the desired substrates **s1**.[1]

(B): To a solution of acetylene (7.2 mmol) in anhydrous THF was added *n*-BuLi (7.2 mmol, 3.0 mL) at -78 °C under an Ar atmosphere. The resulting solution was stirred at -78 °C for 1 h. Then **s1** (6.0 mmol) in anhydrous THF was added by syringe. The reaction mixture was kept under the inert atmosphere and stirred for 12 h while it warmed to ambient temperature. Upon completion, the mixture was then quenched by adding satd aq NH_4Cl and the mixture was extracted with ethyl acetate (3 x 30.0 mL). The combined organic layer was washed with brine, dried over $MgSO_4$, and concentrated under reduced pressure. The residue was added into a solution of MnO_2 (30.0 mmol, 2580.0 mg) in DCM (20.0 mL) at room temperature. The reaction

mixture was stirred at room temperature and the progress of the reaction was monitored by TLC. The reaction typically took 12 h. Upon completion, the reaction crude was filtered through a Celite plug and concentrated under vacuum, and the residue was purified by chromatography on silica gel (eluent: petroleum ether/ethyl acetate) to afford the desired alkynone substrates **1a-1z.**[2]

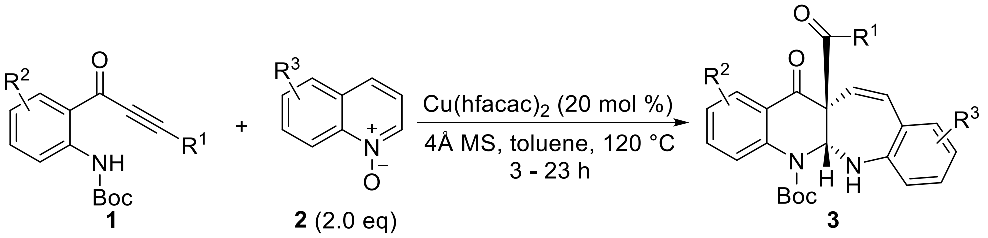

Supplementary Figure 76. Synthesis of benzo[6,7]azepino[2,3-*b*]quinolines **3**.

General procedure for the synthesis of benzo[6,7]azepino[2,3-*b*]quinolines 3: Quinoline *N*-oxide **2** (0.4 mmol), 4Å molecular sieves (40 mg) and Cu(hfacac)$_2$ (0.04 mmol, 19.1 mg) were added in this order to the alkynones **1** (0.2 mmol) in toluene (4.0 mL) at room temperature. The reaction mixture was stirred at 120 ^{o}C (120 °C, heating mantle temperature) and the progress of the reaction was monitored by TLC. Upon completion, the mixture was then concentrated and the residue was purified by chromatography on silica gel (eluent: petroleum ether/ethyl acetate) to afford the desired product **3**.

***tert*-butyl 11a-benzoyl-4-methyl-11-oxo-5,5a,11,11a-tetrahydro-6*H*-benzo[6,7]azepino[2,3-*b*]quinoline-6-carboxylate (3a)**

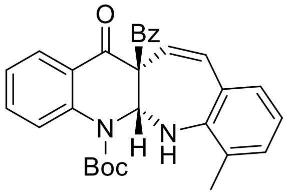

3a

The reaction was conducted with *tert*-butyl (2-(3-phenylpropioloyl)phenyl)carbamate (**1a**, 0.2 mmol, 64.2 mg), 8-methylquinoline *N*-oxide (**2a**, 0.4 mmol, 63.6 mg), 4Å

molecular sieves (40 mg) and Cu(hfacac)$_2$ (0.04 mmol, 19.1 mg) in toluene (4.0 mL) at 120 ^{o}C (120 °C, heating mantle temperature). Purification by column chromatography on silica gel (petroleum ether/ethyl acetate = 5:1) yielded **3a** (73.1 mg, 76%) as a yellow solid (mp 147-149 °C).

The reaction was conducted with *tert*-butyl (2-(3-phenylpropioloyl)phenyl)carbamate (**1a**, 4.0 mmol, 1284.0 mg), 8-methylquinoline *N*-oxide (**2a**, 8.0 mmol, 1272.0 mg), 4Å molecular sieves (400 mg) and Cu(hfacac)$_2$ (0.8 mmol, 382.2 mg) in toluene (40.0 mL) at 120 ^{o}C (120 °C, oil bath temperature). Purification by column chromatography on silica gel (petroleum ether/ethyl acetate = 5:1) yielded **3a** (1362.9 mg, 71%).

^{1}H NMR (400 MHz, CDCl$_3$) δ 8.08 (d, *J* = 7.8 Hz, 1H), 8.01 – 7.94 (m, 3H), 7.54 – 7.50 (m, 2H), 7.40 – 7.36 (m, 2H), 7.20 – 7.15 (m, 2H), 7.02 (d, *J* = 7.3 Hz, 1H), 6.87 – 6.80 (m, 2H), 6.34 (s, 1H), 6.20 (d, *J* = 12.4 Hz, 1H), 3.97 (s, 1H), 2.11 (s, 3H), 1.19 (s, 9H); ^{13}C NMR (100 MHz, CDCl$_3$) δ 195.0, 191.5, 151.1, 144.0, 139.2, 135.9, 134.4, 133.3, 132.5, 131.9, 130.2, 129.3, 128.6, 128.3, 124.6, 124.4, 123.0, 122.5, 121.3(4), 121.2(6), 120.9, 83.2, 72.4, 71.1, 27.7, 17.6; IR (neat): 3368(br), 2921, 2851, 1725, 1705, 1699, 1597, 1470, 1369, 1305, 1156, 1145, 1021, 937, 766; HRMS (ESI) m/z: [M + H]$^+$ calcd for $C_{30}H_{29}N_2O_4$ 481.2122, found 481.2115.

***tert*-butyl 2-benzoyl-3-oxoindoline-1-carboxylate (3a')**

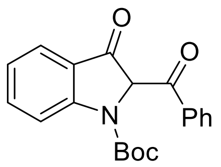

3a'

^{1}H NMR (400 MHz, CDCl$_3$) δ 8.36 (d, *J* = 8.4 Hz, 1H), 8.11 (d, *J* = 7.5 Hz, 2H), 7.69 – 7.65 (m, 3H), 7.56 – 7.53 (m, 2H), 7.15 (t, *J* = 7.3 Hz, 1H), 5.96 (s, 1H), 1.34 (s, 9H); ^{13}C NMR (100 MHz, CDCl$_3$) δ 190.4, 188.9, 154.3, 149.8, 137.7, 134.7, 134.0, 129.6, 128.8, 124.6, 123.2, 116.5, 82.8, 71.2, 27.9; IR (neat): 2928, 1724, 1701, 1650,

1450, 1326, 1150, 795; HRMS (ESI) m/z: $[M + H]^+$ calcd for $C_{20}H_{20}NO_4$ 338.1387, found 338.1387.

***tert*-butyl 11a-(4-fluorobenzoyl)-4-methyl-11-oxo-5,5a,11,11a-tetrahydro-6*H*-benzo[6,7]azepino[2,3-*b*]quinoline-6-carboxylate (3b)**

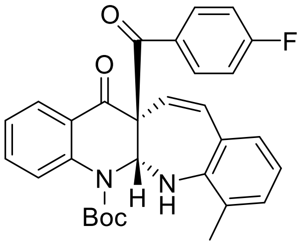

3b

The reaction was conducted with *tert*-butyl (2-(3-(4-fluorophenyl)propioloyl)phenyl)carbamate (**1b**, 0.2 mmol, 67.8 mg), 8-methylquinoline *N*-oxide (**2a**, 0.4 mmol, 63.6 mg), 4Å molecular sieves (40 mg) and $Cu(hfacac)_2$ (0.04 mmol, 19.1 mg) in toluene (4.0 mL) at 120 °C (120 °C, heating mantle temperature). Purification by column chromatography on silica gel (petroleum ether/ethyl acetate = 5:1) yielded **3b** (71.9 mg, 72%) as a pale yellow oil.

^1H NMR (400 MHz, $CDCl_3$) δ 8.13 – 8.05 (m, 3H), 7.92 (d, *J* = 8.0 Hz, 1H), 7.52 (t, *J* = 7.8 Hz, 1H), 7.20 (t, *J* = 7.5 Hz, 1H), 7.15 (d, *J* = 7.1 Hz, 1H), 7.08 – 7.02 (m, 3H), 6.87 – 6.79 (m, 2H), 6.31 (s, 1H), 6.18 (d, *J* = 12.3 Hz, 1H), 3.97 (s, 1H), 2.11 (s, 3H), 1.25 (s, 9H); ^{13}C NMR (100 MHz, $CDCl_3$) δ 193.3, 191.5, 165.7 (d, *J* = 254.0 Hz), 151.2, 144.0, 139.1, 134.4, 132.6, 132.3 (d, *J* = 10.0 Hz), 132.1 (d, *J* = 3.0 Hz), 131.9, 130.4, 128.3, 124.7, 124.5, 122.9, 122.4, 121.6, 121.4, 120.9, 115.7 (d, *J* = 22.0 Hz), 83.3, 72.4, 71.0, 27.7, 17.6; IR (neat): 3400(br), 2920, 2849, 1706, 1670, 1597, 1505, 1458, 1369, 1302, 1236, 1117, 1012, 932, 851; HRMS (ESI) m/z: $[M + H]^+$ calcd for $C_{30}H_{28}FN_2O_4$ 499.2028, found 499.2022.

***N*-(2-(1-((4-bromophenyl)sulfonyl)-4-phenyl-1*H*-pyrrole-3-carbonyl)phenyl)benzamide (3c)**

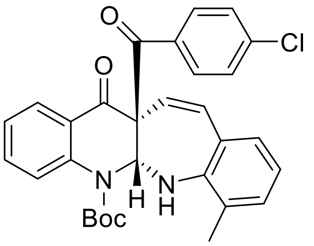

3c

The reaction was conducted with *tert*-butyl (2-(3-(4-chlorophenyl)propioloyl)phenyl)carbamate (**1c**, 0.2 mmol, 71.2 mg), 8-methylquinoline *N*-oxide (**2a**, 0.4 mmol, 63.6 mg), 4Å molecular sieves (40 mg) and Cu(hfacac)$_2$ (0.04 mmol, 19.1 mg) in toluene (4.0 mL) at 120 ^{o}C (120 °C, heating mantle temperature). Purification by column chromatography on silica gel (petroleum ether/ethyl acetate = 5:1) yielded **3c** (96.8 mg, 94%) as a pale yellow oil.

^{1}H NMR (400 MHz, CDCl$_3$) δ 8.06 (d, *J* = 7.8 Hz, 1H), 7.96 – 7.91 (m, 3H), 7.52 (t, *J* = 7.6 Hz, 1H), 7.36 (d, *J* = 7.8 Hz, 2H), 7.21 (t, *J* = 7.5 Hz, 1H), 7.15 (d, *J* = 7.7 Hz, 1H), 7.03 (d, *J* = 7.3 Hz, 1H), 6.88 – 6.80 (m, 2H), 6.29 (s, 1H), 6.16 (d, *J* = 12.3 Hz, 1H), 3.96 (s, 1H), 2.12 (s, 3H), 1.24 (s, 9H); ^{13}C NMR (100 MHz, CDCl$_3$) δ 193.8, 191.4, 151.2, 144.0, 139.9, 139.2, 134.5, 134.1, 132.8, 132.0, 130.9, 130.4, 128.9, 128.3, 124.7, 124.5, 122.9, 122.2, 121.6, 121.3, 121.0, 83.4, 72.4, 71.1, 27.7, 17.6; IR (neat): 3401(br), 2921, 2850, 1705, 1670, 1598, 1471, 1458, 1369, 1242, 1145, 1117, 1012, 882, 766; HRMS (ESI) m/z: [M + H]$^+$ calcd for $C_{30}H_{28}ClN_2O_4$ 515.1732, found 515.1728.

***tert*-butyl 11a-(4-bromobenzoyl)-4-methyl-11-oxo-5,5a,11,11a-tetrahydro-6H-benzo[6,7]azepino[2,3-*b*]quinoline-6-carboxylate (3d)**

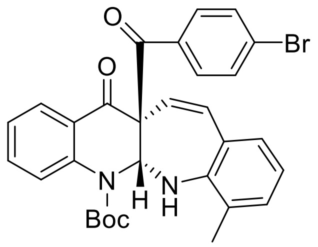

3d

The reaction was conducted with *tert*-butyl (2-(3-(4-bromophenyl)propioloyl)phenyl)carbamate (**1d**, 0.2 mmol, 80.0 mg), 8-methylquinoline *N*-oxide (**2a**, 0.4 mmol, 63.6 mg), 4Å molecular sieves (40 mg) and Cu(hfacac)$_2$ (0.04 mmol, 19.1 mg) in toluene (4.0 mL) at 120 °C (120 °C, heating mantle temperature). Purification by column chromatography on silica gel (petroleum ether/ethyl acetate = 5:1) yielded **3d** (69.3 mg, 62%) as a pale yellow oil.

^{1}H NMR (400 MHz, CDCl$_3$) δ 8.06 (d, *J* = 7.8 Hz, 1H), 7.93 (d, *J* = 8.5 Hz, 1H), 7.87 (d, *J* = 7.0 Hz, 2H), 7.54 – 7.50 (m, 3H), 7.21 (t, *J* = 7.5 Hz, 1H), 7.15 (d, *J* = 7.7 Hz, 1H), 7.03 (d, *J* = 7.3 Hz, 1H), 6.88 – 6.79 (m, 2H), 6.28 (s, 1H), 6.16 (d, *J* = 12.4 Hz, 1H), 3.96 (s, 1H), 2.12 (s, 3H), 1.25 (s, 9H); ^{13}C NMR (100 MHz, CDCl$_3$) δ 194.0, 191.3, 151.2, 144.0, 139.2, 134.6, 134.5, 132.8, 132.0, 131.9, 130.9, 130.4, 128.6, 128.3, 124.7, 124.5, 122.9, 122.1, 121.5, 121.3, 121.0, 83.4, 72.4, 71.1, 27.7, 17.6; IR (neat): 3401(br), 2924, 1705, 1671, 1598, 1583, 1471, 1458, 1394, 1303, 1145, 1117, 1071, 798, 706; HRMS (ESI) m/z: [M + H]$^+$ calcd for $C_{30}H_{28}BrN_2O_4$ 559.1227, found 559.1224.

***tert*-butyl 4-methyl-11-oxo-11a-(4-(trifluoromethyl)benzoyl)-5,5a,11,11a-tetrahydro-6*H*-benzo[6,7]azepino[2,3-*b*]quinoline-6-carboxylate (3e)**

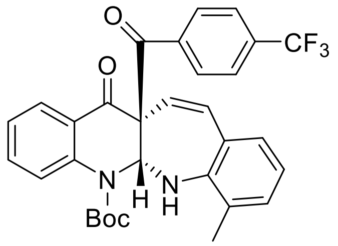

3e

The reaction was conducted with *tert*-butyl (2-(3-(4-(trifluoromethyl)phenyl)propioloyl)phenyl)carbamate (**1e**, 0.2 mmol, 77.8 mg), 8-methylquinoline *N*-oxide (**2a**, 0.4 mmol, 63.6 mg), 4Å molecular sieves (40 mg) and Cu(hfacac)$_2$ (0.04 mmol, 19.1 mg) in toluene (4.0 mL) at 120 ^{o}C (120 °C, heating mantle temperature). Purification by column chromatography on silica gel (petroleum ether/ethyl acetate = 5:1) yielded **3e** (90.0 mg, 82%) as a pale yellow oil.

^{1}H NMR (400 MHz, CDCl$_3$) δ 8.12 – 8.04 (m, 3H), 7.96 – 7.93 (m, 1H), 7.68 – 7.65 (m, 2H), 7.54 (t, *J* = 9.4 Hz, 1H), 7.23 – 7.16 (m, 2H), 7.07 – 7.01 (m, 1H), 6.86 (t, *J* = 14.0 Hz, 2H), 6.28 – 6.27 (m, 1H), 6.17 (d, *J* = 12.4 Hz, 1H), 3.94 (s, 1H), 2.12 (s, 3H), 1.21 (s, 9H); ^{13}C NMR (100 MHz, CDCl$_3$) δ 194.3, 191.0, 151.2, 144.1, 139.2, 134.7, 133.2, 132.0, 130.5, 129.6, 128.5, 125.6 (q, *J* = 6.8 Hz), 124.7, 124.6, 122.9, 121.7, 121.4, 121.1, 83.5, 72.4, 71.2, 27.7, 17.6; IR (neat): 3391(br), 2920, 2849, 1705, 1646, 1600, 1470, 1417, 1370, 1324, 1258, 1130, 1041, 799, 764; HRMS (ESI) m/z: $[M + H]^+$ calcd for $C_{31}H_{28}F_3N_2O_4$ 549.1996, found 549.1989.

***tert*-butyl 11a-(4-cyanobenzoyl)-4-methyl-11-oxo-5,5a,11,11a-tetrahydro-6*H*-benzo[6,7]azepino[2,3-*b*]quinoline-6-carboxylate (3f)**

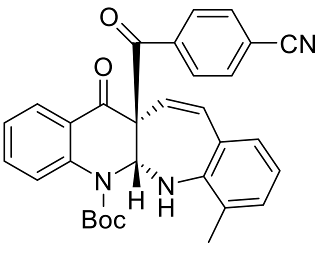

3f

The reaction was conducted with *tert*-butyl (2-(3-(4-cyanophenyl)propioloyl)phenyl)carbamate (**1f**, 0.2 mmol, 69.2 mg), 8-methylquinoline *N*-oxide (**2a**, 0.4 mmol, 63.6 mg), 4Å molecular sieves (40 mg) and Cu(hfacac)$_2$ (0.04 mmol, 19.1 mg) in toluene (4.0 mL) at 120 ^{o}C (120 °C, heating

mantle temperature). Purification by column chromatography on silica gel (petroleum ether/ethyl acetate = 5:1) yielded **3f** (73.9 mg, 73%) as a pale yellow oil.

^{1}H NMR (400 MHz, $CDCl_3$) δ 8.08 (d, *J* = 4.9 Hz, 3H), 7.91 (d, *J* = 7.2 Hz, 1H), 7.70 (d, *J* = 5.5 Hz, 2H), 7.59 – 7.50 (m, 1H), 7.23 – 7.15 (m, 2H), 7.04 (d, *J* = 6.0 Hz, 1H), 6.87 – 6.82 (m, 2H), 6.27 (s, 1H), 6.15 (d, *J* = 11.1 Hz, 1H), 3.98 (s, 1H), 2.12 (s, 3H), 1.27 (s, 9H); ^{13}C NMR (100 MHz, $CDCl_3$) δ 194.0, 190.9, 151.2, 144.0, 139.2, 134.8, 133.3, 132.3, 132.0, 130.6, 129.8, 128.4, 124.8, 124.7, 122.8, 121.8, 121.4, 121.3, 121.1, 116.5, 83.6, 72.2, 71.3, 27.8, 17.6; IR (neat): 3446(br), 2924, 2853, 2231, 1704, 1697, 1656, 1471, 1395, 1291, 1145, 1088, 1041, 850, 708; HRMS (ESI) m/z: [M + H]$^+$ calcd for $C_{31}H_{28}N_3O_4$ 506.2074, found 506.2072.

***tert*-butyl 11a-([1,1'-biphenyl]-4-carbonyl)-4-methyl-11-oxo-5,5a,11,11a-tetrahydro-6*H*-benzo[6,7]azepino[2,3-*b*]quinoline-6-carboxylate (3g)**

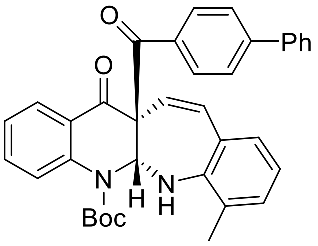

3g

The reaction was conducted with *tert*-butyl (2-(3-([1,1'-biphenyl]-4-yl)propioloyl)phenyl)carbamate (**1g**, 0.2 mmol, 79.4 mg), 8-methylquinoline *N*-oxide (**2a**, 0.4 mmol, 63.6 mg), 4Å molecular sieves (40 mg) and Cu(hfacac)$_2$ (0.04 mmol, 19.1 mg) in toluene (4.0 mL) at 120 ^{o}C (120 °C, heating mantle temperature). Purification by column chromatography on silica gel (petroleum ether/ethyl acetate = 5:1) yielded **3g** (102.5 mg, 92%) as a pale yellow oil.

^{1}H NMR (400 MHz, $CDCl_3$) δ 8.09 (d, *J* = 7.9 Hz, 3H), 7.97 (d, *J* = 7.8 Hz, 1H), 7.61 – 7.39 (m, 8H), 7.23 – 7.17 (m, 2H), 7.03 (d, *J* = 7.0 Hz, 1H), 6.88 – 6.83 (m, 2H), 6.38 (s, 1H), 6.24 (d, *J* = 12.3 Hz, 1H), 3.99 (s, 1H), 2.13 (s, 3H), 1.19 (s, 9H); ^{13}C NMR (100 MHz, $CDCl_3$) δ 194.4, 191.6, 151.2, 146.0, 144.0, 139.6, 139.2, 134.5,

134.4, 132.6, 132.0, 130.3, 130.0, 129.0, 128.3(4), 128.3(2), 127.1(8), 127.1(6), 124.6, 124.4, 123.1, 122.6, 121.4, 121.3, 120.9, 83.2, 72.5, 71.2, 27.7, 17.7; IR (neat): 3401(br), 2925, 2853, 1867, 1725, 1705, 1668, 1557, 1470, 1458, 1306, 1194, 1041, 932, 874; HRMS (ESI) m/z: $[M + H]^+$ calcd for $C_{36}H_{33}N_2O_4$ 557.2435, found 557.2430.

***tert*-butyl 4-methyl-11a-(4-methylbenzoyl)-11-oxo-5,5a,11,11a-tetrahydro-6*H*-benzo[6,7]azepino[2,3-*b*]quinoline-6-carboxylate (3h)**

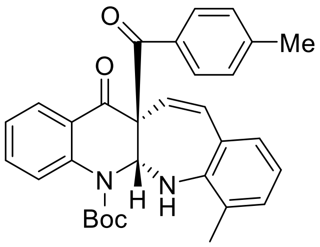

3h

The reaction was conducted with *tert*-butyl (2-(3-(*p*-tolyl)propioloyl)phenyl)carbamate (**1h**, 0.2 mmol, 64.2 mg), 8-methylquinoline *N*-oxide (**2a**, 0.4 mmol, 67.0 mg), 4Å molecular sieves (40 mg) and $Cu(hfacac)_2$ (0.04 mmol, 19.1 mg) in toluene (4.0 mL) at 120 °C (120 °C, heating mantle temperature). Purification by column chromatography on silica gel (petroleum ether/ethyl acetate = 5:1) yielded **3h** (92.1 mg, 93%) as a pale yellow oil.

^{1}H NMR (400 MHz, $CDCl_3$) δ 8.06 (d, *J* = 7.7 Hz, 1H), 7.95 – 7.89 (m, 3H), 7.51 (t, *J* = 7.8 Hz, 1H), 7.22 – 7.14 (m, 4H), 7.02 (d, *J* = 7.0 Hz, 1H), 6.87 – 6.78 (m, 2H), 6.33 (s, 1H), 6.20 (d, *J* = 12.3 Hz, 1H), 3.96 (s, 1H), 2.35 (s, 3H), 2.12 (s, 3H), 1.20 (s, 9H); ^{13}C NMR (100 MHz, $CDCl_3$) δ 194.5, 191.8, 151.2, 144.2, 144.0, 139.2, 134.3, 133.3, 132.3, 131.9, 130.2, 129.6, 129.2, 128.3, 124.6, 124.4, 123.1, 122.8, 121.4, 120.8, 83.1, 72.5, 71.1, 27.6, 21.6, 17.7; IR (neat): 3401(br), 2974, 2923, 2852, 1725, 1704, 1667, 1599, 1458, 1306, 1256, 1116, 1041, 931, 884; HRMS (ESI) m/z: $[M + H]^+$ calcd for $C_{31}H_{31}N_2O_4$ 495.2278, found 495.2274.

***tert*-butyl 11a-(4-ethylbenzoyl)-4-methyl-11-oxo-5,5a,11,11a-tetrahydro-6*H*-benzo[6,7]azepino[2,3-*b*]quinoline-6-carboxylate (3i)**

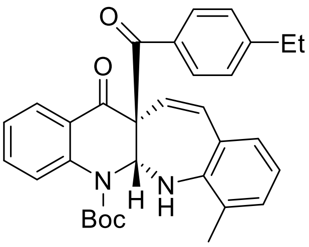

3i

The reaction was conducted with *tert*-butyl (2-(3-(4-ethylphenyl)propioloyl)phenyl)carbamate (**1i**, 0.2 mmol, 69.8 mg), 8-methylquinoline *N*-oxide (**2a**, 0.4 mmol, 63.6 mg), 4Å molecular sieves (40 mg) and Cu(hfacac)$_2$ (0.04 mmol, 19.1 mg) in toluene (4.0 mL) at 120 ^{o}C (120 °C, heating mantle temperature). Purification by column chromatography on silica gel (petroleum ether/ethyl acetate = 5:1) yielded **3i** (91.6 mg, 90%) as a pale yellow oil.

^{1}H NMR (400 MHz, CDCl$_3$) δ 8.08 (d, *J* = 7.8 Hz, 1H), 7.95 – 7.90 (m, 3H), 7.51 (t, *J* = 7.9 Hz, 1H), 7.21 – 7.15 (m, 4H), 7.02 (d, *J* = 7.3 Hz, 1H), 6.87 – 6.79 (m, 2H), 6.35 (s, 1H), 6.20 (d, *J* = 12.3 Hz, 1H), 3.97 (s, 1H), 2.65 (q, *J* = 7.5 Hz, 2H), 2.11 (s, 3H), 1.22 – 1.18 (m, 12H); ^{13}C NMR (100 MHz, CDCl$_3$) δ 194.5, 191.7, 151.1, 150.4, 144.0, 139.2, 134.3, 133.4, 132.3, 131.9, 130.2, 129.7, 128.3, 128.1, 124.6, 124.3, 123.1, 122.8, 121.4, 120.8, 83.0, 72.5, 71.1, 28.9, 27.7, 17.6, 15.1; IR (neat): 3401(br), 2971, 2928, 1726, 1705, 1668, 1600, 1470, 1458, 1411, 1156, 1145, 1015, 884, 798; HRMS (ESI) m/z: [M + H]$^+$ calcd for $C_{32}H_{33}N_2O_4$ 509.2435, found 509.2432.

***tert*-butyl 11a-(4-(*tert*-butyl)benzoyl)-4-methyl-11-oxo-5,5a,11,11a-tetrahydro-6*H*-benzo[6,7]azepino[2,3-*b*]quinoline-6-carboxylate (3j)**

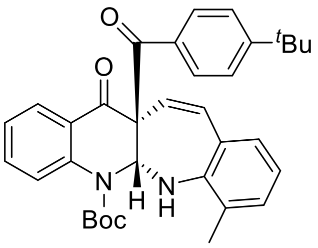

3j

The reaction was conducted with *tert*-butyl (2-(3-(4-(*tert*-butyl)phenyl)propioloyl)phenyl)carbamate (**1j**, 0.2 mmol, 75.4 mg), 8-methylquinoline *N*-oxide (**2a**, 0.4 mmol, 63.6 mg), 4Å molecular sieves (40 mg) and Cu(hfacac)$_2$ (0.04 mmol, 19.1 mg) in toluene (4.0 mL) at 120 °C (120 °C, heating mantle temperature). Purification by column chromatography on silica gel (petroleum ether/ethyl acetate = 5:1) yielded **3j** (88.1 mg, 82%) as a pale yellow oil.

^{1}H NMR (400 MHz, $CDCl_3$) δ 8.10 (d, *J* = 7.6 Hz, 1H), 7.99 – 7.94 (m, 3H), 7.51 (t, *J* = 7.7 Hz, 1H), 7.40 (d, *J* = 8.5 Hz, 2H), 7.21 – 7.16 (m, 2H), 7.02 (s, 1H), 6.86 – 6.79 (m, 2H), 6.38 (s, 1H), 6.20 (d, *J* = 12.3 Hz, 1H), 3.97 (s, 1H), 2.11 (s, 3H), 1.29 (s, 9H), 1.15 (s, 9H); ^{13}C NMR (100 MHz, $CDCl_3$) δ 194.4, 191.6, 157.2, 151.1, 144.0, 139.1, 134.3, 133.1, 132.3, 131.9, 130.2, 129.5, 128.3, 125.6, 124.6, 124.3, 123.1, 122.8, 121.3, 120.8, 83.0, 72.4, 71.1, 35.1, 30.9, 27.6, 17.6; IR (neat): 3401(br), 2965, 2927, 1727, 1705, 1668, 1600, 1470, 1458, 1394, 1256, 1156, 1041, 933, 798; HRMS (ESI) m/z: $[M + H]^+$ calcd for $C_{34}H_{37}N_2O_4$ 537.2748, found 537.2742.

***tert*-butyl 11a-(4-methoxybenzoyl)-4-methyl-11-oxo-5,5a,11,11a-tetrahydro-6*H*-benzo[6,7]azepino[2,3-*b*]quinoline-6-carboxylate (3k)**

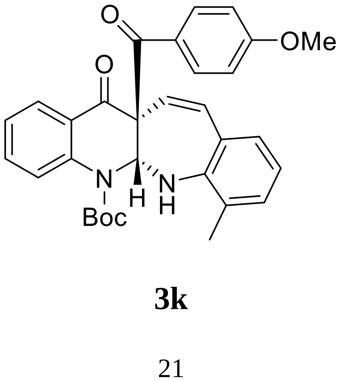

3k

The reaction was conducted with *tert*-butyl (2-(3-(4-methoxyphenyl)propioloyl)phenyl)carbamate (**1k**, 0.2 mmol, 70.2 mg), 8-methylquinoline *N*-oxide (**2a**, 0.4 mmol, 63.6 mg), 4Å molecular sieves (40 mg) and Cu(hfacac)$_2$ (0.04 mmol, 19.1 mg) in toluene (4.0 mL) at 120 °C (120 °C, heating mantle temperature). Purification by column chromatography on silica gel (petroleum ether/ethyl acetate = 5:1) yielded **3k** (85.8 mg, 84%) as a pale yellow oil.

^{1}H NMR (400 MHz, CDCl$_3$) δ 8.07 – 8.02 (m, 3H), 7.92 (d, *J* = 8.5 Hz, 1H), 7.51 (t, *J* = 7.8 Hz, 1H), 7.20 – 7.14 (m, 2H), 7.02 (d, *J* = 7.2 Hz, 1H), 6.87 – 6.77 (m, 4H), 6.35 (s, 1H), 6.21 (d, *J* = 12.3 Hz, 1H), 3.97 (s, 1H), 3.83 (s, 3H), 2.12 (s, 3H), 1.23 (s, 9H); ^{13}C NMR (100 MHz, CDCl$_3$) δ 193.1, 192.0, 163.6, 151.2, 144.0, 139.2, 134.2, 132.1, 132.0, 131.9, 130.2, 128.4, 128.2, 124.6, 124.4, 123.1, 123.0, 121.6, 120.8, 113.7, 83.1, 72.4, 70.9, 55.5, 27.7, 17.7; IR (neat): 3400(br), 2921, 2850, 1704, 1660, 1598, 1508, 1470, 1458, 1417, 1255, 1170, 1021, 884, 765; HRMS (ESI) m/z: [M + H]$^+$ calcd for $C_{31}H_{31}N_2O_5$ 511.2227, found 511.2224.

_N_-(2-(4-(thiophen-3-yl)-1-tosyl-1_H_-pyrrole-3-carbonyl)phenyl)benzamide (3l)

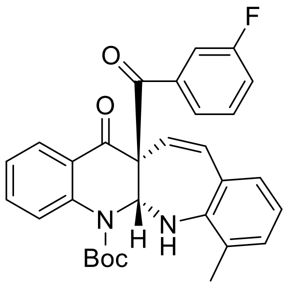

3l

The reaction was conducted with *tert*-butyl (2-(3-(3-fluorophenyl)propioloyl)phenyl)carbamate (**1l**, 0.2 mmol, 67.8 mg), 8-methylquinoline *N*-oxide (**2a**, 0.4 mmol, 63.6 mg), 4Å molecular sieves (40 mg) and Cu(hfacac)$_2$ (0.04 mmol, 19.1 mg) in toluene (4.0 mL) at 120 °C (120 °C, heating mantle temperature). Purification by column chromatography on silica gel (petroleum ether/ethyl acetate = 5:1) yielded **3l** (74.9 mg, 75%) as a pale yellow oil.

^{1}H NMR (400 MHz, $CDCl_3$) δ 8.07 (d, *J* = 7.8 Hz, 1H), 7.93 (d, *J* = 8.4 Hz, 1H), 7.84 (d, *J* = 7.2 Hz, 1H), 7.65 (d, *J* = 9.5 Hz, 1H), 7.53 (t, *J* = 7.8 Hz, 1H), 7.40 – 7.36 (m, 1H), 7.23 – 7.15 (m, 3H), 7.03 (d, *J* = 6.9 Hz, 1H), 6.88 – 6.81 (m, 2H), 6.31 (s, 1H), 6.17 (d, *J* = 12.3 Hz, 1H), 3.97 (s, 1H), 2.11 (s, 3H), 1.24 (s, 9H); ^{13}C NMR (100 MHz, $CDCl_3$) δ 193.8, 191.2, 162.5 (d, *J* = 247.0 Hz), 151.2, 144.0, 139.1, 137.8 (d, *J* = 7.0 Hz), 134.5, 132.9, 132.0, 130.4, 130.3 (d, *J* = 8.0 Hz), 128.4, 125.1 (d, *J* = 3.0 Hz), 124.6, 124.5, 122.9, 122.0, 121.5, 121.2, 121.0, 120.4 (d, *J* = 21.0 Hz), 116.2 (d, *J* = 23.0 Hz), 83.3, 72.3, 71.2, 27.7, 17.6; IR (neat): 3401(br), 2923, 2852, 1705, 1678, 1598, 1585, 1471, 1458, 1434, 1304, 1146, 832, 729; HRMS (ESI) m/z: $[M + H]^+$ calcd for $C_{30}H_{28}FN_2O_4$ 499.2028, found 499.2023.

tert-butyl 11a-(3-chlorobenzoyl)-4-methyl-11-oxo-5,5a,11,11a-tetrahydro-6_H_-benzo[6,7]azepino[2,3-_b_]quinoline-6-carboxylate (3m)

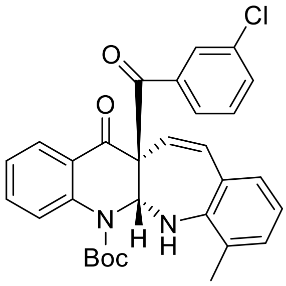

3m

The reaction was conducted with *tert*-butyl (2-(3-(3-chlorophenyl)propioloyl)phenyl)carbamate (**1m**, 0.2 mmol, 71.2 mg), 8-methylquinoline *N*-oxide (**2a**, 0.4 mmol, 63.6 mg), 4Å molecular sieves (40 mg) and $Cu(hfacac)_2$ (0.04 mmol, 19.1 mg) in toluene (4.0 mL) at 120 °C (120 °C, heating mantle temperature). Purification by column chromatography on silica gel (petroleum ether/ethyl acetate = 5:1) yielded **3m** (81.4 mg, 79%) as a pale yellow oil.

^{1}H NMR (400 MHz, $CDCl_3$) δ 8.07 (d, *J* = 7.8 Hz, 1H), 7.98 – 7.92 (m, 3H), 7.55 – 7.49 (m, 2H), 7.33 (t, *J* = 7.9 Hz, 1H), 7.24 – 7.16 (m, 2H), 7.03 (d, *J* = 7.3 Hz, 1H), 6.88 – 6.82 (m, 2H), 6.30 (s, 1H), 6.16 (d, *J* = 12.4 Hz, 1H), 3.97 (s, 1H), 2.12 (s, 3H), 1.24 (s, 9H); ^{13}C NMR (100 MHz, $CDCl_3$) δ 193.8, 191.1, 151.1, 144.0, 139.1, 137.4,

134.9, 134.6, 133.2, 133.0, 132.0, 130.4, 129.9, 129.4, 128.4, 127.4, 124.6, 124.5, 122.9, 121.9, 121.5, 121.2, 121.0, 83.4, 72.3, 71.2, 27.7, 17.6; IR (neat): 3401(br), 2975, 2921, 2851, 1867, 1828, 1790, 1770, 1728, 1672, 1598, 1506, 1041, 797, 674; HRMS (ESI) m/z: $[M + H]^+$ calcd for $C_{30}H_{28}ClN_2O_4$ 515.1732, found 515.1729.

***tert*-butyl 4-methyl-11a-(3-methylbenzoyl)-11-oxo-5,5a,11,11a-tetrahydro-6*H*-benzo[6,7]azepino[2,3-*b*]quinoline-6-carboxylate (3n)**

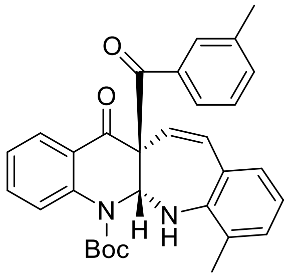

3n

The reaction was conducted with *tert*-butyl (2-(3-(m-tolyl)propioloyl)phenyl)carbamate (**1n**, 0.2 mmol, 67.0 mg), 8-methylquinoline *N*-oxide (**2a**, 0.4 mmol, 63.6 mg), 4Å molecular sieves (40 mg) and Cu(hfacac)$_2$ (0.04 mmol, 19.1 mg) in toluene (4.0 mL) at 120 °C (120 °C, heating mantle temperature). Purification by column chromatography on silica gel (petroleum ether/ethyl acetate = 5:1) yielded **3n** (90.1 mg, 91%) as a pale yellow oil.

^{1}H NMR (400 MHz, $CDCl_3$) δ 8.07 (d, *J* = 7.8 Hz, 1H), 7.96 (d, *J* = 8.5 Hz, 1H), 7.84 (d, *J* = 7.6 Hz, 1H), 7.75 (s, 1H), 7.51 (t, *J* = 7.8 Hz, 1H), 7.33 (d, *J* = 7.5 Hz, 1H), 7.22 – 7.15 (m, 2H), 7.02 (d, *J* = 7.2 Hz, 1H), 6.87 – 6.80 (m, 2H), 6.33 (s, 1H), 6.20 (d, *J* = 12.4 Hz, 1H), 3.96 (s, 1H), 2.31 (s, 3H), 2.12 (s, 3H), 1.19 (s, 9H); ^{13}C NMR (100 MHz, $CDCl_3$) δ 195.2, 191.6, 151.1, 144.1, 139.2, 138.5, 136.0, 134.4, 134.1, 132.4, 131.9, 130.2, 129.8, 128.4, 128.3, 126.5, 124.6, 124.4, 123.1, 122.7, 121.3, 120.9, 83.1, 72.5, 71.2, 27.6, 21.3, 17.7; IR (neat): 3420(br), 2922, 2851, 1704, 1668, 1598, 1470, 1458, 1417, 1304, 1259, 1145, 1118, 853, 706; HRMS (ESI) m/z: $[M + H]^+$ calcd for $C_{31}H_{31}N_2O_4$ 495.2278, found 495.2279.

***tert*-butyl 11a-(2-chlorobenzoyl)-4-methyl-11-oxo-5,5a,11,11a-tetrahydro-6*H*-benzo[6,7]azepino[2,3-*b*]quinoline-6-carboxylate (3o)**

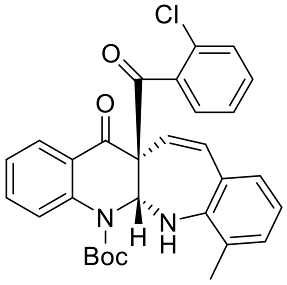

3o

The reaction was conducted with *tert*-butyl (2-(3-(2-chlorophenyl)propioloyl)phenyl)carbamate (**1o**, 0.2 mmol, 71.2 mg), 8-methylquinoline *N*-oxide (**2a**, 0.4 mmol, 63.6 mg), 4Å molecular sieves (40 mg) and Cu(hfacac)$_2$ (0.04 mmol, 19.1 mg) in toluene (4.0 mL) at 120 ^{o}C (120 °C, heating mantle temperature). Purification by column chromatography on silica gel (petroleum ether/ethyl acetate = 5:1) yielded **3o** (72.1 mg, 70%) as a pale yellow oil.

^{1}H NMR (400 MHz, CDCl$_3$) δ 8.12 (d, *J* = 6.1 Hz, 1H), 8.02 (d, *J* = 5.3 Hz, 1H), 7.56 (t, *J* = 8.3 Hz, 1H), 7.49 (d, *J* = 5.7 Hz, 1H), 7.38 – 7.29 (m, 2H), 7.22 – 7.19 (m, 2H), 7.11 (d, *J* = 5.6 Hz, 1H), 7.00 (d, *J* = 5.5 Hz, 1H), 6.83 (t, *J* = 7.4 Hz, 1H), 6.76 (d, *J* = 12.5 Hz, 1H), 6.17 (d, *J* = 12.4 Hz, 1H), 6.05 (s, 1H), 3.92 (s, 1H), 2.13 (s, 3H), 1.36 (s, 9H); ^{13}C NMR (100 MHz, CDCl$_3$) δ 196.5, 190.1, 151.5, 144.5, 140.2, 137.7, 135.0, 132.5, 131.6, 131.4, 131.3, 130.7, 130.1, 128.6, 127.5, 126.2, 124.6, 124.2, 123.4, 122.1, 121.1(4), 121.0(5), 120.5, 83.5, 72.9, 72.8, 27.9, 17.5; IR (neat): 3401(br), 2923, 2852, 1728, 1708, 1683, 1632, 1598, 1470, 1458, 1304, 1115, 1038, 799, 707; HRMS (ESI) m/z: [M + H]$^{+}$ calcd for $C_{30}H_{28}ClN_2O_4$ 515.1732, found 515.1729.

***tert*-butyl 11a-(2-bromobenzoyl)-4-methyl-11-oxo-5,5a,11,11a-tetrahydro-6*H*-benzo[6,7]azepino[2,3-*b*]quinoline-6-carboxylate (3p)**

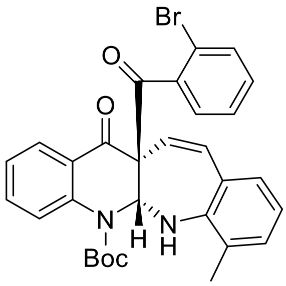

3p

The reaction was conducted with *tert*-butyl (2-(3-(2-bromophenyl)propioloyl)phenyl)carbamate (**1p**, 0.2 mmol, 80.0 mg), 8-methylquinoline *N*-oxide (**2a**, 0.4 mmol, 63.6 mg), 4Å molecular sieves (40 mg) and Cu(hfacac)$_2$ (0.04 mmol, 19.1 mg) in toluene (4.0 mL) at 120 °C (120 °C, heating mantle temperature). Purification by column chromatography on silica gel (petroleum ether/ethyl acetate = 5:1) yielded **3p** (55.8 mg, 50%) as a pale yellow oil.

^{1}H NMR (400 MHz, CDCl$_3$) δ 8.07 (d, *J* = 7.8 Hz, 1H), 8.00 – 7.94 (m, 3H), 7.53 – 7.50 (m, 2H), 7.40 – 7.36 (m, 2H), 7.20 – 7.15 (m, 2H), 7.02 (d, *J* = 7.3 Hz, 1H), 6.87 – 6.80 (m, 1H), 6.34 (s, 1H), 6.20 (d, *J* = 12.3 Hz, 1H), 3.97 (s, 1H), 2.12 (s, 3H), 1.19 (s, 9H); ^{13}C NMR (100 MHz, CDCl$_3$) δ 195.0, 191.5, 151.1, 144.0, 139.2, 136.0, 134.4, 133.3, 132.5, 131.9, 130.2, 129.4, 128.6, 128.3, 124.6, 124.4, 123.0, 122.5, 121.4, 121.3, 120.9, 83.2, 72.4, 71.2, 27.7, 17.6; IR (neat): 3438(br), 2921, 2849, 1643, 1598, 1470, 1458, 1419, 1306, 1233, 1145, 1088, 1041, 848, 696; HRMS (ESI) m/z: [M + H]$^+$ calcd for $C_{30}H_{28}BrN_2O_4$ 559.1227, found 559.1224.

***tert*-butyl 4-methyl-11a-(2-methylbenzoyl)-11-oxo-5,5a,11,11a-tetrahydro-6*H*-benzo[6,7]azepino[2,3-*b*]quinoline-6-carboxylate (3q)**

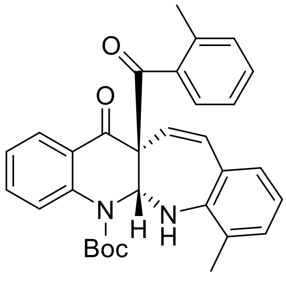

3q

The reaction was conducted with *tert*-butyl (2-(3-(o-tolyl)propioloyl)phenyl)carbamate (**1q**, 0.2 mmol, 67.0 mg), 8-methylquinoline *N*-oxide (**2a**, 0.4 mmol, 63.6 mg), 4Å molecular sieves (40 mg) and Cu(hfacac)$_2$ (0.04 mmol, 19.1 mg) in toluene (4.0 mL) at 120 ^{o}C (120 °C, heating mantle temperature). Purification by column chromatography on silica gel (petroleum ether/ethyl acetate = 5:1) yielded **3q** (76.2 mg, 77%) as a pale yellow oil.

^{1}H NMR (400 MHz, $CDCl_3$) δ 8.12 (d, *J* = 8.5 Hz, 1H), 8.06 (d, *J* = 6.2 Hz, 1H), 7.77 (d, *J* = 7.6 Hz, 1H), 7.53 (t, *J* = 7.8 Hz, 1H), 7.30 – 7.26 (m, 1H), 7.21 – 7.13 (m, 4H), 6.99 (d, *J* = 6.9 Hz, 1H), 6.85 – 6.79 (m, 2H), 6.16 (d, *J* = 12.3 Hz, 1H), 6.02 (s, 1H), 3.92 (s, 1H), 2.11 (s, 6H), 1.20 (s, 9H); ^{13}C NMR (100 MHz, $CDCl_3$) δ 198.3, 190.6, 151.3, 144.4, 139.6, 138.1, 137.5, 134.8, 132.6, 132.0, 131.8, 131.1, 130.1, 128.7, 126.9, 125.1, 124.5, 124.1, 123.3, 122.7, 120.9, 120.3, 120.1, 83.3, 73.3, 72.5, 27.7, 20.0, 17.6; IR (neat): 3407(br), 2975, 2926, 2853, 1726, 1707, 1681, 1598, 1470, 1231, 1097, 1040, 1010, 953, 798; HRMS (ESI) m/z: $[M + H]^+$ calcd for $C_{31}H_{31}N_2O_4$ 495.2278, found 495.2281.

tert-butyl 11a-(3,5-dimethylbenzoyl)-4-methyl-11-oxo-5,5a,11,11a-tetrahydro-6*H*-benzo[6,7]azepino[2,3-*b*]quinoline-6-carboxylate (3r)

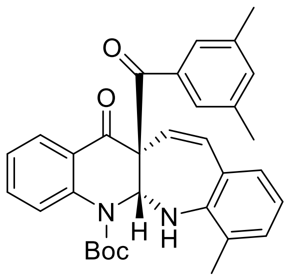

3r

The reaction was conducted with *tert*-butyl (2-(3-(3,5-dimethylphenyl)propioloyl)phenyl)carbamate (**1r**, 0.2 mmol, 69.8 mg), 8-methylquinoline *N*-oxide (**2a**, 0.4 mmol, 63.6 mg), 4Å molecular sieves (40 mg) and Cu(hfacac)$_2$ (0.04 mmol, 19.1 mg) in toluene (4.0 mL) at 120 ^{o}C (120 °C, heating mantle temperature). Purification by column chromatography on silica gel (petroleum

ether/ethyl acetate = 5:1) yielded **3r** (89.6 mg, 88%) as a yellow solid (mp 155-157 °C).

^{1}H NMR (400 MHz, $CDCl_3$) δ 8.07 – 7.97 (m, 2H), 7.59 – 7.49 (m, 3H), 7.20 – 7.15 (m, 3H), 7.02 (s, 1H), 6.88 – 6.80 (m, 2H), 6.32 (s, 1H), 6.21 (d, *J* = 12.3 Hz, 1H), 3.96 (s, 1H), 2.27 (s, 6H), 2.12 (s, 3H), 1.18 (s, 9H); ^{13}C NMR (100 MHz, $CDCl_3$) δ 195.3, 191.6, 151.1, 144.1, 139.2, 138.2, 136.2, 134.9, 134.4, 132.3, 131.7, 130.1, 128.3, 127.0, 124.6, 124.3, 123.2, 123.0, 121.3, 121.2, 120.9, 82.9, 72.7, 71.3, 27.6, 21.2, 17.6; IR (neat): 3407(br), 2974, 2923, 2853, 1727, 1704, 1669, 1599, 1558, 1470, 1458, 1197, 1099, 851, 706; HRMS (ESI) m/z: $[M + H]^+$ calcd for $C_{32}H_{33}N_2O_4$ 509.2435, found 509.2436.

***tert*-butyl 4-methyl-11-oxo-11a-(thiophene-3-carbonyl)-5,5a,11,11a-tetrahydro-6*H*-benzo[6,7]azepino[2,3-*b*]quinoline-6-carboxylate (3s)**

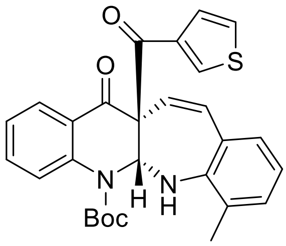

3s

The reaction was conducted with *tert*-butyl (2-(3-(thiophen-3-yl)propioloyl)phenyl)carbamate (**1s**, 0.2 mmol, 65.4 mg), 8-methylquinoline *N*-oxide (**2a**, 0.4 mmol, 63.6 mg), 4Å molecular sieves (40 mg) and $Cu(hfacac)_2$ (0.04 mmol, 19.1 mg) in toluene (4.0 mL) at 120 °C (120 °C, heating mantle temperature). Purification by column chromatography on silica gel (petroleum ether/ethyl acetate = 5:1) yielded **3s** (65.3 mg, 67%) as a pale yellow oil.

^{1}H NMR (400 MHz, $CDCl_3$) δ 8.33 (s, 1H), 8.06 (d, *J* = 5.4 Hz, 1H), 7.96 (d, *J* = 8.4 Hz, 1H), 7.57 – 7.51 (m, 2H), 7.19 – 7.13 (m, 2H), 7.02 (d, *J* = 5.0 Hz, 1H), 6.85 – 6.77 (m, 2H), 6.31 (s, 1H), 6.22 (d, *J* = 12.4 Hz, 1H), 3.96 (s, 1H), 2.13 (s, 3H), 1.30 (s, 9H); ^{13}C NMR (100 MHz, $CDCl_3$) δ 192.0, 188.2, 151.4, 144.3, 139.5, 139.0,

134.6, 134.4, 132.6, 131.8, 130.2, 128.4, 128.2, 125.7, 124.7, 124.3, 123.1, 122.8, 121.7, 121.5, 120.9, 83.2, 72.7, 71.3, 27.8, 17.6; IR (neat): 3419(br), 2923, 2852, 1703, 1697, 1660, 1598, 1470, 1368, 1242, 1145, 1118, 1041, 911, 759; HRMS (ESI) m/z: $[M + H]^+$ calcd for $C_{28}H_{27}N_2O_4S$ 487.1686, found 487.1686.

***tert*-butyl 11a-cinnamoyl-4-methyl-11-oxo-5,5a,11,11a-tetrahydro-6*H*-benzo[6,7]azepino[2,3-*b*]quinoline-6-carboxylate (3t)**

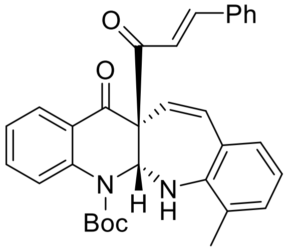

3t

The reaction was conducted with *tert*-butyl (*E*)-(2-(5-phenylpent-4-en-2-ynoyl)phenyl)carbamate (**1t**, 0.2 mmol, 69.4 mg), 8-methylquinoline *N*-oxide (**2a**, 0.4 mmol, 63.6 mg), 4Å molecular sieves (40 mg) and $Cu(hfacac)_2$ (0.04 mmol, 19.1 mg) in toluene (4.0 mL) at 120 °C (120 °C, heating mantle temperature). Purification by column chromatography on silica gel (petroleum ether/ethyl acetate = 5:1) yielded **3t** (69.0 mg, 68%) as a pale yellow oil.

^{1}H NMR (400 MHz, $CDCl_3$) δ 8.06 (d, *J* = 7.8 Hz, 1H), 7.93 (d, *J* = 8.5 Hz, 1H), 7.69 (d, *J* = 15.6 Hz, 1H), 7.56 – 7.52 (m, 3H), 7.39 – 7.36 (m, 3H), 7.18 (t, *J* = 7.5 Hz, 1H), 7.12 – 7.06 (m, 2H), 7.01 (d, *J* = 7.2 Hz, 1H), 6.83 (t, *J* = 7.5 Hz, 1H), 6.75 (d, *J* = 12.4 Hz, 1H), 6.13 – 6.07 (m, 2H), 3.95 (s, 1H), 2.16 (s, 3H), 1.43 (s, 9H); ^{13}C NMR (100 MHz, $CDCl_3$) δ 191.8, 191.2, 151.9, 145.8, 144.6, 140.1, 134.9, 134.1, 132.7, 131.5, 131.0, 130.0, 128.9, 128.8, 128.2, 124.6, 124.1, 123.4, 122.0, 121.4, 121.1, 120.8, 120.3, 83.3, 72.4, 72.1, 28.0, 17.6; IR (neat): 3445(br), 2922, 2850, 1673, 1632, 1599, 1576, 1470, 1458, 1304, 1227, 1124, 1041, 951, 732; HRMS (ESI) m/z: $[M + H]^+$ calcd for $C_{32}H_{31}N_2O_4$ 507.2278, found 507.2279.

***tert*-butyl 4-methyl-11-oxo-11a-pentanoyl-5,5a,11,11a-tetrahydro-6*H*-benzo[6,7]azepino[2,3-*b*]quinoline-6-carboxylate (3u)**

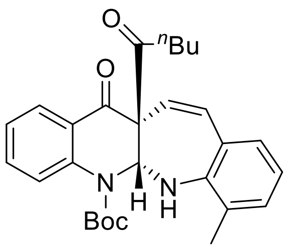

3u

The reaction was conducted with *tert*-butyl (2-(hept-2-ynoyl)phenyl)carbamate (**1u**, 0.2 mmol, 60.2 mg), 8-methylquinoline *N*-oxide (**2a**, 0.4 mmol, 63.6 mg), 4Å molecular sieves (40 mg) and Cu(hfacac)$_2$ (0.04 mmol, 19.1 mg) in toluene (4.0 mL) at 120 ^{o}C (120 °C, heating mantle temperature). Purification by column chromatography on silica gel (petroleum ether/ethyl acetate = 5:1) yielded **3u** (79.3 mg, 86%) as a pale yellow oil.

^{1}H NMR (400 MHz, CDCl$_3$) δ 8.04 (d, *J* = 7.8 Hz, 1H), 7.94 (d, *J* = 8.5 Hz, 1H), 7.55 (t, *J* = 7.9 Hz, 1H), 7.20 (t, *J* = 7.5 Hz, 1H), 7.08 (d, *J* = 7.7 Hz, 1H), 6.99 (d, *J* = 7.2 Hz, 1H), 6.81 (t, *J* = 7.5 Hz, 1H), 6.69 (d, *J* = 12.4 Hz, 1H), 6.06 – 6.00 (m, 2H), 3.87 (s, 1H), 2.73 – 2.65 (m, 1H), 2.49 – 2.41 (m, 1H), 2.14 (s, 3H), 1.49 – 1.42 (m, 11H), 1.26 – 1.19 (m, 2H), 0.83 (t, *J* = 7.3 Hz, 3H); ^{13}C NMR (100 MHz, CDCl$_3$) δ 203.6, 191.9, 151.9, 144.7, 140.1, 134.9, 132.3, 131.4, 129.9, 128.1, 124.6, 124.2, 123.4, 122.0, 121.6, 121.1, 120.9, 83.4, 73.2, 72.5, 38.6, 28.0, 25.5, 22.0, 17.5, 13.8; IR (neat): 3407(br), 2959, 2927, 2853, 1715, 1684, 1630, 1598, 1470, 1303, 1228, 1121, 1041, 797, 519; HRMS (ESI) m/z: [M + H]$^+$ calcd for $C_{28}H_{33}N_2O_4$ 461.2435, found 461.2434.

***tert*-butyl 11a-(cyclopropanecarbonyl)-4-methyl-11-oxo-5,5a,11,11a-tetrahydro-6*H*-benzo[6,7]azepino[2,3-*b*]quinoline-6-carboxylate (3v)**

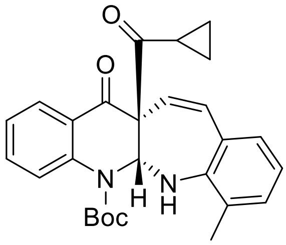

3v

The reaction was conducted with *tert*-butyl (2-(3-cyclopropylpropioloyl)phenyl)carbamate (**1v**, 0.2 mmol, 57.0 mg), 8-methylquinoline *N*-oxide (**2a**, 0.4 mmol, 63.6 mg), 4Å molecular sieves (40 mg) and Cu(hfacac)$_2$ (0.04 mmol, 19.1 mg) in toluene (4.0 mL) at 120 ^{o}C (120 °C, heating mantle temperature). Purification by column chromatography on silica gel (petroleum ether/ethyl acetate = 5:1) yielded **3v** (71.2 mg, 80%) as a pale yellow oil.

^{1}H NMR (400 MHz, $CDCl_3$) δ 8.05 (d, *J* = 7.8 Hz, 1H), 7.91 (d, *J* = 8.3 Hz, 1H), 7.54 (t, *J* = 6.9 Hz, 1H), 7.19 (t, *J* = 6.6 Hz, 1H), 7.08 (d, *J* = 7.5 Hz, 1H), 6.99 (d, *J* = 7.2 Hz, 1H), 6.81 (t, *J* = 7.5 Hz, 1H), 6.73 (d, *J* = 12.5 Hz, 1H), 6.18 (d, *J* = 12.4 Hz, 1H), 6.08 (s, 1H), 3.90 (s, 1H), 2.30 – 2.24 (m, 1H), 2.14 (s, 3H), 1.49 (s, 9H), 1.01 – 0.87 (m, 4H); ^{13}C NMR (100 MHz, $CDCl_3$) δ 203.5, 191.8, 151.9, 144.6, 140.1, 134.7, 132.3, 131.4, 129.9, 128.2, 124.6, 124.2, 123.5, 121.9, 121.8, 121.2, 120.9, 83.3, 73.1, 72.4, 28.1, 17.8, 17.5, 12.8, 12.7; IR (neat): 3419(br), 2978, 2920, 2849, 1704, 1697, 1681, 1657, 1645, 1599, 1417, 1228, 1041, 909, 797; HRMS (ESI) m/z: $[M + H]^+$ calcd for $C_{27}H_{29}N_2O_4$ 445.2122, found 445.2123.

***tert*-butyl 11a-benzoyl-9-chloro-4-methyl-11-oxo-5,5a,11,11a-tetrahydro-6*H*-benzo[6,7]azepino[2,3-*b*]quinoline-6-carboxylate (3w)**

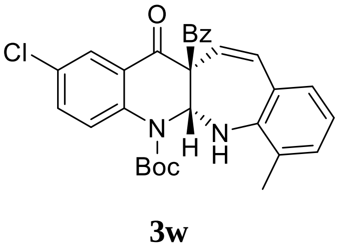

3w

The reaction was conducted with *tert*-butyl (4-chloro-2-(3-phenylpropioloyl)phenyl)carbamate (**1w**, 0.2 mmol, 71.1 mg), 8-methylquinoline *N*-oxide (**2a**, 0.4 mmol, 63.6 mg), 4Å molecular sieves (40 mg) and Cu(hfacac)$_2$ (0.04 mmol, 19.1 mg) in toluene (4.0 mL) at 120 ^{o}C (120 °C, heating mantle temperature). Purification by column chromatography on silica gel (petroleum ether/ethyl acetate = 5:1) yielded **3w** (77.3 mg, 75%) as a pale yellow oil.

^{1}H NMR (400 MHz, CDCl$_3$) ^{1}H NMR (400 MHz, CDCl$_3$) δ 8.03 – 7.93 (m, 4H), 7.54 – 7.39 (m, 4H), 7.17 (d, *J* = 7.0 Hz, 1H), 7.04 (d, *J* = 6.5 Hz, 1H), 6.88 – 6.81 (m, 2H), 6.34 (s, 1H), 6.17 (d, *J* = 11.9 Hz, 1H), 3.93 (s, 1H), 2.12 (s, 3H), 1.17 (s, 9H); ^{13}C NMR (100 MHz, CDCl$_3$) δ 194.9, 190.3, 150.9, 143.7, 137.6, 135.7, 134.1, 133.5, 132.7, 132.1, 130.4, 130.1, 129.4, 128.7, 127.7, 124.6, 123.1, 122.9, 122.6, 122.1, 121.0, 83.6, 72.2, 71.0, 27.6, 17.6; IR (neat): 3446(br), 2978, 2921, 2850, 1867, 1708, 1667, 1646, 1596, 1507, 1417, 1395, 1147, 848, 690; HRMS (ESI) m/z: [M + H]$^+$ calcd for $C_{30}H_{28}ClN_2O_4$ 515.1732, found 515.1735.

tert-butyl 11a-benzoyl-4,9-dimethyl-11-oxo-5,5a,11,11a-tetrahydro-6*H*-benzo[6,7]azepino[2,3-*b*]quinoline-6-carboxylate (3x)

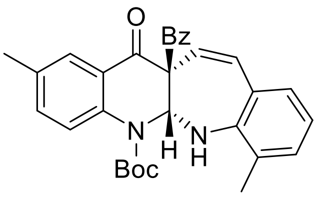

3x

The reaction was conducted with *tert*-butyl (4-methyl-2-(3-phenylpropioloyl)phenyl)carbamate (**1x**, 0.2 mmol, 67.0 mg), 8-methylquinoline *N*-oxide (**2a**, 0.4 mmol, 63.6 mg), 4Å molecular sieves (40 mg) and Cu(hfacac)$_2$ (0.04 mmol, 19.1 mg) in toluene (4.0 mL) at 120 ^{o}C (120 °C, heating mantle temperature). Purification by column chromatography on silica gel (petroleum ether/ethyl acetate = 5:1) yielded **3x** (76.2 mg, 77%) as a pale yellow oil.

^{1}H NMR (400 MHz, $CDCl_3$) δ 7.99 (d, *J* = 7.4 Hz, 2H), 7.88 – 7.83 (m, 2H), 7.51 (t, *J* = 7.4 Hz, 1H), 7.39 – 7.31 (m, 3H), 7.16 (d, *J* = 7.6 Hz, 1H), 7.02 (d, *J* = 7.3 Hz, 1H), 6.87 – 6.79 (m, 2H), 6.33 (s, 1H), 6.19 (d, *J* = 12.3 Hz, 1H), 3.96 (s, 1H), 2.35 (s, 3H), 2.11 (s, 3H), 1.18 (s, 9H); ^{13}C NMR (100 MHz, $CDCl_3$) δ 195.1, 191.7, 151.2, 144.1, 136.8, 136.0, 135.3, 134.2, 133.2, 132.4, 131.9, 130.2, 129.4, 128.6, 128.2, 124.6, 123.0, 122.7, 121.4, 121.1, 120.8, 82.9, 72.3, 71.2, 27.7, 20.5, 17.6; IR (neat): 3419(br), 2976, 2923, 2852, 1704, 1668, 1632, 1615, 1416, 1308, 1231, 1148, 1029, 970, 786; HRMS (ESI) m/z: $[M + H]^+$ calcd for $C_{31}H_{31}N_2O_4$ 495.2278, found 495.2281.

***tert*-butyl 11a-benzoyl-8-bromo-4-methyl-11-oxo-5,5a,11,11a-tetrahydro-6*H*-benzo[6,7]azepino[2,3-*b*]quinoline-6-carboxylate (3y)**

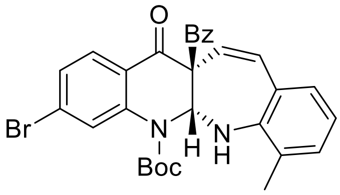

3y

The reaction was conducted with *tert*-butyl (5-bromo-2-(3-phenylpropioloyl)phenyl)carbamate (**1y**, 0.2 mmol, 80.0 mg), 8-methylquinoline *N*-oxide (**2a**, 0.4 mmol, 63.6 mg), 4Å molecular sieves (40 mg) and $Cu(hfacac)_2$ (0.04 mmol, 19.1 mg) in toluene (4.0 mL) at 120 ^{o}C (120 °C, heating mantle temperature). Purification by column chromatography on silica gel (petroleum ether/ethyl acetate = 5:1) yielded **3y** (87.2 mg, 78%) as a pale yellow oil.

^{1}H NMR (400 MHz, $CDCl_3$) δ 8.23 (s, 1H), 7.98 (d, *J* = 7.2 Hz, 2H), 7.93 (d, *J* = 8.4 Hz, 1H), 7.53 (t, *J* = 6.4 Hz, 1H), 7.41 – 7.34 (m, 3H), 7.17 (d, *J* = 6.5 Hz, 1H), 7.04 (d, *J* = 5.9 Hz, 1H), 6.89 – 6.81 (m, 2H), 6.32 (s, 1H), 6.17 (d, *J* = 12.3 Hz, 1H), 3.95 (s, 1H), 2.14 (s, 3H), 1.19 (s, 9H); ^{13}C NMR (100 MHz, $CDCl_3$) δ 194.8, 190.6, 150.8, 143.8, 139.9, 135.8, 133.5, 132.7, 132.0, 130.4, 129.6, 129.4, 129.3, 128.7, 127.7, 124.7, 124.2, 123.0, 122.0, 121.0, 120.0, 83.8, 72.4, 70.9, 27.6, 17.7; IR (neat):

3401(br), 2975, 2922, 2850, 1727, 1705, 1669, 1645, 1588, 1256, 1144, 1119, 1042, 937, 707; HRMS (ESI) m/z: $[M + H]^+$ calcd for $C_{30}H_{28}BrN_2O_4$ 559.1227, found 559.1232.

***tert*-butyl 11a-benzoyl-4,8-dimethyl-11-oxo-5,5a,11,11a-tetrahydro-6*H*-benzo[6,7]azepino[2,3-*b*]quinoline-6-carboxylate (3z)**

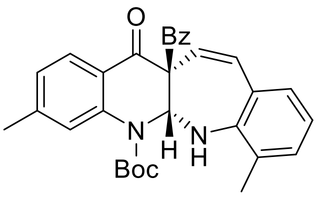

3z

The reaction was conducted with *tert*-butyl (5-methyl-2-(3-phenylpropioloyl)phenyl)carbamate (**1z**, 0.2 mmol, 67.0 mg), 8-methylquinoline *N*-oxide (**2a**, 0.4 mmol, 63.6 mg), 4Å molecular sieves (40 mg) and $Cu(hfacac)_2$ (0.04 mmol, 19.1 mg) in toluene (4.0 mL) at 120 °C (120 °C, heating mantle temperature). Purification by column chromatography on silica gel (petroleum ether/ethyl acetate = 4:1) yielded **3z** (81.2 mg, 82%) as a pale yellow oil.

^1H NMR (400 MHz, $CDCl_3$) δ 8.00 – 7.95 (m, 3H), 7.81 (s, 1H), 7.56 – 7.49 (m, 1H), 7.39 – 7.36 (m, 2H), 7.16 (d, J = 6.2 Hz, 1H), 7.02 (d, J = 7.1 Hz, 2H), 6.87 – 6.79 (m, 2H), 6.31 (s, 1H), 6.20 (d, J = 12.4 Hz, 1H), 3.96 (s, 1H), 2.40 (s, 3H), 2.13 (s, 3H), 1.19 (s, 9H); ^{13}C NMR (100 MHz, $CDCl_3$) δ 194.9, 191.3, 151.2, 145.7, 144.1, 139.2, 136.1, 133.2, 132.4, 131.9, 130.2, 129.3, 128.6, 128.3, 125.5, 124.6, 123.1, 122.7, 121.5, 120.8, 119.0, 83.0, 72.6, 71.1, 27.7, 22.3, 17.7; IR (neat): 3411(br), 2975, 2922, 2850, 1725, 1698, 1668, 1645, 1607, 1471, 1301, 1233, 1098, 955, 802; HRMS (ESI) m/z: $[M + H]^+$ calcd for $C_{31}H_{31}N_2O_4$ 495.2278, found 495.2281.

***tert*-butyl 11a-benzoyl-11-oxo-5,5a,11,11a-tetrahydro-6*H*-benzo[6,7]azepino[2,3-*b*]quinoline-6-carboxylate (3aa)**

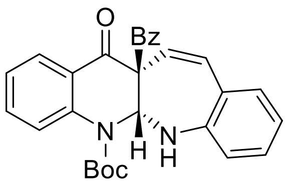

3aa

The reaction was conducted with *tert*-butyl (2-(3-phenylpropioloyl)phenyl)carbamate (**1a**, 0.2 mmol, 64.2 mg), quinoline *N*-oxide (**2b**, 0.4 mmol, 58.0 mg), 4Å molecular sieves (40 mg) and Cu(hfacac)$_2$ (0.04 mmol, 19.1 mg) in toluene (4.0 mL) at 120 °C (120 °C, heating mantle temperature). Purification by column chromatography on silica gel (petroleum ether/ethyl acetate = 4:1) yielded **3aa** (62.6 mg, 67%) as a pale yellow oil.

^{1}H NMR (400 MHz, CDCl$_3$) δ 8.07 (d, *J* = 6.2 Hz, 1H), 8.01 (d, *J* = 7.3 Hz, 2H), 7.83 (d, *J* = 8.4 Hz, 1H), 7.55 – 7.48 (m, 2H), 7.41 – 7.38 (m, 2H), 7.21 (t, *J* = 7.5 Hz, 1H), 7.09 (t, *J* = 7.6 Hz, 1H), 6.92 (t, *J* = 7.5 Hz, 1H), 6.80 (d, *J* = 12.4 Hz, 1H), 6.67 (d, *J* = 7.9 Hz, 1H), 6.44 (s, 1H), 6.17 (d, *J* = 12.3 Hz, 1H), 4.01 (s, 1H), 1.19 (s, 9H); ^{13}C NMR (100 MHz, CDCl$_3$) δ 195.1, 191.4, 151.1, 145.1, 139.0, 135.6, 134.0, 133.7, 133.4, 132.0, 129.5, 128.9, 128.6, 128.2, 124.6, 122.9, 122.6, 122.1, 121.7, 121.1, 118.5, 83.3, 71.3, 71.2, 27.7; IR (neat): 3434(br), 2978, 2919, 2849, 1698, 1666, 1599, 1476, 1459, 1369, 1259, 1145, 1041, 903, 749; HRMS (ESI) m/z: [M + H]$^+$ calcd for $C_{29}H_{27}N_2O_4$ 467.1965, found 467.1968.

***tert*-butyl 11a-benzoyl-4-ethyl-11-oxo-5,5a,11,11a-tetrahydro-6*H*-benzo[6,7]azepino[2,3-*b*]quinoline-6-carboxylate (3ab)**

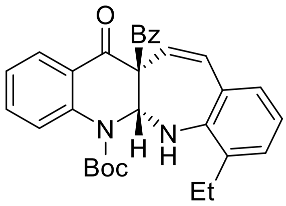

3ab

The reaction was conducted with *tert*-butyl (2-(3-phenylpropioloyl)phenyl)carbamate (**1a**, 0.2 mmol, 64.2 mg), 8-ethylquinoline *N*-oxide (**2c**, 0.4 mmol, 69.2 mg), 4Å molecular sieves (40 mg) and Cu(hfacac)$_2$ (0.04 mmol, 19.1 mg) in toluene (4.0 mL) at 120 °C (120 °C, heating mantle temperature). Purification by column chromatography on silica gel (petroleum ether/ethyl acetate = 5:1) yielded **3ab** (83.2 mg, 84%) as a pale yellow oil.

^{1}H NMR (400 MHz, CDCl$_3$) δ 8.09 (d, *J* = 7.7 Hz, 1H), 8.02 (d, *J* = 7.4 Hz, 2H), 7.93 (d, *J* = 8.4 Hz, 1H), 7.54 – 7.51 (m, 2H), 7.39 (d, *J* = 7.5 Hz, 2H), 7.23 – 7.16 (m, 2H), 7.03 (d, *J* = 7.2 Hz, 1H), 6.90 (t, *J* = 7.5 Hz, 1H), 6.82 (d, *J* = 12.4 Hz, 1H), 6.37 (s, 1H), 6.22 (d, *J* = 12.4 Hz, 1H), 4.04 (s, 1H), 2.50 – 2.38 (m, 2H), 1.17 – 1.12 (m, 12H); ^{13}C NMR (100 MHz, CDCl$_3$) δ 195.0, 191.6, 151.1, 143.5, 139.1, 135.9, 134.3, 133.4, 132.5, 131.9, 130.6, 129.5, 128.6, 128.5, 128.3, 124.5, 123.5, 122.7, 121.8, 121.5, 121.1, 83.2, 72.2, 71.3, 27.6, 24.3, 13.5; IR (neat): 3419(br), 2920, 2849, 1702, 1644, 1597, 1458, 1419, 1307, 1256, 1144, 1119, 1041, 919, 703; HRMS (ESI) m/z: [M + H]$^+$ calcd for $C_{31}H_{31}N_2O_4$ 495.2278, found 495.2276.

tert-butyl 11a-benzoyl-4-isopropyl-11-oxo-5,5a,11,11a-tetrahydro-6*H*-benzo[6,7]azepino[2,3-*b*]quinoline-6-carboxylate (3ac)

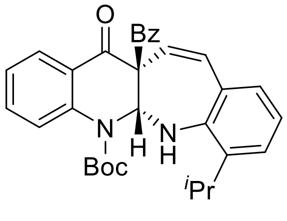

3ac

The reaction was conducted with *tert*-butyl (2-(3-phenylpropioloyl)phenyl)carbamate (**1a**, 0.2 mmol, 64.2 mg), 8-isopropylquinoline *N*-oxide (**2d**, 0.4 mmol, 74.8 mg), 4Å molecular sieves (40 mg) and Cu(hfacac)$_2$ (0.04 mmol, 19.1 mg) in toluene (4.0 mL) at 120 °C (120 °C, heating mantle temperature). Purification by column

chromatography on silica gel (petroleum ether/ethyl acetate = 10:1) yielded **3ac** (63.1 mg, 62%) as a yellow solid (mp 174-176 °C).

^{1}H NMR (400 MHz, $CDCl_3$) δ 8.10 (d, *J* = 7.8 Hz, 1H), 8.03 (d, *J* = 7.5 Hz, 2H), 7.94 (d, *J* = 8.4 Hz, 1H), 7.55 – 7.51 (m, 2H), 7.41 – 7.37 (m, 2H), 7.22 (t, *J* = 8.0 Hz, 1H), 7.16 (d, *J* = 7.6 Hz, 1H), 7.11 (d, *J* = 7.6 Hz, 1H), 6.94 (t, *J* = 7.6 Hz, 1H), 6.82 (d, *J* = 12.3 Hz, 1H), 6.38 (s, 1H), 6.24 (d, *J* = 12.4 Hz, 1H), 4.15 (s, 1H), 2.78 – 2.71 (m, 1H), 1.22 (d, *J* = 6.7 Hz, 3H), 1.16 (s, 9H), 1.12 (d, *J* = 6.6 Hz, 3H); ^{13}C NMR (100 MHz, $CDCl_3$) δ 195.0, 191.6, 151.0, 143.0, 139.1, 135.9, 135.1, 134.3, 133.4, 132.5, 131.5, 129.5, 128.6, 128.3, 125.3, 124.6, 123.8, 122.9, 121.9, 121.6, 121.3, 83.1, 72.4, 71.5, 27.7, 27.6, 22.9, 22.0; IR (neat): 3445(br), 2978, 2923, 2849, 1922, 1867, 1828, 1799, 1790, 1779, 1660, 1457, 1041, 953, 749; HRMS (ESI) m/z: $[M + H]^+$ calcd for $C_{32}H_{33}N_2O_4$ 509.2435, found 509.2437.

***tert*-butyl 11a-benzoyl-3-chloro-11-oxo-5,5a,11,11a-tetrahydro-6*H*-benzo[6,7]azepino[2,3-*b*]quinoline-6-carboxylate (3ad)**

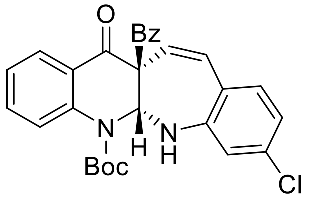

3ad

The reaction was conducted with *tert*-butyl (2-(3-phenylpropioloyl)phenyl)carbamate (**1a**, 0.2 mmol, 64.2 mg), 7-chloroquinoline *N*-oxide (**2e**, 0.4 mmol, 72.0 mg), 4Å molecular sieves (40 mg) and $Cu(hfacac)_2$ (0.04 mmol, 19.1 mg) in toluene (4.0 mL) at 120 °C (120 °C, heating mantle temperature). Purification by column chromatography on silica gel (petroleum ether/ethyl acetate = 10:1) yielded **3ad** (95.2 mg, 95%) as a pale yellow oil.

^{1}H NMR (400 MHz, $CDCl_3$) δ 8.05 (d, *J* = 7.8 Hz, 1H), 7.98 (d, *J* = 7.9 Hz, 2H), 7.79 (d, *J* = 8.5 Hz, 1H), 7.56 – 7.48 (m, 2H), 7.42 – 7.38 (m, 2H), 7.22 – 7.16 (m, 2H), 6.88 (d, *J* = 8.3 Hz, 1H), 6.73 (d, *J* = 12.3 Hz, 1H), 6.68 (s, 1H), 6.43 (s, 1H), 6.16 (d,

J = 12.3 Hz, 1H), 4.07 (s, 1H), 1.22 (s, 9H); ^{13}C NMR (100 MHz, $CDCl_3$) δ 194.7, 191.2, 151.1, 145.9, 138.8, 135.5, 134.7, 134.3, 134.2, 133.5, 131.0, 129.5, 128.7, 128.2, 124.7, 123.2, 122.2, 121.5, 121.1(4), 121.0(7), 118.1, 83.5, 71.1, 71.0, 27.7; IR (neat): 3445(br), 2064, 1867, 1828, 1790, 1703, 1687, 1639, 1558, 1458, 1368, 1236, 1041, 588, 469; HRMS (ESI) m/z: $[M + H]^+$ calcd for $C_{29}H_{26}ClN_2O_4$ 501.1576, found 501.1583.

tert-butyl 11a-benzoyl-11-oxo-3-(trifluoromethyl)-5,5a,11,11a-tetrahydro-6*H*-benzo[6,7]azepino[2,3-*b*]quinoline-6-carboxylate (3ae)

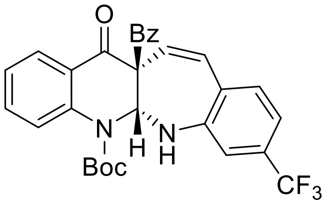

3ae

The reaction was conducted with *tert*-butyl (2-(3-phenylpropioloyl)phenyl)carbamate (**1a**, 0.2 mmol, 64.2 mg), 7-(trifluoromethyl)quinoline *N*-oxide (**2f**, 0.4 mmol, 63.6 mg), 4Å molecular sieves (40 mg) and $Cu(hfacac)_2$ (0.04 mmol, 19.1 mg) in toluene (4.0 mL) at 120 °C (120 °C, heating mantle temperature). Purification by column chromatography on silica gel (petroleum ether/ethyl acetate = 10:1) yielded **3ae** (85.2 mg, 87%) as a pale yellow oil.

^{1}H NMR (400 MHz, $CDCl_3$) δ 8.06 (d, J = 5.3 Hz, 1H), 7.98 (d, J = 6.7 Hz, 2H), 7.80 (d, J = 7.9 Hz, 1H), 7.57 – 7.50 (m, 2H), 7.43 – 7.36 (m, 3H), 7.23 – 7.14 (m, 2H), 6.94 (s, 1H), 6.82 (d, J = 12.4 Hz, 1H), 6.45 (s, 1H), 6.30 (d, J = 12.3 Hz, 1H), 4.23 (s, 1H), 1.23 (s, 9H); ^{13}C NMR (100 MHz, $CDCl_3$) δ 194.5, 191.1, 151.1, 145.0, 138.8, 135.4, 134.3, 134.1, 133.6, 130.9, 129.4, 128.7, 128.2, 125.6, 125.3, 124.8, 122.2, 121.4, 117.5 (q, J = 6.9 Hz), 115.3(4), 115.3(1), 83.6, 71.1(1), 71.0(7), 27.7; IR (neat): 3445(br), 2065, 1639, 1474, 1459, 1422, 1396, 1369, 1320, 1238, 1146, 1119, 1012,

508, 481; HRMS (ESI) m/z: $[M + H]^+$ calcd for $C_{30}H_{26}F_3N_2O_4$ 535.1839, found 535.1843.

tert-butyl 11a-benzoyl-3-methyl-11-oxo-5,5a,11,11a-tetrahydro-6*H*-benzo[6,7]azepino[2,3-*b*]quinoline-6-carboxylate (3af)

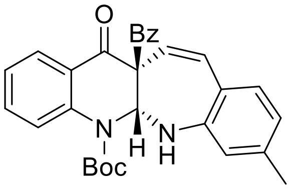

3af

The reaction was conducted with *tert*-butyl (2-(3-phenylpropioloyl)phenyl)carbamate (**1a**, 0.2 mmol, 64.2 mg), 7-methylquinoline *N*-oxide (**2g**, 0.4 mmol, 63.6 mg), 4Å molecular sieves (40 mg) and $Cu(hfacac)_2$ (0.04 mmol, 19.1 mg) in toluene (4.0 mL) at 120 °C (120 °C, heating mantle temperature). Purification by column chromatography on silica gel (petroleum ether/ethyl acetate = 10:1) yielded **3af** (89.5 mg, 93%) as a pale yellow oil.

^{1}H NMR (400 MHz, $CDCl_3$) δ 8.09 – 8.02 (m, 3H), 7.81 (d, *J* = 6.7 Hz, 1H), 7.54 – 7.49 (m, 2H), 7.40 – 7.37 (m, 2H), 7.19 – 7.13 (m, 2H), 6.73 (d, *J* = 7.6 Hz, 2H), 6.48 (d, *J* = 7.8 Hz, 2H), 6.12 (s, 1H), 3.98 (s, 1H), 2.22 (s, 3H), 1.19 (s, 9H); ^{13}C NMR (100 MHz, $CDCl_3$) δ 195.2, 191.3, 151.1, 144.9, 139.1, 138.9, 135.6, 133.9, 133.6, 133.4, 131.8, 129.6, 128.6, 128.1, 124.5, 122.2, 122.0, 121.9, 121.5, 120.0, 119.0, 83.2, 71.2, 71.1, 27.7, 21.0; IR (neat): 3445(br), 2978, 2925, 2854, 1703, 1698, 1668, 1646, 1615, 1599, 1579, 1558, 1146, 941, 814; HRMS (ESI) m/z: $[M + H]^+$ calcd for $C_{30}H_{29}N_2O_4$ 481.2122, found 481.2126.

tert-butyl 11a-benzoyl-2-fluoro-11-oxo-5,5a,11,11a-tetrahydro-6*H*-benzo[6,7]azepino[2,3-*b*]quinoline-6-carboxylate (3ag)

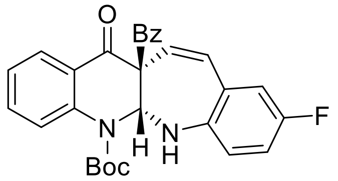

3ag

The reaction was conducted with *tert*-butyl (2-(3-phenylpropioloyl)phenyl)carbamate (**1a**, 0.2 mmol, 64.2 mg), 6-fluoroquinoline *N*-oxide (**2h**, 0.4 mmol, 65.2 mg), 4Å molecular sieves (40 mg) and Cu(hfacac)$_2$ (0.04 mmol, 19.1 mg) in toluene (4.0 mL) at 120 ^{o}C (120 °C, heating mantle temperature). Purification by column chromatography on silica gel (petroleum ether/ethyl acetate = 10:1) yielded **3ag** (95.1 mg, 98%) as a pale yellow oil.

^{1}H NMR (400 MHz, CDCl$_3$) δ 8.07 (d, *J* = 6.3 Hz, 1H), 7.98 (d, *J* = 7.5 Hz, 2H), 7.83 (d, *J* = 8.5 Hz, 1H), 7.54 – 7.49 (m, 2H), 7.42 – 7.38 (m, 2H), 7.21 (t, *J* = 7.5 Hz, 1H), 6.99 – 6.96 (m, 1H), 6.84 – 6.79 (m, 1H), 6.71 (d, *J* = 12.4 Hz, 1H), 6.64 – 6.61 (m, 1H), 6.38 (s, 1H), 6.26 (d, *J* = 12.3 Hz, 1H), 3.92 (s, 1H), 1.20 (s, 9H); ^{13}C NMR (100 MHz, CDCl$_3$) δ 194.8, 191.3, 157.6 (d, *J* = 238.0 Hz), 151.1, 141.5, 139.0, 135.6, 134.2, 133.5, 130.8, 129.4, 128.7, 128.2, 124.7 (d, *J* = 27.0 Hz), 124.0 (d, *J* = 7.0 Hz), 122.1, 121.5, 119.7 (d, *J* = 8.0 Hz), 118.8 (d, *J* = 22.0 Hz), 115.8 (d, *J* = 22.0 Hz), 83.4, 71.6, 71.2, 27.7; IR (neat): 3445(br), 2921, 2849, 2088, 1656, 1644, 1634, 1498, 1458, 1417, 1313, 1118, 1021, 959, 763; HRMS (ESI) m/z: [M + H]$^+$ calcd for $C_{29}H_{26}FN_2O_4$ 485.1871, found 485.1877.

tert-butyl 11a-benzoyl-2-chloro-11-oxo-5,5a,11,11a-tetrahydro-6*H*-benzo[6,7]azepino[2,3-*b*]quinoline-6-carboxylate (3ah)

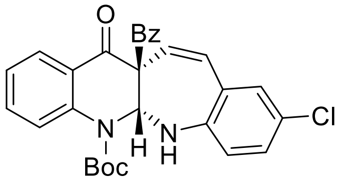

3ah

The reaction was conducted with *tert*-butyl (2-(3-phenylpropioloyl)phenyl)carbamate (**1a**, 0.2 mmol, 64.2 mg), 6-chloroquinoline *N*-oxide (**2i**, 0.4 mmol, 72.0 mg), 4Å molecular sieves (40 mg) and Cu(hfacac)$_2$ (0.04 mmol, 19.1 mg) in toluene (4.0 mL) at 120 ^{o}C (120 °C, heating mantle temperature). Purification by column chromatography on silica gel (petroleum ether/ethyl acetate = 10:1) yielded **3ah** (58.1 mg, 58%) as a pale yellow oil.

^{1}H NMR (400 MHz, $CDCl_3$) δ 8.06 (d, *J* = 7.7 Hz, 1H), 7.98 (d, *J* = 6.9 Hz, 2H), 7.82 (d, *J* = 10.0 Hz, 1H), 7.56 – 7.51 (m, 2H), 7.42 – 7.39 (m, 2H), 7.24 – 7.19 (m, 2H), 7.03 (d, *J* = 8.4 Hz, 1H), 6.70 (d, *J* = 12.4 Hz, 1H), 6.61 (d, *J* = 8.5 Hz, 1H), 6.40 (s, 1H), 6.23 (d, *J* = 9.9 Hz, 1H), 4.05 (s, 1H), 1.21 (s, 9H); ^{13}C NMR (100 MHz, $CDCl_3$) δ 194.7, 191.2, 151.1, 143.7, 138.9, 135.5, 134.2, 133.6, 132.6, 130.7, 129.4, 128.7, 128.6, 128.2, 125.9, 124.6(4), 124.5(9), 124.0, 122.1, 121.5, 119.8, 83.4, 71.3, 71.1, 27.7; IR (neat): 3434(br), 2979, 2924, 2851, 2366, 1693, 1666, 1636, 1597, 1507, 1487, 1313, 1041, 855, 762; HRMS (ESI) m/z: $[M + H]^+$ calcd for $C_{29}H_{26}ClN_2O_4$ 501.1576, found 501.1583.

tert-butyl 11a-benzoyl-2-bromo-11-oxo-5,5a,11,11a-tetrahydro-6*H*-benzo[6,7]azepino[2,3-*b*]quinoline-6-carboxylate (3ai)

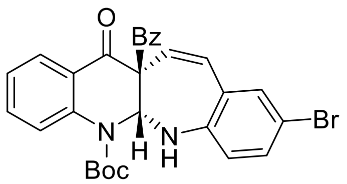

3ai

The reaction was conducted with *tert*-butyl (2-(3-phenylpropioloyl)phenyl)carbamate (**1a**, 0.2 mmol, 64.2 mg), 6-bromoquinoline *N*-oxide (**2j**, 0.4 mmol, 89.6 mg), 4Å molecular sieves (40 mg) and Cu(hfacac)$_2$ (0.04 mmol, 19.1 mg) in toluene (4.0 mL) at 120 ^{o}C (120 °C, heating mantle temperature). Purification by column chromatography on silica gel (petroleum ether/ethyl acetate = 10:1) yielded **3ai** (84.0 mg, 77%) as a pale yellow oil.

^{1}H NMR (400 MHz, $CDCl_3$) δ 8.07 (s, 1H), 7.98 (d, *J* = 7.5 Hz, 2H), 7.83 (d, *J* = 7.5 Hz, 1H), 7.56 – 7.50 (m, 2H), 7.42 – 7.38 (m, 3H), 7.20 – 7.14 (m, 2H), 6.68 (d, *J* = 8.0 Hz, 1H), 6.55 (d, *J* = 8.4 Hz, 1H), 6.41 (s, 1H), 6.23 (d, *J* = 8.4 Hz, 1H), 4.10 (s, 1H), 1.20 (s, 9H); ^{13}C NMR (100 MHz, $CDCl_3$) δ 194.7, 191.2, 151.1, 144.2, 138.9, 135.6, 135.5, 134.2, 133.6, 131.4, 130.6, 129.4, 128.7, 128.2, 124.7, 124.6, 124.4, 122.1, 121.4, 120.1, 113.1, 83.4, 71.2, 71.0, 27.7; IR (neat): 3445(br), 2924, 2851, 2065, 1657, 1644, 1634, 1558, 1507, 1459, 1422, 1311, 1117, 947, 755; HRMS (ESI) m/z: $[M + H]^+$ calcd for $C_{29}H_{26}BrN_2O_4$ 545.1070, found 545.1080.

6-(*tert*-butyl) 2-methyl 11a-benzoyl-11-oxo-5,5a,11,11a-tetrahydro-6*H*-benzo[6,7]azepino[2,3-*b*]quinoline-2,6-dicarboxylate (3aj)

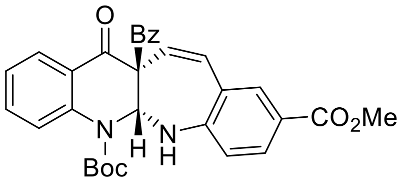

3aj

The reaction was conducted with *tert*-butyl (2-(3-phenylpropioloyl)phenyl)carbamate (**1a**, 0.2 mmol, 64.2 mg), 6-(methoxycarbonyl)quinoline *N*-oxide (**2k**, 0.4 mmol, 81.2 mg), 4Å molecular sieves (40 mg) and Cu(hfacac)$_2$ (0.04 mmol, 19.1 mg) in toluene (4.0 mL) at 120 °C (120 °C, heating mantle temperature). Purification by column chromatography on silica gel (petroleum ether/ethyl acetate = 10:1) yielded **3aj** (94.5 mg, 90%) as a pale yellow oil.

^{1}H NMR (400 MHz, $CDCl_3$) δ 8.05 – 7.99 (m, 4H), 7.84 (d, *J* = 7.0 Hz, 1H), 7.75 (s, 1H), 7.55 – 7.41 (m, 4H), 7.20 (s, 1H), 6.83 (d, *J* = 9.4 Hz, 1H), 6.67 (s, 1H), 6.49 (s, 1H), 6.21 (d, *J* = 10.0 Hz, 1H), 4.50 (s, 1H), 3.91 (s, 3H), 1.21 (s, 9H); ^{13}C NMR (100 MHz, $CDCl_3$) δ 194.4, 191.0, 166.4, 151.0, 148.9, 138.8, 135.8, 135.4, 134.3, 133.6, 131.5, 130.1, 129.5, 128.7, 128.2, 124.7, 123.4, 122.5, 122.1, 121.5, 121.4, 118.2, 83.5, 70.9, 70.8, 51.9, 27.7; IR (neat): 3445(br), 2980, 2927, 2852, 1698, 1668, 1645,

1600, 1506, 1477, 1370, 1234, 1011, 942, 708; HRMS (ESI) m/z: $[M + H]^+$ calcd for $C_{31}H_{29}N_2O_6$ 525.2020, found 525.2029.

***tert*-butyl 11a-benzoyl-2-nitro-11-oxo-5,5a,11,11a-tetrahydro-6*H*-benzo[6,7]azepino[2,3-*b*]quinoline-6-carboxylate (3ak)**

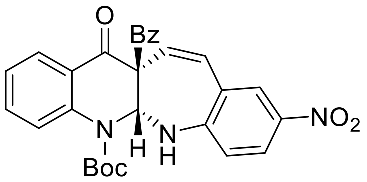

3ak

The reaction was conducted with *tert*-butyl (2-(3-phenylpropioloyl)phenyl)carbamate (**1a**, 0.2 mmol, 64.2 mg), 6-nitroquinoline *N*-oxide (**2l**, 0.4 mmol, 76.0 mg), 4Å molecular sieves (40 mg) and $Cu(hfacac)_2$ (0.04 mmol, 19.1 mg) in toluene (4.0 mL) at 120 °C (120 °C, heating mantle temperature). Purification by column chromatography on silica gel (petroleum ether/ethyl acetate = 10:1) yielded **3ak** (80.9 mg, 79%) as a pale yellow oil.

^{1}H NMR (400 MHz, $CDCl_3$) ^{1}H NMR (400 MHz, $CDCl_3$) δ 8.18 (s, 1H), 8.04 (d, *J* = 6.2 Hz, 1H), 7.98 (d, *J* = 7.0 Hz, 2H), 7.92 (d, *J* = 8.3 Hz, 1H), 7.82 (d, *J* = 7.5 Hz, 1H), 7.57 – 7.43 (m, 4H), 7.21 (s, 1H), 6.83 (d, *J* = 11.6 Hz, 1H), 6.68 (d, *J* = 8.1 Hz, 1H), 6.52 (s, 1H), 6.29 (d, *J* = 11.9 Hz, 1H), 4.74 (s, 1H), 1.25 (s, 9H); ^{13}C NMR (100 MHz, $CDCl_3$) δ 193.8, 190.6, 151.0, 150.0, 141.1, 138.6, 135.1, 134.5, 133.8, 130.6, 129.7, 129.4, 128.8, 128.3, 125.1, 124.9, 124.2, 122.2, 121.2(2), 121.1(5), 118.3, 84.0, 70.7, 70.5, 27.7; IR (neat): 3445(br), 2925, 2851, 2107, 1073, 1657, 1634, 1614, 1558, 1532, 1476, 1258, 1041, 747, 489; HRMS (ESI) m/z: $[M + H]^+$ calcd for $C_{29}H_{26}N_3O_6$ 512.1816, found 512.1826.

***tert*-butyl 11a-benzoyl-2-methyl-11-oxo-5,5a,11,11a-tetrahydro-6*H*-benzo[6,7]azepino[2,3-*b*]quinoline-6-carboxylate (3al)**

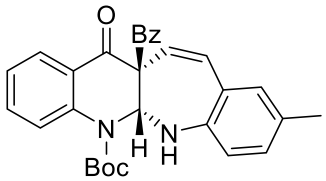

3al

The reaction was conducted with *tert*-butyl (2-(3-phenylpropioloyl)phenyl)carbamate (**1a**, 0.2 mmol, 64.2 mg), 6-methylquinoline *N*-oxide (**2m**, 0.4 mmol, 63.6 mg), 4Å molecular sieves (40 mg) and Cu(hfacac)$_2$ (0.04 mmol, 19.1 mg) in toluene (4.0 mL) at 120 ^{o}C (120 °C, heating mantle temperature). Purification by column chromatography on silica gel (petroleum ether/ethyl acetate = 4:1) yielded **3al** (92.4 mg, 96%) as a pale yellow oil.

^{1}H NMR (400 MHz, CDCl$_3$) δ 8.07 – 8.00 (m, 3H), 7.81 (d, *J* = 4.5 Hz, 1H), 7.53 – 7.50 (m, 2H), 7.40 – 7.38 (m, 2H), 7.22 – 7.19 (m, 1H), 7.06 (s, 1H), 6.89 (d, *J* = 5.4 Hz, 1H), 6.74 (d, *J* = 8.9 Hz, 1H), 6.57 (d, *J* = 3.4 Hz, 1H), 6.41 (s, 1H), 6.15 (d, *J* = 7.7 Hz, 1H), 3.92 (s, 1H), 2.28 (s, 3H), 1.18 (s, 9H); ^{13}C NMR (100 MHz, CDCl$_3$) δ 195.3, 191.5, 151.1, 142.8, 139.0, 135.7, 134.0, 133.8, 133.4, 132.0, 130.3, 129.7, 129.6, 128.6, 128.1, 124.5, 122.9, 122.6, 122.2, 121.8, 118.6, 83.2, 71.4, 71.2, 27.7, 20.3; IR (neat): 3445(br), 2923, 2852, 2065, 1697, 1660, 1643, 1600, 1505, 1476, 1313, 1147, 1010, 947, 764; HRMS (ESI) m/z: [M + H]$^+$ calcd for $C_{30}H_{29}N_2O_4$ 481.2122, found 481.2132.

***tert*-butyl 11a-benzoyl-2-butyl-11-oxo-5,5a,11,11a-tetrahydro-6*H*-benzo[6,7]azepino[2,3-*b*]quinoline-6-carboxylate (3am)**

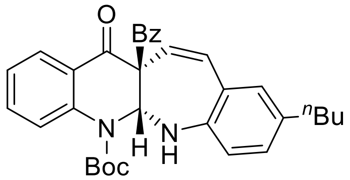

3am

The reaction was conducted with *tert*-butyl (2-(3-phenylpropioloyl)phenyl)carbamate (**1a**, 0.2 mmol, 64.2 mg), 6-butylquinoline *N*-oxide (**2n**, 0.4 mmol, 80.4 mg), 4Å

molecular sieves (40 mg) and Cu(hfacac)$_2$ (0.04 mmol, 19.1 mg) in toluene (4.0 mL) at 120 ^{o}C (120 °C, heating mantle temperature). Purification by column chromatography on silica gel (petroleum ether/ethyl acetate = 10:1) yielded **3am** (96.2 mg, 92%) as a pale yellow oil.

^{1}H NMR (400 MHz, CDCl$_3$) δ 8.06 (d, *J* = 7.8 Hz, 1H), 8.02 (d, *J* = 8.0 Hz, 2H), 7.81 (d, *J* = 8.4 Hz, 1H), 7.53 – 7.48 (m, 2H), 7.41 – 7.37 (m, 2H), 7.20 (t, *J* = 7.5 Hz, 1H), 7.06 (s, 1H), 6.91 (d, *J* = 8.1 Hz, 1H), 6.76 (d, *J* = 12.4 Hz, 1H), 6.59 (d, *J* = 8.0 Hz, 1H), 6.42 (s, 1H), 6.15 (d, *J* = 12.3 Hz, 1H), 3.95 (s, 1H), 2.54 (t, 2H), 1.61 – 1.53 (m, 2H), 1.37 – 1.32 (m, 2H), 1.19 (s, 9H), 0.93 (t, *J* = 7.3 Hz, 3H); ^{13}C NMR (100 MHz, CDCl$_3$) δ 195.3, 191.5, 151.2, 142.9, 139.0, 135.6, 135.4, 133.9, 133.4, 133.3, 132.1, 129.6, 129.1, 128.6, 128.1, 124.5, 122.7, 122.4, 122.2, 121.8, 118.5, 83.2, 71.3, 71.2, 34.6, 33.7, 27.7, 22.3, 13.9; IR (neat): 3445(br), 2925, 2853, 2064, 1867, 1703, 1657, 1634, 1558, 1506, 1458, 1235, 1013, 516, 461; HRMS (ESI) m/z: [M + H]$^+$ calcd for $C_{33}H_{35}N_2O_4$ 523.2591, found 523.2582.

***tert*-butyl 11a-benzoyl-2-(tert-butyl)-11-oxo-5,5a,11,11a-tetrahydro-6*H*-benzo[6,7]azepino[2,3-*b*]quinoline-6-carboxylate (3an)**

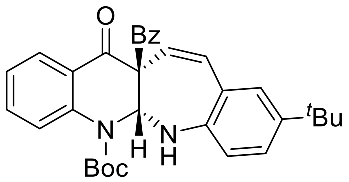

3an

The reaction was conducted with *tert*-butyl (2-(3-phenylpropioloyl)phenyl)carbamate (**1a**, 0.2 mmol, 64.2 mg), 6-(tert-butyl)quinoline *N*-oxide (**2o**, 0.4 mmol, 80.4 mg), 4Å molecular sieves (40 mg) and Cu(hfacac)$_2$ (0.04 mmol, 19.1 mg) in toluene (4.0 mL) at 120 ^{o}C (120 °C, heating mantle temperature). Purification by column chromatography on silica gel (petroleum ether/ethyl acetate = 10:1) yielded **3an** (100.4 mg, 96%) as a pale yellow oil.

^{1}H NMR (400 MHz, $CDCl_3$) δ 8.06 (d, J = 7.8 Hz, 1H), 8.02 (d, J = 7.3 Hz, 2H), 7.79 (d, J = 8.1 Hz, 1H), 7.55 – 7.48 (m, 2H), 7.42 – 7.38 (m, 2H), 7.24 – 7.18 (m, 2H), 7.13 – 7.11 (m, 1H), 6.80 (d, J = 12.4 Hz, 1H), 6.61 (d, J = 8.3 Hz, 1H), 6.44 (s, 1H), 6.14 (d, J = 12.3 Hz, 1H), 3.96 (s, 1H), 1.30 (s, 9H), 1.19 (s, 9H); ^{13}C NMR (100 MHz, $CDCl_3$) δ 195.3, 191.4, 151.2, 143.7, 142.4, 138.9, 135.6, 133.9, 133.4, 132.5, 130.3, 129.6, 128.6, 128.1, 126.2, 124.5, 122.5, 122.3, 121.9, 121.8, 118.3, 83.2, 71.2, 70.9, 34.0, 31.4, 27.8; IR (neat): 3444(br), 2065, 1868, 1656, 1650, 1644, 1633, 1507, 1477, 1417, 1315, 1118, 1010, 944, 508; HRMS (ESI) m/z: [M + H]$^+$ calcd for $C_{33}H_{35}N_2O_4$ 523.2591, found 523.2589.

***tert*-butyl 11a-benzoyl-2-methoxy-11-oxo-5,5a,11,11a-tetrahydro-6*H*-benzo[6,7]azepino[2,3-*b*]quinoline-6-carboxylate (3ao)**

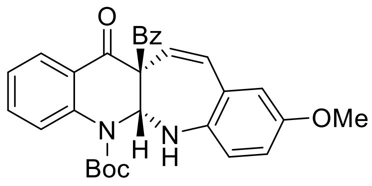

3ao

The reaction was conducted with *tert*-butyl (2-(3-phenylpropioloyl)phenyl)carbamate (**1a**, 0.2 mmol, 64.2 mg), 6-methoxyquinoline *N*-oxide (**2p**, 0.4 mmol, 70.0 mg), 4Å molecular sieves (40 mg) and Cu(hfacac)$_2$ (0.04 mmol, 19.1 mg) in toluene (4.0 mL) at 120 °C (120 °C, heating mantle temperature). Purification by column chromatography on silica gel (petroleum ether/ethyl acetate = 10:1) yielded **3ao** (77.5 mg, 78%) as a pale yellow oil.

^{1}H NMR (400 MHz, $CDCl_3$) δ 8.07 (d, J = 7.8 Hz, 1H), 8.00 (d, J = 8.0 Hz, 2H), 7.82 (d, J = 8.5 Hz, 1H), 7.55 – 7.48 (m, 2H), 7.41 – 7.37 (m, 2H), 7.20 (t, J = 7.5 Hz, 1H), 6.80 – 6.69 (m, 3H), 6.62 (d, J = 8.6 Hz, 1H), 6.38 (s, 1H), 6.22 (d, J = 12.3 Hz, 1H), 4.00 – 3.79 (m, 4H), 1.18 (s, 9H); ^{13}C NMR (100 MHz, $CDCl_3$) δ 195.2, 191.5, 154.3, 151.2, 139.2, 139.1, 135.7, 134.0, 133.4, 131.6, 129.5, 128.6, 128.1, 124.5, 124.0, 123.8, 122.1, 121.7, 119.8, 117.4, 115.4, 83.2, 71.8, 71.3, 55.7, 27.7; IR (neat):

3445(br), 2923, 2850, 2064, 1922, 1828, 1810, 1790, 1770, 1737, 1703, 1645, 1270, 507, 499; HRMS (ESI) m/z: $[M + H]^+$ calcd for $C_{30}H_{29}N_2O_5$ 497.2071, found 497.2064.

***tert*-butyl 11a-benzoyl-1-chloro-11-oxo-5,5a,11,11a-tetrahydro-6*H*-benzo[6,7]azepino[2,3-*b*]quinoline-6-carboxylate (3ap)**

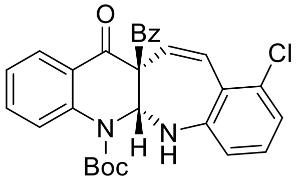

3ap

The reaction was conducted with *tert*-butyl (2-(3-phenylpropioloyl)phenyl)carbamate (**1a**, 0.2 mmol, 64.2 mg), 5-chloroquinoline *N*-oxide (**2q**, 0.4 mmol, 72.0 mg), 4Å molecular sieves (40 mg) and $Cu(hfacac)_2$ (0.04 mmol, 19.1 mg) in toluene (4.0 mL) at 120 ^{o}C (120 °C, heating mantle temperature). Purification by column chromatography on silica gel (petroleum ether/ethyl acetate = 10:1) yielded **3ap** (82.2 mg, 82%) as a pale yellow oil.

^{1}H NMR (400 MHz, $CDCl_3$) δ 7.98 (d, *J* = 7.8 Hz, 1H), 7.91 (d, *J* = 6.3 Hz, 2H), 7.78 (d, *J* = 8.3 Hz, 1H), 7.48 – 7.42 (m, 2H), 7.34 – 7.31 (m, 2H), 7.25 (d, *J* = 12.7 Hz, 1H), 7.12 (t, *J* = 6.9 Hz, 1H), 7.00 – 6.90 (m, 2H), 6.52 (d, *J* = 6.2 Hz, 1H), 6.36 – 6.31 (m, 2H), 4.00 (s, 1H), 1.14 (s, 9H); ^{13}C NMR (100 MHz, $CDCl_3$) δ 194.6, 191.1, 151.2, 147.8, 139.0, 136.4, 135.7, 134.4, 133.5, 129.4, 129.0, 128.6, 128.2, 127.0, 125.3, 124.6, 122.7, 122.0, 121.4, 120.8, 117.5, 83.4, 72.3, 70.9, 27.7; IR (neat): 3444(br), 2928, 2853, 2069, 1661, 1643, 1634, 1478, 1458, 1395, 1310, 1203, 1013, 941, 760; HRMS (ESI) m/z: $[M + H]^+$ calcd for $C_{29}H_{26}ClN_2O_4$ 501.1576, found 501.1574.

***tert*-butyl 11a-benzoyl-1-methyl-11-oxo-5,5a,11,11a-tetrahydro-6*H*-benzo[6,7]azepino[2,3-*b*]quinoline-6-carboxylate (3aq)**

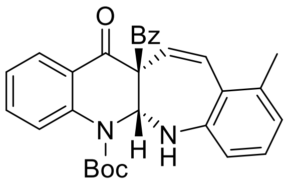

3aq

The reaction was conducted with *tert*-butyl (2-(3-phenylpropioloyl)phenyl)carbamate (**1a**, 0.2 mmol, 64.2 mg), 5-methylquinoline *N*-oxide (**2r**, 0.4 mmol, 63.6 mg), 4Å molecular sieves (40 mg) and Cu(hfacac)$_2$ (0.04 mmol, 19.1 mg) in toluene (4.0 mL) at 120 ^{o}C (120 °C, heating mantle temperature). Purification by column chromatography on silica gel (petroleum ether/ethyl acetate = 10:1) yielded **3aq** (91.4 mg, 95%) as a pale yellow oil.

^{1}H NMR (400 MHz, CDCl$_3$) δ 8.05 – 8.01 (m, 3H), 7.85 (d, *J* = 5.3 Hz, 1H), 7.55 – 7.48 (m, 2H), 7.41 – 7.37 (m, 2H), 7.21 – 7.16 (m, 1H), 7.02 – 6.95 (m, 2H), 6.83 – 6.80 (m, 1H), 6.55 (d, *J* = 7.8 Hz, 1H), 6.42 (s, 1H), 6.34 (d, *J* = 8.9 Hz, 1H), 3.96 (s, 1H), 2.46 (s, 3H), 1.21 (s, 9H); ^{13}C NMR (100 MHz, CDCl$_3$) δ 195.2, 191.5, 151.2, 146.5, 139.2, 139.0, 135.8, 134.2, 133.3, 129.5, 128.5, 128.4, 128.1, 127.7, 124.4, 123.9, 123.7, 122.2, 121.9, 121.8, 117.2, 83.1, 72.6, 71.0, 27.7, 21.0; IR (neat): 3445(br), 2926, 2852, 2064, 1867, 1828, 1688, 1657, 1644, 1558, 1473, 1160, 1010, 942, 765; HRMS (ESI) m/z: [M + H]$^+$ calcd for $C_{30}H_{29}N_2O_4$ 481.2122, found 481.2120.

***tert*-butyl 11a-benzoyl-13-chloro-11-oxo-5,5a,11,11a-tetrahydro-6*H*-benzo[6,7]azepino[2,3-*b*]quinoline-6-carboxylate (3ar)**

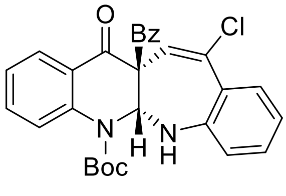

3ar

The reaction was conducted with *tert*-butyl (2-(3-phenylpropioloyl)phenyl)carbamate (**1a**, 0.2 mmol, 64.2 mg), 4-chloroquinoline *N*-oxide (**2s**, 0.4 mmol, 72.0 mg), 4Å molecular sieves (40 mg) and Cu(hfacac)$_2$ (0.04 mmol, 19.1 mg) in toluene (4.0 mL) at 120 °C (120 °C, heating mantle temperature). Purification by column chromatography on silica gel (petroleum ether/ethyl acetate = 10:1) yielded **3ar** (67.1 mg, 67%) as a pale yellow oil.

^{1}H NMR (400 MHz, CDCl$_3$) δ 8.00 (d, *J* = 5.7 Hz, 2H), 7.86 (d, *J* = 3.5 Hz, 2H), 7.77 (d, *J* = 6.9 Hz, 1H), 7.56 – 7.43 (m, 4H), 7.11 – 7.00 (m, 3H), 6.82 (s, 1H), 6.62 – 6.51 (m, 2H), 4.06 (s, 1H), 1.28 (s, 9H); ^{13}C NMR (100 MHz, CDCl$_3$) δ 194.2, 190.0, 151.4, 145.2, 139.4, 134.9, 134.4, 133.6, 133.4, 130.9, 129.9, 129.6, 128.7, 127.8, 124.5, 124.3, 123.8, 123.2, 122.8, 121.9, 120.0, 83.3, 74.9, 69.8, 27.8; IR (neat): 3445(br), 2924, 2851, 2064, 1867, 1828, 1703, 1656, 1634, 1558, 1516, 1280, 1021, 496, 489; HRMS (ESI) m/z: [M + H]$^+$ calcd for $C_{29}H_{26}ClN_2O_4$ 501.1576, found 501.1571.

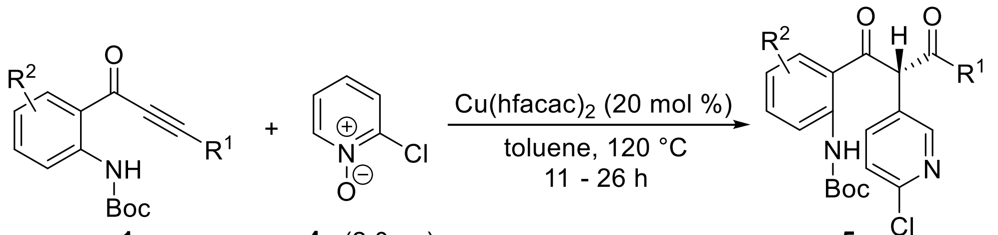

1 **4a** (2.0 eq) **5**

Supplementary Figure 77. Synthesis of pyridine-based diones **5**.

General procedure for the synthesis of pyridine-based diones 5:

2-Chloropyridine *N*-oxide **4a** (0.4 mmol, 51.8 mg) and Cu(hfacac)$_2$ (0.04 mmol, 19.1 mg) were added in this order to the alkynones **1** (0.2 mmol) in toluene (4.0 mL) at room temperature. The reaction mixture was stirred at 120 °C (120 °C, heating mantle temperature) and the progress of the reaction was monitored by TLC. Upon completion, the mixture was then concentrated and the residue was purified by chromatography on silica gel (eluent: petroleum ether/ethyl acetate) to afford the desired product **5**.

tert-butyl (2-(2-(6-chloropyridin-3-yl)-3-oxo-3-phenylpropanoyl)phenyl)carbamate (5a)

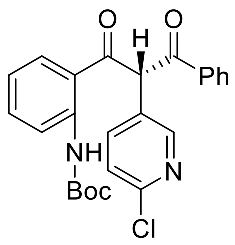

5a

The reaction was conducted with *tert*-butyl (2-(3-phenylpropioloyl)phenyl)carbamate (**1a**, 0.2 mmol, 64.2 mg), 2-chloropyridine *N*-oxide **4a** (0.4 mmol, 51.8 mg) and Cu(hfacac)$_2$ (0.04 mmol, 19.1 mg) in toluene (4.0 mL) at 120 ^{o}C (120 °C, heating mantle temperature). Purification by column chromatography on silica gel (petroleum ether/ethyl acetate = 5:1) yielded **5a** (54.1 mg, 60%) as a pale yellow oil.

^{1}H NMR (400 MHz, CDCl$_3$) δ 10.66 (s, 1H), 8.56 (d, *J* = 8.6 Hz, 1H), 8.42 (d, *J* = 2.3 Hz, 1H), 7.97 (d, *J* = 7.3 Hz, 2H), 7.82 (d, *J* = 7.4 Hz, 1H), 7.77 – 7.75 (m, 1H), 7.62 (t, *J* = 7.4 Hz, 1H), 7.56 – 7.47 (m, 3H), 7.37 (d, *J* = 8.3 Hz, 1H), 6.98 (t, *J* = 7.6 Hz, 1H), 6.67 (s, 1H), 1.49 (s, 9H); ^{13}C NMR (100 MHz, CDCl$_3$) δ 196.1, 192.5, 152.8, 151.5, 150.2, 143.5, 140.4, 135.9, 134.9, 134.2, 130.5, 129.2, 128.7, 128.2, 124.5, 121.3, 119.8, 119.1, 81.0, 59.6, 28.2; IR (neat): 3422 (br), 2977, 2925, 2853, 1731, 1703, 1681, 1524, 1452, 1417, 1367, 1251, 1024, 864, 688; HRMS (ESI) m/z: [M + H]$^+$ calcd for $C_{25}H_{24}ClN_2O_4$ 451.1419, found 451.1425.

tert-butyl (2-(2-(6-chloropyridin-3-yl)-3-(4-fluorophenyl)-3-oxopropanoyl)phenyl)carbamate (5b)

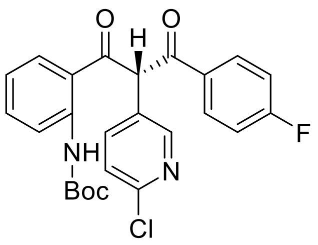

5b

The reaction was conducted with *tert*-butyl (2-(3-(4-fluorophenyl)propioloyl)phenyl)carbamate (**1b**, 0.2 mmol, 67.8 mg), 2-chloropyridine *N*-oxide **4a** (0.4 mmol, 51.8 mg) and Cu(hfacac)$_2$ (0.04 mmol, 19.1 mg) in toluene (4.0 mL) at 120 °C (120 °C, heating mantle temperature). Purification by column chromatography on silica gel (petroleum ether/ethyl acetate = 5:1) yielded **5b** (62.8 mg, 67%) as a pale yellow oil.

^{1}H NMR (400 MHz, CDCl$_3$) δ 10.63 (s, 1H), 8.57 (d, *J* = 8.6 Hz, 1H), 8.41 (d, *J* = 2.4 Hz, 1H), 8.01 – 7.98 (m, 2H), 7.81 (d, *J* = 8.1 Hz, 1H), 7.76 – 7.73 (m, 1H), 7.54 (t, *J* = 7.9 Hz, 1H), 7.38 (d, *J* = 8.4 Hz, 1H), 7.18 – 7.14 (m, 2H), 6.99 (t, *J* = 7.7 Hz, 1H), 6.61 (s, 1H), 1.50 (s, 9H); ^{13}C NMR (100 MHz, CDCl$_3$) δ 196.0, 191.0, 166.2 (d, *J* = 256.0 Hz), 152.8, 151.6, 150.2, 143.5, 140.3, 136.0, 131.5 (d, *J* = 10.0 Hz), 131.3 (d, *J* = 3.0 Hz), 130.4, 128.1, 124.5, 121.3, 119.9, 119.0, 116.5 (d, *J* = 22.0 Hz), 81.0, 59.5, 28.2; IR (neat): 3447 (br), 2957, 2924, 2853, 1732, 1681, 1659, 1523, 1454, 1368, 1250, 1107, 871, 750, 582; HRMS (ESI) m/z: [M + Na]$^+$ calcd for $C_{25}H_{22}ClFN_2NaO_4$ 491.1144, found 491.1147.

***tert*-butyl (2-(3-(4-chlorophenyl)-2-(6-chloropyridin-3-yl)-3-oxopropanoyl)phenyl)carbamate (5c)**

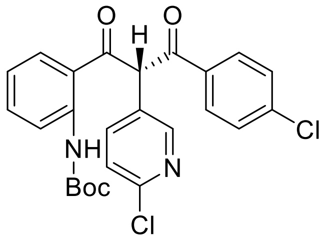

5c

The reaction was conducted with *tert*-butyl (2-(3-(4-chlorophenyl)propioloyl)phenyl)carbamate (**1c**, 0.2 mmol, 71.2 mg), 2-chloropyridine *N*-oxide **4a** (0.4 mmol, 51.8 mg) and Cu(hfacac)$_2$ (0.04 mmol, 19.1 mg) in toluene (4.0 mL) at 120 °C (120 °C, heating mantle temperature). Purification by column chromatography on silica gel (petroleum ether/ethyl acetate = 5:1) yielded **5c** (60.1 mg, 62%) as a pale yellow oil.

^{1}H NMR (400 MHz, CDCl$_3$) δ 10.62 (s, 1H), 8.57 (d, *J* = 8.6 Hz, 1H), 8.45 (s, 1H), 7.90 (d, *J* = 8.2 Hz, 2H), 7.80 (d, *J* = 8.1 Hz, 1H), 7.74 (d, *J* = 8.0 Hz, 1H), 7.55 (t, *J* = 7.8 Hz, 1H), 7.46 (d, *J* = 8.3 Hz, 2H), 7.39 (d, *J* = 8.2 Hz, 1H), 6.99 (t, *J* = 7.5 Hz, 1H), 6.61 (s, 1H), 1.50 (s, 9H); ^{13}C NMR (100 MHz, CDCl$_3$) δ 195.9, 191.4, 152.7, 150.1, 143.5, 140.9, 140.4, 136.1, 133.2, 130.5, 130.1, 129.6, 121.3, 119.9, 119.0, 81.1, 59.6, 28.2; IR (neat): 3424 (br), 2926, 2854, 1735, 1677, 1586, 1526, 1453, 1254, 1198, 1149, 1049, 852, 761, 663; HRMS (ESI) m/z: [M + Na]$^+$ calcd for $C_{25}H_{22}Cl_2N_2NaO_4$ 507.0849, found 507.0849.

***tert*-butyl (2-(3-(4-bromophenyl)-2-(6-chloropyridin-3-yl)-3-oxopropanoyl)phenyl)carbamate (5d)**

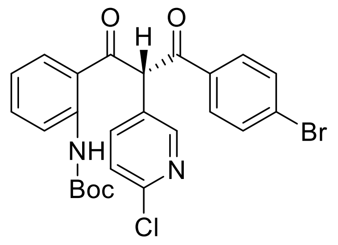

5d

The reaction was conducted with *tert*-butyl (2-(3-(4-bromophenyl)propioloyl)phenyl)carbamate (**1d**, 0.2 mmol, 80.0 mg), 2-chloropyridine *N*-oxide **4a** (0.4 mmol, 51.8 mg) and Cu(hfacac)$_2$ (0.04 mmol, 19.1 mg) in toluene (4.0 mL) at 120 °C (120 °C, heating mantle temperature). Purification by column chromatography on silica gel (petroleum ether/ethyl acetate = 5:1) yielded **5d** (70.0 mg, 66%) as a pale yellow oil.

^{1}H NMR (400 MHz, $CDCl_3$) δ 10.62 (s, 1H), 8.57 (d, J = 8.6 Hz, 1H), 8.41 (s, 1H), 7.83 – 7.79 (m, 3H), 7.74 – 7.72 (m, 1H), 7.63 (d, J = 8.3 Hz, 2H), 7.54 (t, J = 8.2 Hz, 1H), 7.38 (d, J = 8.4 Hz, 1H), 6.99 (t, J = 7.7 Hz, 1H), 6.58 (s, 1H), 1.50 (s, 9H); ^{13}C NMR (100 MHz, $CDCl_3$) δ 195.9, 191.6, 152.8, 151.7, 150.2, 143.6, 140.3, 136.1, 133.7, 132.6, 130.5, 130.1, 129.7, 128.0, 124.6, 121.3, 119.9, 119.0, 81.1, 59.6, 28.2; IR (neat): 3288 (br), 2977, 2926,2853, 1732, 1682, 1581, 1523, 1452, 1392, 1251, 1151, 1024, 805, 751; HRMS (ESI) m/z: $[M + Na]^+$ calcd for $C_{25}H_{22}BrClN_2NaO_4$ 551.0344, found 551.0344.

tert-butyl (2-(2-(6-chloropyridin-3-yl)-3-oxo-3-(4-(trifluoromethyl)phenyl)propanoyl)phenyl)carbamate (5e)

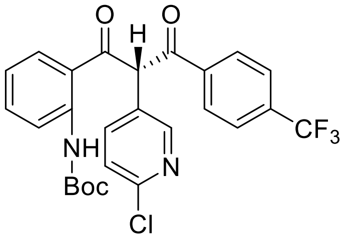

5e

The reaction was conducted with _tert_-butyl (2-(3-(4-(trifluoromethyl)phenyl)propioloyl)phenyl)carbamate (**1e**, 0.2 mmol, 77.8 mg), 2-chloropyridine _N_-oxide **4a** (0.4 mmol, 51.8 mg) and Cu(hfacac)$_2$ (0.04 mmol, 19.1 mg) in toluene (4.0 mL) at 120 °C (120 °C, heating mantle temperature). Purification by column chromatography on silica gel (petroleum ether/ethyl acetate = 5:1) yielded **5e** (65.4 mg, 63%) as a pale yellow oil.

^{1}H NMR (400 MHz, $CDCl_3$) δ 10.59 (s, 1H), 8.58 (d, J = 8.6 Hz, 1H), 8.43 (d, J = 2.1 Hz, 1H), 8.07 (d, J = 8.2 Hz, 2H), 7.82 (d, J = 8.0 Hz, 1H), 7.77 – 7.72 (m, 3H), 7.57 (t, J = 7.9 Hz, 1H), 7.39 (d, J = 8.3 Hz, 1H), 7.01 (t, J = 7.6 Hz, 1H), 6.63 (s, 1H), 1.49 (s, 9H); ^{13}C NMR (100 MHz, $CDCl_3$) δ 195.9, 191.7, 152.7, 151.8, 150.2, 143.7, 140.3, 137.7, 136.3, 135.2, 130.5, 129.0, 127.7, 126.3 (q, J = 3.5 Hz), 124.7, 121.4, 120.0, 118.8, 81.2, 59.9, 28.2; IR (neat): 3291 (br), 3086, 2979,2929, 2854, 1732,

1606, 1580, 1523, 1454, 1326, 1251, 1152, 1068, 875; HRMS (ESI) m/z: $[M + Na]^+$ calcd for $C_{26}H_{22}ClF_3N_2NaO_4$ 541.1112, found 541.1113.

***tert*-butyl (2-(2-(6-chloropyridin-3-yl)-3-oxo-3-(p-tolyl)propanoyl)phenyl)carbamate (5f)**

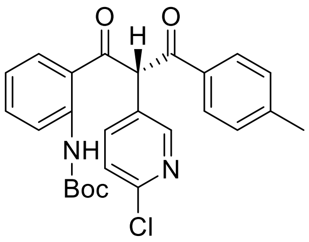

5f

The reaction was conducted with *tert*-butyl (2-(3-(*p*-tolyl)propioloyl)phenyl)carbamate (**1h**, 0.2 mmol, 64.2 mg), 2-chloropyridine *N*-oxide **4a** (0.4 mmol, 51.8 mg) and $Cu(hfacac)_2$ (0.04 mmol, 19.1 mg) in toluene (4.0 mL) at 120 °C (120 °C, heating mantle temperature). Purification by column chromatography on silica gel (petroleum ether/ethyl acetate = 5:1) yielded **5f** (69.8 mg, 75%) as a pale yellow oil.

^{1}H NMR (400 MHz, $CDCl_3$) δ 10.67 (s, 1H), 8.56 (d, *J* = 8.6 Hz, 1H), 8.41 (d, *J* = 2.1 Hz, 1H), 7.87 (d, *J* = 8.0 Hz, 2H), 7.81 (d, *J* = 8.1 Hz, 1H), 7.77 – 7.74 (m, 1H), 7.53 (t, *J* = 7.9 Hz, 1H), 7.37 (d, *J* = 8.3 Hz, 1H), 7.28 (d, *J* = 8.0 Hz, 2H), 6.97 (t, *J* = 7.6 Hz, 1H), 6.63 (s, 1H), 2.42 (s, 3H), 1.49 (s, 9H); ^{13}C NMR (100 MHz, $CDCl_3$) δ 196.2, 192.1, 152.8, 151.4, 150.2, 145.5, 143.4, 140.4, 135.8, 132.3, 130.5, 129.9, 128.9, 128.4, 124.5, 121.2, 119.8, 119.2, 80.9, 59.5, 28.2, 21.7; IR (neat): 3443 (br), 2925, 2089,1636, 1523, 1453, 1392, 1368, 1250, 1151, 1107, 1048, 750, 430, 410; HRMS (ESI) m/z: $[M + Na]^+$ calcd for $C_{26}H_{25}ClN_2NaO_4$ 487.1395, found 487.1396.

***tert*-butyl (2-(2-(6-chloropyridin-3-yl)-3-(4-ethylphenyl)-3-oxopropanoyl)phenyl)carbamate (5g)**

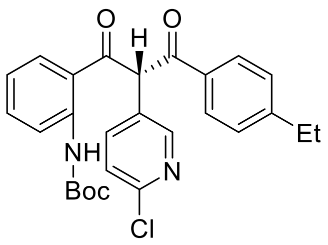

5g

The reaction was conducted with *tert*-butyl (2-(3-(4-ethylphenyl)propioloyl)phenyl)carbamate (**1i**, 0.2 mmol, 69.8 mg), 2-chloropyridine *N*-oxide **4a** (0.4 mmol, 51.8 mg) and Cu(hfacac)$_2$ (0.04 mmol, 19.1 mg) in toluene (4.0 mL) at 120 °C (120 °C, heating mantle temperature). Purification by column chromatography on silica gel (petroleum ether/ethyl acetate = 5:1) yielded **5g** (62.3 mg, 65%) as a pale yellow oil.

^{1}H NMR (400 MHz, $CDCl_3$) δ 10.68 (s, 1H), 8.56 (d, *J* = 8.6 Hz, 1H), 8.41 (d, *J* = 2.4 Hz, 1H), 7.90 (d, *J* = 8.3 Hz, 2H), 7.81 (d, *J* = 8.1 Hz, 1H), 7.77 – 7.75 (m, 1H), 7.53 (t, *J* = 7.9 Hz, 1H), 7.38 – 7.35 (m, 1H), 7.31 (d, *J* = 8.2 Hz, 2H), 6.98 (t, *J* = 7.7 Hz, 1H), 6.64 (s, 1H), 2.71 (q, *J* = 7.6 Hz, 2H), 1.49 (s, 9H), 1.27 – 1.23 (m, 3H); ^{13}C NMR (100 MHz, $CDCl_3$) δ 196.2, 192.1, 152.8, 151.6, 151.4, 150.2, 143.4, 140.5, 135.8, 132.5, 130.5, 129.0, 128.8, 128.4, 124.4, 121.2, 119.8, 119.2, 80.9, 59.5, 29.0, 28.2, 15.0; IR (neat): 3445 (br), 2922, 2850,1704, 1657, 1621, 1585, 1525, 1456, 1417, 1367, 1121, 1043, 777, 625; HRMS (ESI) m/z: [M + Na]$^+$ calcd for $C_{27}H_{27}ClN_2NaO_4$ 501.1552, found 501.1554.

***tert*-butyl (2-(3-(4-(*tert*-butyl)phenyl)-2-(6-chloropyridin-3-yl)-3-oxopropanoyl)phenyl)carbamate (5h)**

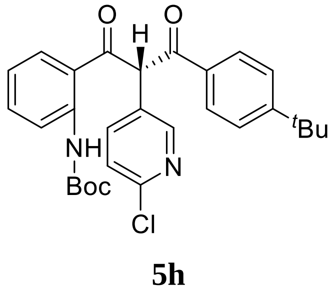

5h

The reaction was conducted with *tert*-butyl (2-(3-(4-(*tert*-butyl)phenyl)propioloyl)phenyl)carbamate (**1j**, 0.2 mmol, 75.4 mg), 2-chloropyridine *N*-oxide **4a** (0.4 mmol, 51.8 mg) and Cu(hfacac)$_2$ (0.04 mmol, 19.1 mg) in toluene (4.0 mL) at 120 ^{o}C (120 °C, heating mantle temperature). Purification by column chromatography on silica gel (petroleum ether/ethyl acetate = 5:1) yielded **5h** (66.9 mg, 66%) as a pale yellow oil.

^{1}H NMR (400 MHz, CDCl$_3$) δ 10.69 (s, 1H), 8.56 (d, *J* = 8.6 Hz, 1H), 8.41 (s, 1H), 7.92 (d, *J* = 6.9 Hz, 2H), 7.82 (d, *J* = 8.1 Hz, 1H), 7.76 (d, *J* = 8.3 Hz, 1H), 7.56 – 7.49 (m, 3H), 7.37 (d, *J* = 7.4 Hz, 1H), 6.98 (t, *J* = 7.7 Hz, 1H), 6.65 (s, 1H), 1.50 (s, 9H), 1.33 (s, 9H); ^{13}C NMR (100 MHz, CDCl$_3$) δ 196.2, 192.1, 158.4, 152.9, 151.4, 150.3, 143.4, 140.5, 135.8, 132.1, 130.5, 128.8, 128.4, 126.3, 124.5, 121.3, 119.8, 119.2, 80.9, 59.5, 35.3, 31.0, 28.2; IR (neat): 3446 (br), 2962, 2923,2852, 1721, 1682, 1646, 1581, 1525, 1454, 1367, 1252, 1153, 871, 753; HRMS (ESI) m/z: [M + Na]$^+$ calcd for $C_{29}H_{31}ClN_2NaO_4$ 529.1865, found 529.1868.

tert-butyl (2-(2-(6-chloropyridin-3-yl)-3-(3-fluorophenyl)-3-oxopropanoyl)phenyl)carbamate (5i)

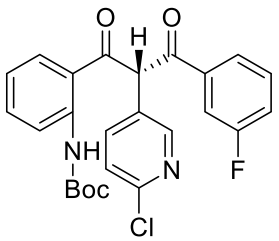

5i

The reaction was conducted with *tert*-butyl (2-(3-(3-fluorophenyl)propioloyl)phenyl)carbamate (**1l**, 0.2 mmol, 67.8 mg), 2-chloropyridine *N*-oxide **4a** (0.4 mmol, 51.8 mg) and Cu(hfacac)$_2$ (0.04 mmol, 19.1 mg) in toluene (4.0 mL) at 120 ^{o}C (120 °C, heating mantle temperature). Purification by column chromatography on silica gel (petroleum ether/ethyl acetate = 5:1) yielded **5i** (63.8 mg, 68%) as a pale yellow oil.

^1H NMR (400 MHz, $CDCl_3$) δ 10.55 (s, 1H), 8.50 (d, J = 8.6 Hz, 1H), 8.34 (s, 1H), 7.74 (d, J = 8.0 Hz, 1H), 7.68 – 7.64 (m, 2H), 7.59 (d, J = 9.2 Hz, 1H), 7.48 (t, J = 7.9 Hz, 1H), 7.43 – 7.37 (m, 1H), 7.31 (d, J = 8.2 Hz, 1H), 7.25 (t, J = 8.1 Hz, 1H), 6.93 (t, J = 7.6 Hz, 1H), 6.53 (s, 1H), 1.42 (s, 9H); ^{13}C NMR (100 MHz, $CDCl_3$) δ 195.9, 191.4, 163.0 (d, J = 248.0 Hz), 152.8, 151.7, 150.2, 143.6, 140.3, 137.0 (d, J = 6.0 Hz), 136.1, 130.9 (d, J = 8.0 Hz), 130.5, 127.9, 124.6, 124.3 (d, J = 3.0 Hz), 121.4 (d, J = 22.0 Hz), 121.3, 119.9, 118.9, 115.6 (d, J = 22.0 Hz), 81.1, 59.7, 28.2; IR (neat): 3296 (br), 2972, 2926,1719, 1686, 1582, 1525, 1455, 1393, 1297, 1252, 1153, 1023, 749, 677; HRMS (ESI) m/z: $[M + Na]^+$ calcd for $C_{25}H_{22}ClFN_2NaO_4$ 491.1144, found 491.1147.

***tert*-butyl (2-(2-(6-chloropyridin-3-yl)-3-oxo-3-(*m*-tolyl)propanoyl)phenyl)carbamate (5j)**

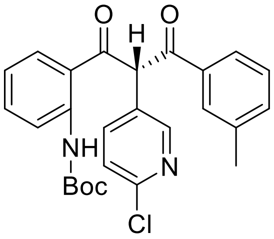

5j

The reaction was conducted with *tert*-butyl (2-(3-(m-tolyl)propioloyl)phenyl)carbamate (**1n**, 0.2 mmol, 67.0 mg), 2-chloropyridine *N*-oxide **4a** (0.4 mmol, 51.8 mg) and $Cu(hfacac)_2$ (0.04 mmol, 19.1 mg) in toluene (4.0 mL) at 120 °C (120 °C, heating mantle temperature). Purification by column chromatography on silica gel (petroleum ether/ethyl acetate = 5:1) yielded **5j** (63.2 mg, 68%) as a pale yellow oil.

^1H NMR (400 MHz, $CDCl_3$) δ 10.65 (s, 1H), 8.56 (d, J = 8.6 Hz, 1H), 8.42 (d, J = 2.3 Hz, 1H), 7.82 – 7.74 (m, 4H), 7.53 (t, J = 7.8 Hz, 1H), 7.43 (d, J = 7.5 Hz, 1H), 7.39 – 7.35 (m, 2H), 6.98 (t, J = 7.7 Hz, 1H), 6.65 (s, 1H), 2.40 (s, 3H), 1.49 (s, 9H); ^{13}C NMR (100 MHz, $CDCl_3$) δ 196.2, 192.7, 152.8, 151.5, 150.3, 143.4, 140.4, 139.3,

135.8, 135.1, 134.9, 130.5, 129.3, 129.1, 128.3, 126.0, 124.5, 121.3, 119.8, 119.2, 81.0, 59.6, 28.2, 21.4; IR (neat): 3435 (br), 2925, 2853,1732, 1681, 1581, 1524, 1453, 1368, 1251, 1150, 1049, 750, 696, 604; HRMS (ESI) m/z: $[M + Na]^+$ calcd for $C_{26}H_{25}ClN_2NaO_4$ 487.1395, found 487.1398.

***tert*-butyl (2-(2-(6-chloropyridin-3-yl)-3-(3,5-dimethylphenyl)-3-oxopropanoyl)phenyl)carbamate (5k)**

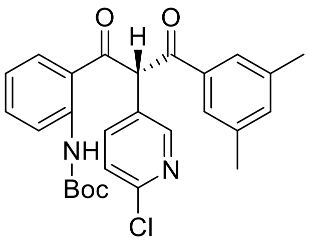

5k

The reaction was conducted with *tert*-butyl (2-(3-(3,5-dimethylphenyl)propioloyl)phenyl)carbamate (**1r**, 0.2 mmol, 69.8 mg), 2-chloropyridine *N*-oxide **4a** (0.4 mmol, 51.8 mg) and $Cu(hfacac)_2$ (0.04 mmol, 19.1 mg) in toluene (4.0 mL) at 120 °C (120 °C, heating mantle temperature). Purification by column chromatography on silica gel (petroleum ether/ethyl acetate = 5:1) yielded **5k** (61.3 mg, 64%) as a pale yellow oil.

^1H NMR (400 MHz, $CDCl_3$) δ 10.66 (s, 1H), 8.56 (d, *J* = 8.6 Hz, 1H), 8.41 (s, 1H), 7.80 – 7.74 (m, 2H), 7.57 (s, 2H), 7.53 (t, *J* = 7.9 Hz, 1H), 7.37 (d, *J* = 8.3 Hz, 1H), 7.25 (s, 1H), 6.97 (t, *J* = 7.6 Hz, 1H), 6.64 (s, 1H), 2.35 (s, 6H), 1.50 (s, 9H); ^{13}C NMR (100 MHz, $CDCl_3$) δ 196.2, 192.9, 152.8, 151.4, 150.3, 143.3, 140.5, 139.0, 136.1, 135.8, 134.9, 130.5, 128.4, 126.6, 124.5, 121.2, 119.8, 119.3, 80.9, 59.6, 28.2, 21.3; IR (neat): 3435 (br), 2923, 2852,1731, 1643, 1581, 1523, 1453, 1367, 1307, 1250, 1151, 1049, 750, 421; HRMS (ESI) m/z: $[M + Na]^+$ calcd for $C_{27}H_{27}ClN_2NaO_4$ 501.1552, found 501.1553.

tert-butyl (2-(2-(6-chloropyridin-3-yl)-3-oxo-3-(thiophen-3-yl)propanoyl)phenyl)carbamate (5l)

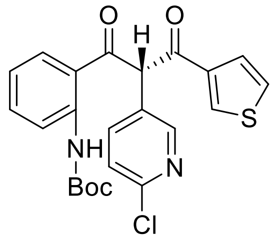

5l

The reaction was conducted with *tert*-butyl (2-(3-(thiophen-3-yl)propioloyl)phenyl)carbamate (**1s**, 0.2 mmol, 65.4 mg), 2-chloropyridine *N*-oxide **4a** (0.4 mmol, 51.8 mg) and Cu(hfacac)$_2$ (0.04 mmol, 19.1 mg) in toluene (4.0 mL) at 120 ^{o}C (120 °C, heating mantle temperature). Purification by column chromatography on silica gel (petroleum ether/ethyl acetate = 5:1) yielded **5l** (63.1 mg, 69%) as a pale yellow oil.

^{1}H NMR (400 MHz, CDCl$_3$) δ 10.66 (s, 1H), 8.56 (d, *J* = 8.6 Hz, 1H), 8.41 (s, 1H), 8.12 (s, 1H), 7.86 – 7.77 (m, 2H), 7.60 – 7.55 (m, 2H), 7.39 – 7.34 (m, 2H), 7.00 (t, *J* = 7.6 Hz, 1H), 6.48 (s, 1H), 1.50 (s, 9H); ^{13}C NMR (100 MHz, CDCl$_3$) δ 195.9, 186.4, 152.8, 151.5, 150.1, 143.4, 140.4, 140.1, 136.0, 133.8, 130.5, 128.1, 127.5, 127.1, 124.5, 121.3, 119.8, 119.2, 81.0, 61.1, 28.2; IR (neat): 3420 (br), 2920, 2849,1721, 1673, 1581, 1525, 1454, 1393, 1252, 1151, 1050, 1023, 819, 748; HRMS (ESI) m/z: [M + Na]$^+$ calcd for $C_{23}H_{21}ClN_2NaO_4S$ 479.0803, found 479.0806.

tert-butyl (4-chloro-2-(2-(6-chloropyridin-3-yl)-3-oxo-3-phenylpropanoyl)phenyl)carbamate (5m)

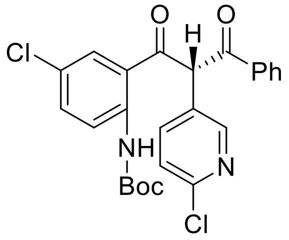

5m

The reaction was conducted with *tert*-butyl (4-chloro-2-(3-phenylpropioloyl)phenyl)carbamate (**1w**, 0.2 mmol, 71.1 mg), 2-chloropyridine *N*-oxide **4a** (0.4 mmol, 51.8 mg) and Cu(hfacac)$_2$ (0.04 mmol, 19.1 mg) in toluene (4.0 mL) at 120 ^{o}C (120 °C, heating mantle temperature). Purification by column chromatography on silica gel (petroleum ether/ethyl acetate = 5:1) yielded **5m** (63.1 mg, 65%) as a pale yellow oil.

^{1}H NMR (400 MHz, CDCl$_3$) δ 10.54 (s, 1H), 8.56 (d, *J* = 9.0 Hz, 1H), 8.44 (s, 1H), 7.96 (d, *J* = 8.1 Hz, 2H), 7.76 – 7.74 (m, 2H), 7.63 (t, *J* = 7.1 Hz, 1H), 7.53 – 7.47 (m, 3H), 7.39 (d, *J* = 8.3 Hz, 1H), 6.59 (s, 1H), 1.49 (s, 9H); ^{13}C NMR (100 MHz, CDCl$_3$) δ 195.3, 192.4, 152.6, 151.7, 150.2, 141.9, 140.4, 135.6, 134.6, 134.4, 129.7, 129.3, 128.8, 127.8, 126.1, 124.6, 121.4, 120.3, 81.4, 59.6, 28.2; IR (neat): 3391 (br), 2923, 2852,1731, 1668, 1574, 1510, 1303, 1280, 1253, 1152, 1108, 687, 653, 595; HRMS (ESI) m/z: [M + H]$^+$ calcd for $C_{25}H_{22}Cl_2N_2NaO_4$ 507.0849, found 507.0853.

***tert*-butyl (2-(2-(6-chloropyridin-3-yl)-3-oxo-3-phenylpropanoyl)-4-methylphenyl)carbamate (5n)**

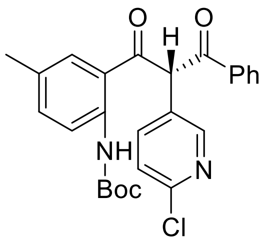

5n

The reaction was conducted with *tert*-butyl (4-methyl-2-(3-phenylpropioloyl)phenyl)carbamate (**1x**, 0.2 mmol, 67.0 mg), 2-chloropyridine *N*-oxide **4a** (0.4 mmol, 51.8 mg) and Cu(hfacac)$_2$ (0.04 mmol, 19.1 mg) in toluene (4.0 mL) at 120 ^{o}C (120 °C, heating mantle temperature). Purification by column chromatography on silica gel (petroleum ether/ethyl acetate = 5:1) yielded **5n** (61.4 mg, 66%) as a pale yellow oil.

^{1}H NMR (400 MHz, $CDCl_3$) δ 10.53 (s, 1H), 8.48 – 8.43 (m, 2H), 7.97 (d, *J* = 7.4 Hz, 2H), 7.79 – 7.76 (m, 1H), 7.64 – 7.60 (m, 2H), 7.51 – 7.47 (m, 2H), 7.39 – 7.34 (m, 2H), 6.66 (s, 1H), 2.23 (s, 3H), 1.48 (s, 9H); ^{13}C NMR (100 MHz, $CDCl_3$) δ 196.0, 192.6, 152.9, 151.4, 150.2, 141.1, 140.4, 136.9, 134.9, 134.2, 130.6, 130.5, 129.2, 128.7, 128.3, 124.5, 119.8, 119.1, 80.8, 59.6, 28.2, 20.7; IR (neat): 3435 (br), 2978, 2925,2853, 1730, 1655, 1582, 1522, 1449, 1367, 1255, 1152, 1107, 688, 672; HRMS (ESI) m/z: $[M + Na]^+$ calcd for $C_{26}H_{25}ClN_2NaO_4$ 487.1395, found 487.1400.

tert-butyl (2-(2-(6-chloropyridin-3-yl)-3-oxo-3-phenylpropanoyl)-5-fluorophenyl)carbamate (5o)

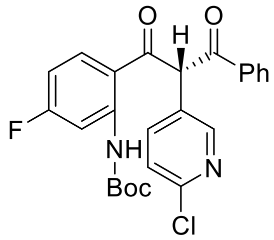

5o

The reaction was conducted with *tert*-butyl (5-fluoro-2-(3-phenylpropioloyl)phenyl)carbamate (**1aa**, 0.2 mmol, 67.8 mg), 2-chloropyridine *N*-oxide **4a** (0.4 mmol, 51.8 mg) and $Cu(hfacac)_2$ (0.04 mmol, 19.1 mg) in toluene (4.0 mL) at 120 °C (120 °C, heating mantle temperature). Purification by column chromatography on silica gel (petroleum ether/ethyl acetate = 5:1) yielded **5o** (63.8 mg, 68%) as a pale yellow oil.

^{1}H NMR (400 MHz, $CDCl_3$) δ 10.88 (s, 1H), 8.42 – 8.36 (m, 2H), 7.97 (d, *J* = 7.0 Hz, 2H), 7.82 (t, *J* = 6.4 Hz, 1H), 7.75 (d, *J* = 8.3 Hz, 1H), 7.64 (t, *J* = 7.3 Hz, 1H), 7.52 – 7.49 (m, 2H), 7.39 (d, *J* = 7.8 Hz, 1H), 6.67 (t, *J* = 8.1 Hz, 1H), 6.59 (s, 1H), 1.50 (s, 9H); ^{13}C NMR (100 MHz, $CDCl_3$) δ 194.9, 192.4, 166.9 (d, *J* = 255.0 Hz), 152.6, 151.6, 150.2, 146.3 (d, *J* = 13.0 Hz), 140.4, 134.8, 134.4, 133.1 (d, *J* = 11.0 Hz), 129.3, 128.8, 128.1, 124.6, 115.8 (d, *J* = 2.0 Hz), 109.0 (d, *J* = 23.0 Hz), 106.7 (d, *J* = 28.0 Hz), 81.5, 59.7, 28.2; IR (neat): 3281 (br), 2979, 2928,1734, 1661, 1586, 1527,

1450, 1392, 1369, 1268, 1253, 1000, 876, 687; HRMS (ESI) m/z: $[M + Na]^+$ calcd for $C_{25}H_{22}NaClFN_2O_4$ 491.1144, found 491.1144.

tert-butyl (5-bromo-2-(2-(6-chloropyridin-3-yl)-3-oxo-3-phenylpropanoyl)phenyl)carbamate (5p)

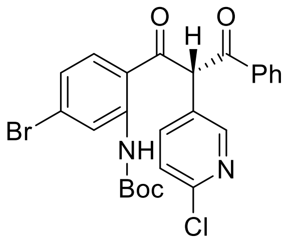

5p

The reaction was conducted with *tert*-butyl (5-bromo-2-(3-phenylpropioloyl)phenyl)carbamate (**1y**, 0.2 mmol, 80.0 mg), 2-chloropyridine *N*-oxide **4a** (0.4 mmol, 51.8 mg) and $Cu(hfacac)_2$ (0.04 mmol, 19.1 mg) in toluene (4.0 mL) at 120 ^oC (120 °C, heating mantle temperature). Purification by column chromatography on silica gel (petroleum ether/ethyl acetate = 5:1) yielded **5p** (70.0 mg, 66%) as a yellow solid (mp 126-128 °C).

^1H NMR (400 MHz, $CDCl_3$) δ 10.72 (s, 1H), 8.84 (s, 1H), 8.41 (s, 1H), 7.96 (d, J = 7.7 Hz, 2H), 7.73 (d, J = 8.3 Hz, 1H), 7.65 – 7.61 (m, 2H), 7.52 – 7.48 (m, 2H), 7.38 (d, J = 8.2 Hz, 1H), 7.11 (d, J = 8.6 Hz, 1H), 6.59 (s, 1H), 1.50 (s, 9H); ^{13}C NMR (100 MHz, $CDCl_3$) δ 195.6, 192.4, 152.5, 151.6, 150.2, 144.2, 140.4, 134.7, 134.4, 131.4, 129.3, 128.8, 127.9, 124.5, 122.6, 117.7, 81.5, 59.6, 28.2; IR (neat): 3495 (br), 2927, 2854, 1736, 1704, 1698, 1580, 1391, 1368, 1224, 1150, 1050, 806, 752; HRMS (ESI) m/z: $[M + H]^+$ calcd for $C_{25}H_{23}BrClN_2O_4$ 529.0524, found 529.0543.

tert-butyl (2-(2-(6-chloropyridin-3-yl)-3-oxo-3-phenylpropanoyl)-5-methylphenyl)carbamate (5q)

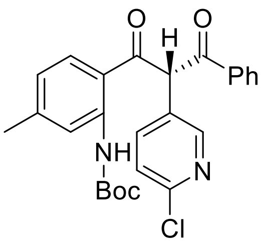

5q

The reaction was conducted with *tert*-butyl (5-methyl-2-(3-phenylpropioloyl)phenyl)carbamate (**1z**, 0.2 mmol, 67.0 mg), 2-chloropyridine *N*-oxide **4a** (0.4 mmol, 51.8 mg) and Cu(hfacac)$_2$ (0.04 mmol, 19.1 mg) in toluene (4.0 mL) at 120 ^{o}C (120 °C, heating mantle temperature). Purification by column chromatography on silica gel (petroleum ether/ethyl acetate = 5:1) yielded **5q** (60.5 mg, 65%) as a pale yellow oil.

^{1}H NMR (400 MHz, $CDCl_3$) δ 10.73 (s, 1H), 8.45 – 8.41 (m, 2H), 7.97 (d, *J* = 7.4 Hz, 2H), 7.75 – 7.71 (m, 2H), 7.62 (t, *J* = 7.4 Hz, 1H), 7.51 – 7.47 (m, 2H), 7.37 (d, *J* = 8.3 Hz, 1H), 6.80 (d, *J* = 8.2 Hz, 1H), 6.63 (s, 1H), 2.38 (s, 3H), 1.49 (s, 9H); ^{13}C NMR (100 MHz, $CDCl_3$) δ 195.6, 192.5, 152.9, 151.4, 150.2, 147.8, 143.6, 140.4, 135.0, 134.2, 130.6, 129.2, 128.7, 128.4, 124.5, 122.4, 119.8, 116.8, 80.9, 59.5, 28.2, 22.3; IR (neat): 3360 (br), 2920, 2850,1730, 1646, 1610, 1570, 1459, 1368, 1254, 1147, 1107, 1050, 861, 769; HRMS (ESI) m/z: $[M + Na]^+$ calcd for $C_{26}H_{25}ClN_2NaO_4$ 487.1395, found 487.1399.

3-(8-methylquinolin-2-yl)-2-phenyl-4*H*-chromen-4-one (3aba)

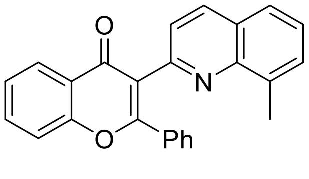

3aba

The reaction was conducted with 1-(2-hydroxyphenyl)-3-phenylprop-2-yn-1-one (**1ab**, 0.2 mmol, 44.4 mg), 8-methoxyquinoline *N*-oxide (**2a**, 0.4 mmol, 63.6 mg), 4Å molecular sieves (40 mg) and Cu(hfacac)$_2$ (0.04 mmol, 19.1 mg) in toluene (4.0 mL) at 120 ^{o}C (120 °C, heating mantle temperature). Purification by column

chromatography on silica gel (petroleum ether/ethyl acetate = 10:1) yielded **3aba** (62.4 mg, 86%) as a white solid (mp 105-107 °C).

The data of **3aba** was reported in previous literature.[3] ^{1}H NMR (400 MHz, $CDCl_3$) δ 8.32 (d, *J* = 7.8 Hz, 1H), 8.13 (d, *J* = 8.4 Hz, 1H), 7.72 – 7.67 (m, 2H), 7.62 (d, *J* = 7.5 Hz, 1H), 7.56 (d, *J* = 8.3 Hz, 1H), 7.46 – 7.36 (m, 5H), 7.28 – 7.16 (m, 3H), 2.36 (s, 3H); ^{13}C NMR (100 MHz, $CDCl_3$) δ 177.3, 164.4, 156.1, 151.9, 147.0, 137.4, 135.8, 133.8, 133.7, 129.9, 129.3, 129.1, 127.9, 127.1, 126.2(4), 126.2(0), 125.4, 125.2, 124.0, 123.2, 118.0, 17.6.

2-(8-methylquinolin-2(1*H*)-ylidene)-1,3-diphenylpropane-1,3-dione (3aca)

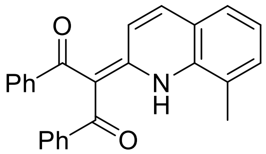

3aca

The reaction was conducted with 1,3-diphenylprop-2-yn-1-one (**1ac**, 0.2 mmol, 41.2 mg), 8-methoxyquinoline *N*-oxide (**2a**, 0.4 mmol, 63.6 mg), 4Å molecular sieves (40 mg) and Cu(hfacac)$_2$ (0.04 mmol, 19.1 mg) in toluene (4.0 mL) at 120 °C (120 °C, heating mantle temperature). Purification by column chromatography on silica gel (petroleum ether/ethyl acetate = 10:1) yielded **3aca** (64.2 mg, 88%) as a pale yellow oil.

The data of **3aca** was reported in previous literature.[4] ^{1}H NMR (400 MHz, $CDCl_3$) δ 7.96 (s, 2H), 7.62 (d, *J* = 7.4 Hz, 2H), 7.55 – 7.49 (m, 2H), 7.41 (d, *J* = 6.3 Hz, 2H), 7.33 (t, *J* = 6.8 Hz, 1H), 7.21 (t, *J* = 7.1 Hz, 1H), 7.14 – 7.10 (m, 5H), 2.78 (s, 3H); ^{13}C NMR (100 MHz, $CDCl_3$) δ 197.6, 192.6, 154.1, 142.7, 142.2, 138.8, 135.6, 132.2, 131.3, 129.6, 129.3, 128.1, 127.7(0), 127.6(6), 126.8, 125.4, 124.7, 123.6, 119.3, 106.3, 17.2.

(*Z*)-2-(8-methylquinolin-2(1*H*)-ylidene)-1-phenylhexane-1,3-dione (3aca)

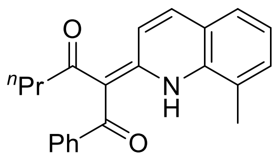

3ada

The reaction was conducted with 1-phenylhex-1-yn-3-one (**1ad**, 0.2 mmol, 34.4 mg), 8-methoxyquinoline *N*-oxide (**2a**, 0.4 mmol, 63.6 mg), 4Å molecular sieves (40 mg) and Cu(hfacac)$_2$ (0.04 mmol, 19.1 mg) in toluene (4.0 mL) at 120 ^{o}C (120 °C, heating mantle temperature). Purification by column chromatography on silica gel (petroleum ether/ethyl acetate = 10:1) yielded **3ada** (57.6 mg, 87%) as a pale yellow oil.

^{1}H NMR (400 MHz, CDCl$_3$) δ 7.93 (d, *J* = 8.3 Hz, 2H), 7.70 (d, *J* = 9.4 Hz, 1H), 7.55 – 7.43 (m, 6H), 7.29 – 7.25 (m, 1H), 7.07 (d, *J* = 9.4 Hz, 1H), 2.72 (s, 3H), 2.36 – 2.31 (m, 2H), 1.70 – 1.62 (m, 2H), 0.82 (t, *J* = 7.3 Hz, 3H); ^{13}C NMR (100 MHz, CDCl$_3$) δ 197.4, 195.7, 153.4, 141.3, 137.6, 136.0, 132.6, 132.0, 129.4, 128.7, 126.7, 125.4, 124.3, 123.1, 119.7, 106.7, 42.7, 20.1, 17.3, 14.0; IR (neat): 3444(br), 2066, 1633, 1447, 1375, 1345, 1323, 1264, 1230, 1196, 1156, 1041, 1021, 523, 499; HRMS (ESI) m/z: [M + H]$^+$ calcd for $C_{22}H_{22}NO_2$ 332.1645, found 332.1644.

Synthetic Applications

tert-butyl 4-methyl-11-oxo-5,11-dihydro-6*H*-benzo[6,7]azepino[2,3-*b*]quinoline-6-carboxylate (6)

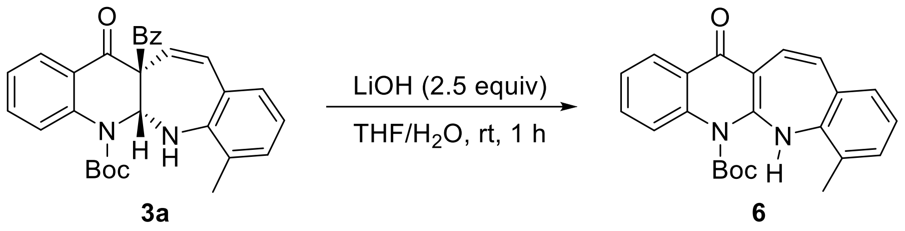

Supplementary Figure 78. Synthesis of compound **6**.

LiOH (0.5 mmol, 12.0 mg) was added to a solution of the compound **3a** (0.2 mmol, 96.2 mg) in THF/H_2O (4.0 mL/1.0 mL) at room temperature. The reaction mixture was stirred at room temperature and the progress of the reaction was monitored by TLC. Upon completion, the mixture was then concentrated and the residue was purified by chromatography on silica gel (eluent: petroleum ether/ethyl acetate) to afford the desired product **6** (74.1 mg, 99% yield, yellow oil). ^{1}H NMR (400 MHz, $CDCl_3$) δ 14.69 (s, 1H), 7.92 – 7.87 (m, 2H), 7.73 (d, *J* = 9.5 Hz, 1H), 7.61 (d, *J* = 7.7 Hz, 1H), 7.48 – 7.46 (m, 2H), 7.23 – 7.18 (m, 2H), 7.12 (t, *J* = 7.8 Hz, 1H), 2.70 (s, 3H), 1.73 (s, 9H); ^{13}C NMR (100 MHz, $CDCl_3$) δ 168.6, 149.6, 148.5, 138.5, 136.3, 133.0, 132.6, 125.7, 125.6, 125.2, 123.6, 123.2, 122.2, 118.0, 117.1, 114.6, 89.7, 83.7, 28.3, 17.0; IR (neat): 3444(br), 2850, 2065, 1845, 1634, 1508, 1489, 1458, 1400, 1368, 1316, 1260, 1021, 508, 499; HRMS (ESI) m/z: $[M + H]^+$ calcd for $C_{23}H_{23}N_2O_3$ 375.1703, found 375.1696.

tert-butyl 11a-benzoyl-4-methyl-11-oxo-5,6,11,11a-tetrahydro-5a*H*-benzo[6,7]azepino[2,3-*b*]quinoline-5a-carboxylate (7)

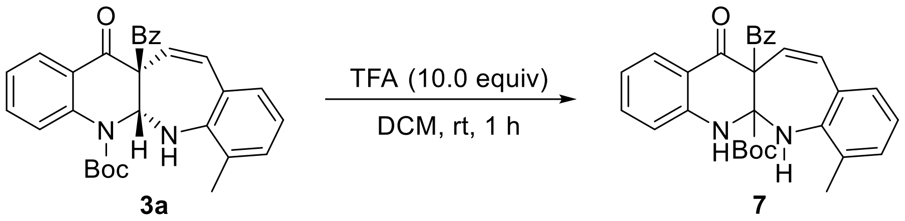

Supplementary Figure 79. Synthesis of compound **7**.

TFA (2.0 mmol, 228.0 mg) was added dropwise to a solution of compound **3a** (0.2 mmol, 96.2 mg) in DCM (4.0 mL) at 0 °C. The reaction mixture was stirred at room temperature and the progress of the reaction was monitored by TLC. Upon completion, the mixture was then concentrated and the residue was purified by chromatography on silica gel (eluent: petroleum ether/ethyl acetate) to afford the desired product **7** (64.6 mg, 67% yield, yellow oil). ^{1}H NMR (400 MHz, $CDCl_3$) δ 10.74 (s, 1H), 8.56 (d, *J* = 7.4 Hz, 1H), 8.21 (s, 1H), 8.02 (d, *J* = 6.2 Hz, 2H), 7.89 (d, *J* = 6.5 Hz, 1H), 7.77 (s, 1H), 7.58 – 7.42 (m, 6H), 6.97 (t, *J* = 5.9 Hz, 1H), 6.86 (s, 1H), 2.87 (s, 3H), 1.48 (s, 9H); ^{13}C NMR (100 MHz, $CDCl_3$) δ 196.7, 193.0, 152.9, 143.4, 137.5, 135.7, 135.2, 134.0, 130.7, 130.1, 129.2, 128.9, 127.0, 126.5, 121.2, 119.8, 119.5, 80.9, 60.7, 28.2; IR (neat): 3446(br), 2957, 2925, 2854, 1867, 1703, 1658, 1646, 1558, 1526, 1464, 1368, 1023, 662, 457; HRMS (ESI) m/z: $[M + H]^+$ calcd for $C_{30}H_{29}N_2O_4$ 481.2122, found 481.2123.

tert-butyl 11-(benzoyloxy)-4-methyl-5,5a-dihydro-6*H*-benzo[6,7]azepino[2,3-*b*]quinoline-6-carboxylate (8)

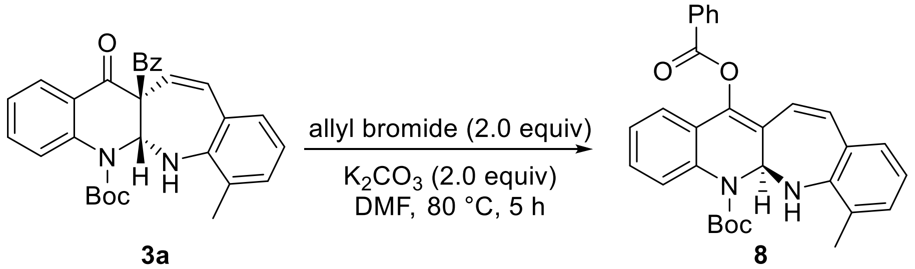

Supplementary Figure 80. Synthesis of compound **8**.

Allyl bromide (0.4 mmol, 48.4 mg), K_2CO_3 (0.4 mmol, 55.2 mg) was added to a solution of compound **3a** (0.2 mmol, 96.2 mg) in DMF (2.0 mL) at room temperature.

The reaction mixture was stirred at room temperature 80 ^{o}C and the progress of the reaction was monitored by TLC. Upon completion, the mixture was then concentrated and the residue was purified by chromatography on silica gel (eluent: petroleum ether/ethyl acetate) to afford the desired product **8** (82.7 mg, 86% yield) as a yellow solid (mp 131-133 °C). ^{1}H NMR (400 MHz, $CDCl_3$) δ 8.31 (d, *J* = 7.5 Hz, 2H), 8.02 (d, *J* = 8.3 Hz, 1H), 7.70 (t, *J* = 7.4 Hz, 1H), 7.59 – 7.55 (m, 2H), 7.33 – 7.29 (m, 2H), 7.22 (d, *J* = 7.8 Hz, 1H), 7.11 – 7.05 (m, 2H), 6.83 (t, *J* = 7.5 Hz, 1H), 6.71 (d, *J* = 11.9 Hz, 1H), 6.58 (d, *J* = 11.9 Hz, 1H), 5.21 (s, 1H), 4.49 (s, 1H), 2.31 (s, 3H), 1.40 (s, 9H); ^{13}C NMR (100 MHz, $CDCl_3$) δ 164.3, 153.1, 146.2, 136.7, 134.0, 133.6, 132.6(4), 132.5(7), 130.4, 129.6, 128.8, 127.6, 124.2, 124.0, 123.9, 123.5, 122.4, 121.5, 120.8, 119.8, 118.4, 82.9, 66.7, 28.0, 18.0; IR (neat): 3446(br), 2920, 2849, 2064, 1867, 1790, 1770, 1738, 1704, 1646, 1558, 1540, 1338, 516, 446; HRMS (ESI) m/z: $[M + H]^+$ calcd for $C_{30}H_{29}N_2O_4$ 481.2122, found 481.2124.

Computational Studies

All structures were optimized at B3LYP[5] level of theory in gas phase with Def2SVP[6] basis set for all atoms. Empirical dispersion correction has been considered by using Grimme's DFT empirical dispersion correction with the Becke-Jonson (D3BJ) damping function.[7] Optimized minima and transition states (TSs) were verified at the same level of theory by harmonic vibrational analysis to have no and one proper imaginary frequency, respectively. To refine the calculated energy, single point calculation with larger basis set were then done based on these optimized structures, by using the Def2TZVP basis set for all atoms. The solvent effect was modelled in these single point calculations by employing SMD continuum solvation model,[8] taking toluene as the solvent for each reaction. The reported free energies in this work were based on the electronic energy of single point calculations, including the Gibbs free energy thermal correction obtained from vibrational analysis in gas phase at 120℃, as well as DFT-D3(BJ) empirical dispersion correction.[7]

Cartesian coordinates for the optimized structures

A

O	0.47805900	1.71267400	-1.60283400
C	0.76102000	0.51471300	-1.72366200
C	-0.22990700	-0.48952100	-1.42024800
Cu	-2.13950900	-0.16366100	1.42297800
O	-1.09638900	1.47156800	1.17383100
C	-1.34428100	2.38345000	0.34393300
C	-0.40516700	3.60293700	0.44359300
F	0.84218400	3.23900000	0.74691100
F	-0.38073200	4.32034500	-0.67911300
F	-0.85290700	4.39619800	1.43252800
C	-2.42135900	2.45139000	-0.55190800
C	-3.38873100	1.45026000	-0.58853300
C	-4.54697100	1.57307700	-1.59552200
F	-4.51361700	0.54071100	-2.45579300
F	-5.72023800	1.54403200	-0.96036200
F	-4.48652100	2.70114000	-2.31197900
O	-3.45885400	0.40480000	0.11705100
O	-3.29858200	-1.66679300	1.88849400
C	-2.92916700	-2.67808400	2.53872000
C	-4.04293100	-3.70514600	2.83041300
F	-3.56795000	-4.96159100	2.82549900
F	-4.54414800	-3.46639500	4.05290600
F	-5.03846000	-3.62792000	1.95378300
C	-1.67154000	-2.92154100	3.10000000
C	-0.71194500	-1.90487000	3.16988100
C	0.53691700	-2.19463900	4.02647300
F	0.20663700	-2.08073400	5.32584200
F	0.98296200	-3.44515600	3.83575200
F	1.53048500	-1.34670100	3.78441800
O	-0.80720200	-0.73303400	2.72154400
C	-1.01407200	-1.38673100	-1.16522900
C	-1.87163200	-2.49576700	-0.90818900
C	-3.22263300	-2.47467900	-1.31094000
C	-4.02452300	-3.59079200	-1.09173100
C	-3.49821100	-4.72594000	-0.46720900
C	-2.16442400	-4.74225800	-0.04732000
C	-1.34736000	-3.63490000	-0.26284700
O	1.11932600	-2.19875800	1.02884600
N	2.10816500	-1.38861800	0.93727500
C	1.90412200	-0.07087800	1.15511100
C	2.93557700	0.86880500	1.07073000

C	4.20258600	0.46268400	0.73000600
C	4.44996400	-0.91171500	0.48878300
C	3.38942300	-1.86638900	0.60508900
C	3.63654300	-3.25887600	0.38173400
C	2.60535400	-4.35292400	0.50729300
C	4.93011800	-3.62214300	0.02689500
C	5.97922100	-2.69072500	-0.11425600
C	5.74402300	-1.35670500	0.11644200
N	3.02348500	2.26379100	-1.97940900
H	2.04160100	2.52536900	-1.84419000
C	3.89895900	3.23801900	-1.54414300
O	5.17541500	2.82008900	-1.50841300
C	6.25977000	3.70743300	-1.07329900
C	6.03823100	4.13722400	0.37798700
C	7.49333200	2.81439300	-1.19183400
C	6.35986700	4.90408400	-2.01964100
O	3.50788700	4.33736300	-1.21821300
C	3.20396900	0.92181900	-2.29357900
C	4.43268800	0.39648100	-2.73679700
C	4.56899500	-0.95845600	-3.01495600
C	3.49060200	-1.83872400	-2.86915400
C	2.26739300	-1.33422200	-2.45546900
C	2.08875400	0.03457400	-2.16950600
H	-2.48857700	3.29271500	-1.23483400
H	-1.47205600	-3.88032300	3.57208100
H	-3.62974800	-1.58027400	-1.77899100
H	-5.07279700	-3.57023500	-1.39614700
H	-4.13743300	-5.59205000	-0.28336900
H	-1.76281300	-5.61910500	0.46459800
H	-0.31458000	-3.60808800	0.08251500
H	0.88328500	0.19136200	1.41622300
H	2.69191500	1.91431500	1.25192300
H	5.01550600	1.17728100	0.61210800
H	1.81463200	-4.24645200	-0.24808200
H	3.09266400	-5.32933600	0.36871100
H	2.10423900	-4.32892700	1.48231400
H	5.13674700	-4.68062600	-0.14679300
H	6.97435100	-3.03747300	-0.40158600
H	6.53778300	-0.61405900	0.01561300
H	5.94624000	3.25788000	1.03422000
H	6.90286400	4.72507300	0.72163100
H	5.13293100	4.74829700	0.47338800
H	7.63548700	2.48244900	-2.23124200
H	8.39156000	3.36539300	-0.87679900
H	7.39081400	1.92354400	-0.55376700
H	5.46836600	5.53849700	-1.94543900
H	7.24642000	5.50377400	-1.76306500
H	6.46763500	4.55937400	-3.05955900
H	5.27994400	1.06526800	-2.84744900
H	5.53858600	-1.33527800	-3.34809400
H	3.60830300	-2.90389100	-3.07200100
H	1.41545100	-2.00294600	-2.33346500

TS_{B1}

O	1.44206100	1.25020700	-3.68664500
C	1.77902300	1.25910000	-2.51364500
C	1.94179800	-0.05870800	-1.81372100
Cu	0.00934500	-0.32739300	-1.00106100
O	-1.77637800	-0.79581500	-0.15854400
C	-2.76684400	-1.15825500	-0.82901200
C	-4.07970200	-1.19797300	-0.02149300
F	-4.24970200	-0.06516600	0.65618200
F	-5.15585900	-1.39436000	-0.79314200
F	-4.03015100	-2.21204700	0.86819400
C	-2.79424100	-1.58174600	-2.16862900
C	-1.61628900	-1.70708300	-2.91112800
C	-1.67680700	-2.43092800	-4.26873800
F	-2.92966800	-2.60441700	-4.70670600
F	-0.99205400	-1.77427600	-5.19893200
F	-1.12179500	-3.65537600	-4.12958200
O	-0.44613800	-1.37593500	-2.58948400
O	0.51924100	0.51392700	0.67503300
C	0.10149000	1.63057200	1.09627400
C	0.56000300	1.92787900	2.53724400
F	0.50617400	3.23087200	2.84184000
F	-0.22122900	1.27319700	3.41352300
F	1.82133700	1.51533800	2.73579400
C	-0.71114900	2.57229600	0.47654900
C	-1.23139500	2.42425700	-0.83228800
C	-2.22039400	3.52029800	-1.29525600
F	-2.27677900	3.59289100	-2.62483500
F	-3.45181500	3.23257200	-0.84208000
F	-1.88986200	4.73737600	-0.82540100
O	-1.03437600	1.50505900	-1.64045000
C	2.88437700	-0.88729700	-1.86594700
C	4.04714000	-1.56324700	-2.31737000
C	5.14297000	-1.82212600	-1.46822900
C	6.28072200	-2.43787700	-1.97809300

C	6.33893100	-2.80024100	-3.33049000
C	5.25555300	-2.54694700	-4.17836100
C	4.10816200	-1.93557400	-3.67769800
O	2.53969600	-2.69773700	-0.49877200
N	1.33407500	-3.04752500	-0.20152500
C	0.62614100	-3.73347100	-1.12267800
C	-0.67755700	-4.17115800	-0.88477800
C	-1.27787800	-3.87917600	0.31844500
C	-0.57161000	-3.13608100	1.29617600
C	0.77393800	-2.70912800	1.04288500
C	1.50343300	-1.96819300	2.02395800
C	2.91087900	-1.45743600	1.85602700
C	0.83601200	-1.67173200	3.20428300
C	-0.49710300	-2.05350200	3.45594100
C	-1.19326300	-2.77731600	2.51898800
C	1.98480600	2.47955600	-1.70408900
C	2.65135100	2.38091100	-0.47008300
C	2.81759400	3.48068800	0.35917100
C	2.28979600	4.71131500	-0.04762200
C	1.62896500	4.84881200	-1.26230700
C	1.47297600	3.74640400	-2.12940900
N	0.85317600	3.82388900	-3.36647600
C	0.10560800	4.80677800	-3.99398500
O	-0.12513600	5.86577500	-3.20958600
C	-0.90989000	7.01622100	-3.67306400
C	-2.32831900	6.57890500	-4.03991700
C	-0.92714800	7.92231500	-2.44356200
C	-0.19062500	7.68622100	-4.84389100
O	-0.28035100	4.66629700	-5.13260500
H	-3.73710700	-1.88452000	-2.61390200
H	-0.94178700	3.48419600	1.02024900
H	5.07708200	-1.54327400	-0.41675100
H	7.13013000	-2.64028700	-1.32211800
H	7.23604800	-3.28374600	-3.72451400
H	5.30600000	-2.82848600	-5.23237900
H	3.25136300	-1.72551100	-4.32064700
H	1.16275700	-3.89692500	-2.05485000
H	-1.19704900	-4.70796800	-1.67811200
H	-2.30635100	-4.17735200	0.52396200
H	2.95970900	-0.73862800	1.02855400
H	3.22249800	-0.94770500	2.77778600
H	3.61595200	-2.26547800	1.62570900
H	1.36605200	-1.08715000	3.95766500
H	-0.97159700	-1.75979500	4.39424000
H	-2.23027200	-3.07281800	2.68216800
H	3.02051300	1.40513000	-0.15786100
H	3.32904800	3.37803300	1.31670500
H	2.39305700	5.58761600	0.59711100
H	1.22643200	5.81039100	-1.55985200
H	0.92365600	2.96777700	-3.92355100
H	-2.80439300	6.07291800	-3.18917700
H	-2.92562100	7.46732900	-4.29617700
H	-2.32027900	5.89544100	-4.89721500
H	0.09388300	8.23182000	-2.17250400
H	-1.52095500	8.82574800	-2.64672700
H	-1.37124000	7.39111900	-1.58927800
H	-0.16626600	7.02462900	-5.71864600
H	-0.71421700	8.61576000	-5.11514600
H	0.84198900	7.94345300	-4.56134200

B1

O	1.35025600	0.78101300	-3.61609800
C	1.66431300	0.81455600	-2.42314100
C	1.62670900	-0.42404500	-1.62054000
Cu	-0.26829600	-0.35553200	-0.88885100
O	-2.05016600	-0.83614900	0.06097800
C	-3.08310700	-1.21706100	-0.52493900
C	-4.29967300	-1.41723900	0.40248000
F	-4.37968500	-0.45784000	1.31739900
F	-5.45983300	-1.47241900	-0.26038600
F	-4.15712500	-2.59984800	1.05729900
C	-3.23201900	-1.57461000	-1.87784500
C	-2.13052100	-1.63218700	-2.73973000
C	-2.32856900	-2.24365100	-4.14003400
F	-3.61682100	-2.45227000	-4.44092300
F	-1.79309200	-1.48011200	-5.08339800
F	-1.70739200	-3.44810800	-4.17692500
O	-0.93591400	-1.32915900	-2.50362600
O	0.32068800	0.39864200	0.84220700
C	0.08887200	1.56939900	1.25416500
C	0.66212200	1.80969800	2.66276600
F	0.65293100	3.09391700	3.03351600
F	-0.05013400	1.11608300	3.57202600
F	1.93240400	1.37168900	2.74443800
C	-0.61164100	2.60693600	0.65019600
C	-1.23219900	2.49761500	-0.61909700
C	-2.07337000	3.71755600	-1.06496400
F	-2.24088500	3.73324100	-2.38686200

F	-3.28952400	3.65944200	-0.49397500
F	-1.51362600	4.88518000	-0.69949200
O	-1.23544600	1.53242600	-1.39639900
C	2.61847900	-1.29952300	-1.52028000
C	3.99236900	-1.25498200	-2.06641100
C	5.08142200	-1.76434300	-1.33512200
C	6.37765500	-1.68549200	-1.84503700
C	6.60900000	-1.10278600	-3.09387100
C	5.53126000	-0.60863600	-3.83369800
C	4.23195400	-0.68775300	-3.33188000
O	2.51203400	-2.52741900	-0.73994800
N	1.25025700	-2.92342600	-0.41901700
C	0.57319300	-3.53357300	-1.39585300
C	-0.69850100	-4.05461800	-1.16532700
C	-1.26273200	-3.88029400	0.08022000
C	-0.56247900	-3.20775600	1.10837600
C	0.76850000	-2.71858300	0.86739400
C	1.50377400	-2.05984500	1.89615700
C	2.89656800	-1.51058500	1.75357200
C	0.84528500	-1.88615200	3.10625500
C	-0.47392000	-2.31835900	3.34867200
C	-1.17118800	-2.97805500	2.36693900
C	1.95315700	2.08897400	-1.70846900
C	2.64773100	2.04386300	-0.48824800
C	2.90877600	3.18930300	0.25239900
C	2.44706300	4.41733300	-0.23170900
C	1.76160300	4.50497700	-1.43817200
C	1.51603400	3.35300800	-2.21500800
N	0.87817300	3.37591200	-3.44700700
C	0.19873900	4.35509300	-4.14486900
O	0.05538000	5.49088200	-3.44727800
C	-0.61701500	6.66484000	-4.01046600
C	-2.06521400	6.33342100	-4.37399000
C	-0.56836300	7.66183400	-2.85383700
C	0.17521900	7.17589100	-5.21439700
O	-0.21152800	4.15850700	-5.26787800
H	-4.21060800	-1.86736600	-2.24707600
H	-0.67345100	3.55762000	1.17236700
H	4.91326200	-2.21818900	-0.35895300
H	7.21252300	-2.07956600	-1.26053800
H	7.62457800	-1.04067500	-3.49182300
H	5.69907800	-0.16539800	-4.81803100
H	3.39399300	-0.32521000	-3.92573300
H	1.08023300	-3.56871800	-2.35898800
H	-1.23001900	-4.52596400	-1.98979600
H	-2.27747600	-4.22586300	0.28293200
H	2.92731600	-0.73104800	0.98244100
H	3.20504400	-1.05439400	2.70185300
H	3.62158400	-2.28831800	1.48151700
H	1.37449500	-1.35418000	3.89804400
H	-0.93585900	-2.11453200	4.31608500
H	-2.19791200	-3.31093500	2.51785500
H	2.97433300	1.07385500	-0.11900400
H	3.44523000	3.12432500	1.19981700
H	2.62361100	5.33129900	0.34108900
H	1.41191000	5.46551200	-1.79896700
H	0.90402100	2.47304700	-3.93506800
H	-2.59582600	5.92808000	-3.50189700
H	-2.57618500	7.25311200	-4.69823800
H	-2.10725400	5.59624800	-5.18439400
H	0.47305800	7.89977200	-2.58800100
H	-1.07671400	8.59583400	-3.13580400
H	-1.06640700	7.23838300	-1.96982500
H	0.15413800	6.44613300	-6.03343900
H	-0.26058200	8.12233700	-5.56976100
H	1.22224500	7.36438500	-4.93052500

TS$_{C1}$

O	-0.18287100	0.39847600	-0.92736300
C	0.55362700	-0.62808800	-0.96711400
C	0.57884800	-1.60640600	0.01712000
Cu	-1.48248000	0.86610200	0.76370300
O	-0.14158900	2.15850100	1.41010100
C	-0.08205700	3.37046000	1.06940200
C	1.02023800	4.15551100	1.80571200
F	2.16854700	3.45933600	1.84629500
F	1.27713700	5.34537400	1.25915700
F	0.63307200	4.36121200	3.07447200
C	-0.92598400	4.07006700	0.20383900
C	-2.04922200	3.46085400	-0.37419300
C	-2.95348500	4.30621900	-1.29387100
F	-2.98991000	3.76950600	-2.51739000
F	-4.19995400	4.33602800	-0.81067200
F	-2.52631500	5.57234800	-1.41084600
O	-2.44645200	2.27972000	-0.23560100
O	-2.85121000	-0.44045900	0.21249300
C	-3.12786400	-1.52039800	0.78333800
C	-4.23712800	-2.32132900	0.07016200

F	-4.43275800	-3.52843400	0.62011300
F	-5.39106100	-1.64936500	0.13035900
F	-3.92549100	-2.50102600	-1.21906000
C	-2.57520100	-2.04128700	1.96371800
C	-1.59673900	-1.33885000	2.67000500
C	-1.09474900	-1.94229000	3.99777200
F	-1.79699800	-1.43399800	5.01953600
F	-1.23323700	-3.27670000	4.02821100
F	0.19453200	-1.65713000	4.20025000
O	-1.07605000	-0.22962200	2.38009800
C	0.53786400	-2.75932700	0.62540400
C	-0.30068900	-3.94737400	0.43387100
C	-1.15081800	-4.01323800	-0.68510600
C	-1.99632100	-5.10379700	-0.86643400
C	-2.01427000	-6.14387600	0.06973700
C	-1.17887000	-6.08196200	1.18703600
C	-0.32390800	-4.99308200	1.37192200
O	1.44990900	-2.92922700	1.77370000
N	2.46506000	-2.01316600	1.66204200
C	1.96423700	-0.74745100	1.57545400
C	2.76765900	0.30652700	1.06423500
C	3.90564300	-0.00804700	0.38386400
C	4.33653600	-1.37281400	0.27221400
C	3.60293900	-2.40441500	0.92757600
C	4.04053400	-3.75019600	0.87817400
C	3.38262200	-4.87771500	1.63298000
C	5.16982200	-4.02355000	0.10101300
C	5.87954300	-3.02871300	-0.58647600
C	5.47485600	-1.71022600	-0.48756300
N	1.88587900	1.68552400	-1.97776900
H	0.91904800	1.66349700	-1.63923100
C	2.58027100	2.80455600	-1.59055700
O	3.90390400	2.68804300	-1.78913600
C	4.82880500	3.79151200	-1.50278700
C	4.80334300	4.13272100	-0.01275400
C	6.18120700	3.20395100	-1.90142200
C	4.47778800	4.99684300	-2.37487600
O	2.02508300	3.76952700	-1.10341300
C	2.34846600	0.43462900	-2.40346400
C	3.46952600	0.28820700	-3.24138300
C	3.88830500	-0.97418800	-3.64855600
C	3.20069600	-2.12356800	-3.23920500
C	2.09718100	-1.98998700	-2.40692100
C	1.65204400	-0.72849800	-1.97804500
H	-0.70418500	5.10807400	-0.02178500
H	-2.90257700	-3.01224600	2.32245700
H	-1.16240100	-3.18542500	-1.39636800
H	-2.66224000	-5.13053600	-1.73108000
H	-2.68635300	-6.99385900	-0.06770900
H	-1.19622600	-6.88410000	1.92858600
H	0.30561400	-4.93651400	2.25836400
H	1.09144300	-0.53940200	2.19312500
H	2.39165500	1.32273800	1.15887000
H	4.49401900	0.76856000	-0.10622200
H	2.45922700	-5.21595900	1.14126000
H	4.07060800	-5.73301200	1.69190200
H	3.10716200	-4.57821800	2.65349200
H	5.51566000	-5.05865800	0.04653400
H	6.75473700	-3.29726900	-1.18129600
H	6.01560600	-0.91167900	-0.99856500
H	4.97030500	3.22973900	0.59470800
H	5.61164500	4.84579800	0.21046200
H	3.84683700	4.57761600	0.27975500
H	6.18275200	2.91485100	-2.96299100
H	6.97784200	3.94502500	-1.74081900
H	6.41126800	2.31241700	-1.29815300
H	3.49513000	5.40203800	-2.10426700
H	5.23541700	5.78335700	-2.23810700
H	4.46728600	4.71114700	-3.43802800
H	4.00147400	1.17750800	-3.56930400
H	4.75965300	-1.06162400	-4.30218100
H	3.53138700	-3.11283700	-3.56041600
H	1.56580000	-2.87590600	-2.05564300

TS_{F1}

O	1.46420900	0.97048700	-3.54450900
C	1.82647200	1.18398300	-2.38265100
C	1.79339800	0.05727600	-1.45249700
Cu	0.02814800	-0.06720500	-0.49472400
O	-1.52924500	-0.65631700	0.67706400
C	-2.61923900	-1.06550500	0.23011400
C	-3.77037300	-1.16841500	1.25527000
F	-3.32575700	-1.07542700	2.50693000
F	-4.65848300	-0.18682400	1.04910700
F	-4.42619500	-2.34000600	1.13439500
C	-2.92848500	-1.42226100	-1.09656500
C	-1.94611200	-1.45193000	-2.08904800
C	-2.35156300	-1.94228100	-3.49390300

F	-3.68112100	-2.00086400	-3.65259800
F	-1.85034600	-1.15238100	-4.43632500
F	-1.87291300	-3.19046700	-3.70165400
O	-0.72332800	-1.17168900	-1.98512200
O	0.78702400	0.84237000	1.09527600
C	0.58681100	2.04390000	1.42947700
C	1.34002700	2.41081200	2.72185100
F	1.30137000	3.71928700	3.00576300
F	0.81564400	1.74742100	3.76319500
F	2.63248700	2.05314700	2.62808000
C	-0.19134000	3.02323300	0.81953800
C	-0.94462200	2.80651700	-0.36066600
C	-1.80786100	3.99734600	-0.84387700
F	-2.06289300	3.90507300	-2.15014300
F	-2.98199400	4.00337800	-0.19161400
F	-1.21630400	5.18545500	-0.61901500
O	-1.04200600	1.77106400	-1.03555800
C	2.71451700	-0.91827500	-1.15618100
C	3.96240100	-1.25555700	-1.88884800
C	4.98543900	-1.95608600	-1.22487900
C	6.16270100	-2.28896900	-1.89408200
C	6.33417200	-1.93033100	-3.23507000
C	5.31691500	-1.24439200	-3.90492100
C	4.13440000	-0.91155800	-3.24219300
O	2.45948900	-1.58886300	-0.03997000
N	1.41779300	-3.00099700	-0.28889800
C	1.36691200	-3.43072100	-1.52648500
C	0.31639300	-4.25542200	-1.96018600
C	-0.71179200	-4.50455700	-1.07809500
C	-0.67683200	-3.99336000	0.24263500
C	0.46888800	-3.22687200	0.66639400
C	0.56398000	-2.74283500	2.00952200
C	1.74768000	-1.99152300	2.55379100
C	-0.52088800	-2.99155600	2.83831100
C	-1.67072900	-3.69451700	2.42216700
C	-1.74660200	-4.20320300	1.14716500
C	2.12353000	2.51715100	-1.82352000
C	2.87827000	2.59161600	-0.63875000
C	3.14736900	3.80731300	-0.02572200
C	2.62970500	4.97351200	-0.60030700
C	1.87458800	4.93587200	-1.76764100
C	1.61623000	3.71201700	-2.41877600
N	0.90431700	3.59945200	-3.60419200
C	0.09649400	4.47864300	-4.30528000
O	-0.05898600	5.65074900	-3.67977000
C	-0.88280800	6.72693800	-4.24224900
C	-2.32562600	6.25639000	-4.43085600
C	-0.80461700	7.80217100	-3.16007500
C	-0.25897700	7.20999600	-5.55163500
O	-0.39522000	4.16618100	-5.36680700
H	-3.94480700	-1.71711000	-1.34483300
H	-0.19705300	4.01719700	1.25791000
H	4.84628400	-2.22857400	-0.17776500
H	6.95371100	-2.82643700	-1.36559800
H	7.25737400	-2.19026500	-3.75834100
H	5.43886000	-0.97353700	-4.95620800
H	3.33264000	-0.40450900	-3.77963600
H	2.14740600	-3.07565700	-2.20509800
H	0.30069100	-4.61267400	-2.98875700
H	-1.58681400	-5.07438300	-1.39691900
H	1.71958400	-0.94253100	2.22541600
H	1.72206600	-2.01279700	3.65209200
H	2.69764700	-2.41874600	2.20924800
H	-0.47886200	-2.61488600	3.86233000
H	-2.49873200	-3.83032400	3.11945200
H	-2.62205200	-4.75976600	0.80871600
H	3.23709200	1.66702700	-0.18474100
H	3.72862900	3.84466900	0.89592000
H	2.81549300	5.94091600	-0.12703300
H	1.47967300	5.85172900	-2.19260100
H	0.93119500	2.66627600	-4.02403100
H	-2.72822700	5.86672300	-3.48618000
H	-2.94427600	7.10926600	-4.74978500
H	-2.38650800	5.46810000	-5.19043000
H	0.23349400	8.14133400	-3.02154500
H	-1.41828000	8.67034400	-3.44252900
H	-1.17168600	7.40221200	-2.20395600
H	-0.29763900	6.42347900	-6.31578600
H	-0.80769000	8.09036000	-5.92000600
H	0.79056900	7.50138300	-5.39089300

C1

O	2.20543400	0.40239800	0.85182600
C	2.33372000	-0.53093700	0.01991600
C	1.93867400	-1.86288200	0.46567900
Cu	0.01268400	0.78845300	1.41351000
O	0.28148500	2.71953300	1.72530500
C	0.03070500	3.63147900	0.90573200

C	0.16277600	5.05171500	1.49446500
F	1.27232300	5.18173300	2.20639000
F	0.14542100	6.00128600	0.54528800
F	-0.88847300	5.27442600	2.30247700
C	-0.39973600	3.49442600	-0.42947400
C	-0.50091900	2.24245600	-1.02446200
C	-0.79357500	2.15154700	-2.53278800
F	0.26225000	1.62593800	-3.17146000
F	-1.84435400	1.35498900	-2.76080300
F	-1.05200400	3.34687400	-3.08328300
O	-0.34978500	1.10988600	-0.48521700
O	-0.97436200	-0.89895200	1.16476300
C	-1.36600800	-1.66613700	2.07993200
C	-2.33086000	-2.75915600	1.58128900
F	-2.66239300	-3.62789400	2.54914500
F	-3.45794600	-2.18608600	1.14315300
F	-1.79199000	-3.44637400	0.57199600
C	-1.04715400	-1.60205200	3.44080400
C	-0.22516200	-0.58139200	3.93717100
C	0.24541900	-0.67010900	5.40192400
F	0.16573100	0.51372700	6.00424800
F	-0.47043700	-1.55265000	6.11266800
F	1.52646600	-1.07077000	5.41927300
O	0.24744600	0.40568200	3.31877300
C	1.27846400	-2.87185300	-0.16486000
C	0.68080100	-2.95374900	-1.50316800
C	0.05459100	-1.83020400	-2.06664900
C	-0.48143000	-1.90748800	-3.35107000
C	-0.40406400	-3.10073500	-4.07573700
C	0.19878700	-4.22754400	-3.50697000
C	0.73417600	-4.15955300	-2.22102900
O	1.09634900	-3.95077100	0.60939100
N	1.50700100	-3.58784800	1.98316900
C	2.24466600	-2.30685300	1.88941900
C	3.73350800	-2.41887300	2.09995800
C	4.29002400	-3.54163400	2.57322400
C	3.48139100	-4.70958400	2.91284300
C	2.10400700	-4.71621800	2.60015800
C	1.29150200	-5.82793400	2.90180300
C	-0.18096600	-5.80860200	2.60274000
C	1.89359800	-6.93546500	3.51282300
C	3.25370100	-6.94090000	3.83527100
C	4.04209300	-5.83134800	3.54230200
N	2.72979400	2.11004200	-1.06455000
H	2.55806700	1.84839700	-0.08782800
C	2.71687100	3.47439900	-1.27161200
O	2.87361500	3.81942100	-2.55133800
C	2.89396200	5.22528500	-2.98486000
C	4.05795400	5.95595200	-2.31685000
C	3.11291700	5.10250800	-4.49113200
C	1.54627700	5.87978500	-2.68442300
O	2.55120100	4.24415600	-0.34617200
C	3.02396400	1.02082900	-1.87590800
C	3.46190200	1.14612800	-3.20958700
C	3.82093800	0.02631900	-3.94941700
C	3.78014400	-1.25653600	-3.38969100
C	3.32569100	-1.39906800	-2.08994600
C	2.89679200	-0.29559800	-1.32074600
H	-0.55974100	4.38611400	-1.02578000
H	-1.41552800	-2.36778200	4.11619400
H	-0.03812700	-0.90808600	-1.49511100
H	-0.96890900	-1.02956200	-3.77869400
H	-0.82416100	-3.15686700	-5.08287200
H	0.25066000	-5.16366000	-4.06763700
H	1.20767200	-5.03330900	-1.76954300
H	1.82597600	-1.60739900	2.63211200
H	4.32983600	-1.53823300	1.84902900
H	5.37021400	-3.60904800	2.72693800
H	-0.37964100	-5.74351000	1.52321700
H	-0.67570100	-6.70610700	2.99918700
H	-0.64900000	-4.92145000	3.05268900
H	1.27525100	-7.80374200	3.75354400
H	3.69491400	-7.81370600	4.32191900
H	5.10641600	-5.82433200	3.79029800
H	5.00358700	5.42642900	-2.51011100
H	4.14114500	6.97032400	-2.73584800
H	3.90636700	6.03195900	-1.23329800
H	2.30248300	4.51685600	-4.95009000
H	3.12914800	6.10050300	-4.95304600
H	4.07058600	4.60479700	-4.70620200
H	1.38543200	5.98159800	-1.60544800
H	1.51828600	6.88085200	-3.14090600
H	0.73030800	5.28227400	-3.11718300
H	3.52526600	2.13382300	-3.65087100
H	4.16168100	0.16275000	-4.97886700
H	4.09892700	-2.12895500	-3.96216900
H	3.30437700	-2.38596400	-1.63414000

TS$_{D1}$

O	2.30986200	0.21474000	0.78350600
C	2.37614900	-0.72097800	-0.08069800
C	2.02118900	-2.04786600	0.33896200
Cu	0.15850500	0.66686700	1.38571900
O	0.46994400	2.56761000	1.77931200
C	0.18041400	3.52262800	1.02207700
C	0.37954500	4.90871700	1.66890200
F	1.53019800	4.98139800	2.32166000
F	0.33086800	5.90414400	0.76939500
F	-0.61866300	5.11428000	2.54693700
C	-0.34450400	3.45953900	-0.28274800
C	-0.50268900	2.24025400	-0.93356000
C	-0.88928600	2.23369500	-2.42358600
F	0.12558100	1.75974100	-3.15768800
F	-1.94987600	1.44391500	-2.62991700
F	-1.19106100	3.45822400	-2.88354200
O	-0.33516200	1.08114200	-0.46349400
O	-0.77510600	-1.04987300	1.06932900
C	-1.32924000	-1.75864200	1.94266700
C	-2.21916200	-2.86735600	1.34948700
F	-2.76077700	-3.64831500	2.29986800
F	-3.21932900	-2.31583000	0.65490900
F	-1.51442000	-3.64559800	0.52575400
C	-1.21556400	-1.63681800	3.33456800
C	-0.35285300	-0.68897700	3.89566100
C	-0.02898200	-0.80188400	5.39637300
F	0.02341200	0.39344700	5.97605700
F	-0.92499300	-1.55111800	6.05581400
F	1.17274100	-1.39128500	5.52961900
O	0.29209700	0.21779900	3.30747300
C	1.54582600	-3.18769700	-0.39676000
C	0.87918700	-3.00700000	-1.73345500
C	0.12072200	-1.87291400	-2.05798600
C	-0.47913100	-1.77041300	-3.31404200
C	-0.32882600	-2.79498200	-4.25232700
C	0.40452100	-3.94007400	-3.92251800
C	0.99406100	-4.05057800	-2.66409600
O	1.57477900	-4.32776000	0.09520700
N	1.40977600	-3.41639000	2.32166900
C	2.13216900	-2.27179300	1.87870900
C	3.60748900	-2.30829100	1.99989300
C	4.24920700	-3.41755800	2.48373500
C	3.48656200	-4.49788600	2.98786900

C	2.02892900	-4.42473700	2.88125400
C	1.21822500	-5.54224900	3.34810700
C	-0.25745700	-5.49284300	3.13225100
C	1.85560800	-6.61451100	3.92575400
C	3.27200600	-6.65515600	4.05400100
C	4.07321800	-5.63172600	3.58812500
N	2.65111400	1.96227400	-1.13190600
H	2.55817000	1.66478800	-0.15266500
C	2.68161000	3.32880800	-1.29060800
O	2.75104500	3.71529800	-2.56846300
C	2.82173300	5.13035300	-2.95512400
C	4.07336500	5.77054100	-2.35541900
C	2.92031400	5.05183400	-4.47729900
C	1.54173900	5.85071200	-2.53283000
O	2.62051900	4.07653900	-0.33260100
C	2.92567100	0.89395600	-1.98411300
C	3.27806500	1.05881500	-3.33837400
C	3.67152900	-0.02791600	-4.11013200
C	3.76066700	-1.30876800	-3.55486600
C	3.37659100	-1.48947900	-2.23584500
C	2.88667500	-0.43205800	-1.44268400
H	-0.52731000	4.38262500	-0.82186200
H	-1.73494400	-2.33746200	3.98047500
H	-0.02350300	-1.07431100	-1.33419200
H	-1.06823500	-0.88334100	-3.55350800
H	-0.79247900	-2.70708200	-5.23810900
H	0.51316600	-4.74973300	-4.64848500
H	1.55961900	-4.94139100	-2.38477400
H	1.70748500	-1.38164300	2.37080700
H	4.15155100	-1.41325700	1.69805700
H	5.33977400	-3.46343000	2.53456800
H	-0.46894700	-5.38276800	2.05770800
H	-0.74932800	-6.39746900	3.51406700
H	-0.69571100	-4.61046600	3.62006800
H	1.26704400	-7.46297600	4.28143000
H	3.73286000	-7.52864300	4.52216500
H	5.16028800	-5.68821900	3.67590700
H	4.96871300	5.19467800	-2.63617900
H	4.18573300	6.79280500	-2.74777300
H	4.00693800	5.81579300	-1.26161400
H	2.04651300	4.52614600	-4.89025600
H	2.95712300	6.06361500	-4.90714200
H	3.82955400	4.51137500	-4.78101100
H	1.46355500	5.91412800	-1.44189100

H	1.54415600	6.87057400	-2.94684100
H	0.66194600	5.32194300	-2.92830900
H	3.25290100	2.05241100	-3.76983600
H	3.94275900	0.13714500	-5.15590400
H	4.12111700	-2.15328300	-4.14442800
H	3.46253500	-2.47591300	-1.78730400

D1

O	-1.35404000	2.31298800	1.43207400
C	-1.81366300	1.28727900	0.93860500
C	-3.09541000	1.35973600	0.11444500
C	-4.40503900	0.83299300	0.66562900
C	-4.58509900	0.68222400	2.13628600
C	-3.96088300	1.52777100	3.06881500
C	-4.21461300	1.36849800	4.43233700
C	-5.07546800	0.36058700	4.87393200
C	-5.70512500	-0.47946000	3.94780500
C	-5.47393500	-0.31132500	2.58528000
O	-5.30053700	0.52884900	-0.10578900
C	-2.93931600	1.10200900	-1.37181600
N	-1.68295700	0.82522000	-1.90699400
C	-0.69859400	1.70044400	-1.75288100
C	0.68691900	1.31857900	-1.98038000
C	1.05153300	-0.11738000	-2.20148400
C	1.65866700	2.28380200	-1.92958500
C	1.37919700	3.66875200	-1.69843100
C	0.10356400	4.06959800	-1.45066700
C	-0.96614500	3.10856000	-1.40308700
C	-2.24697300	3.49849000	-1.09363100
C	-3.28731500	2.53970900	-0.84697100
C	-1.12964400	-0.01539000	1.00034500
C	-1.84020300	-1.19810000	0.71533800
C	-1.21562200	-2.43413100	0.68771900
C	0.15931800	-2.50352000	0.94094400
C	0.90024500	-1.35750700	1.21021300
C	0.27543900	-0.09700100	1.24637500
N	0.99122600	1.07395800	1.47053700
C	2.34112800	1.27283300	1.29496300
O	2.59793900	2.58130200	1.47793800
C	3.96602700	3.09592300	1.54581500
C	4.68749100	2.88882400	0.21265200
C	3.75180400	4.58313500	1.81825800
C	4.70794800	2.42775400	2.70488100
O	3.16090300	0.42139900	1.01953500
H	-3.29641400	2.32311300	2.73419000
H	-3.73601300	2.03584500	5.15242600
H	-5.26089700	0.23079700	5.94292600
H	-6.38093400	-1.26542700	4.29231100
H	-5.96196900	-0.94930200	1.84881200
H	-3.77837500	0.56246400	-1.79690600
H	0.44238900	-0.78463800	-1.58211400
H	0.89670100	-0.39496900	-3.25279800
H	2.10745900	-0.28987100	-1.95395000
H	2.69916300	1.97619400	-2.05549400
H	2.20014000	4.38817700	-1.69554900
H	-0.13230700	5.11263900	-1.22853200
H	-2.49636600	4.56235800	-1.07166600
H	-4.32581400	2.86632300	-0.93746200
H	-2.90198500	-1.15147200	0.49233300
H	-1.78708900	-3.32732300	0.43670100
H	0.67016500	-3.46744300	0.90726000
H	1.97324000	-1.41313800	1.37111600
H	0.41900100	1.90504400	1.62753800
H	4.13574100	3.37966100	-0.60179400
H	5.68931700	3.34168900	0.26514400
H	4.79137800	1.82166400	-0.01659700
H	3.21073400	4.72814800	2.76495000
H	4.71979100	5.10193800	1.88206200
H	3.15975700	5.03762700	1.01002400
H	4.85566200	1.35735700	2.51376600
H	5.69250700	2.90161800	2.83682900
H	4.14011500	2.54868800	3.64017900
Cu	-2.09995700	-1.16372900	-3.16248800
O	-0.98203300	-0.98978700	-4.78370800
C	-0.09335000	-1.79770600	-5.15029600
C	0.53403600	-1.44094900	-6.51201000
F	-0.41181400	-1.24221300	-7.42924900
F	1.24702000	-0.31323700	-6.38598000
F	1.35621300	-2.40039600	-6.96027300
C	0.41777000	-2.89512200	-4.44385300
C	-0.01978300	-3.17571400	-3.14232000
C	0.74525100	-4.24685700	-2.33985300
F	1.33150900	-5.14999800	-3.13615400
F	1.70813200	-3.64802200	-1.61895200
F	-0.06255700	-4.89234400	-1.49834900
O	-0.92864200	-2.60403500	-2.49466800
O	-3.53332800	-0.23264200	-4.16881900
C	-4.77145700	-0.36019700	-3.99772700

C	-5.61263900	0.47640200	-4.98146100
F	-6.92499700	0.22832800	-4.86961000
F	-5.41707100	1.77879800	-4.73608500
F	-5.24832500	0.22545200	-6.23961600
C	-5.43439500	-1.10930600	-3.01658400
C	-4.71506900	-1.81600100	-2.04587500
C	-5.49759100	-2.63202800	-0.99921000
F	-4.92075800	-2.53238100	0.20792100
F	-6.76900700	-2.23968700	-0.88329800
F	-5.49134900	-3.92640000	-1.34747300
O	-3.46784900	-1.90256900	-1.92076500
H	1.20228600	-3.50074600	-4.88798200
H	-6.51917600	-1.10174400	-2.97705300

TS$_{E1}$

O	-1.35479200	2.29039000	1.44493000
C	-1.79258200	1.27295800	0.92096700
C	-3.07175200	1.34994700	0.07440400
C	-4.37331600	0.80088600	0.66564200
C	-4.55024500	0.73945000	2.14018700
C	-3.97779100	1.68082800	3.01225900
C	-4.23426000	1.60386500	4.38213000
C	-5.04279600	0.58338300	4.88955000
C	-5.61715500	-0.35471100	4.02334000
C	-5.38578300	-0.26965700	2.65328000
O	-5.24953400	0.41294700	-0.08613300
C	-2.92081100	0.99136000	-1.36913100
N	-1.71755000	0.79404100	-1.95552400
C	-0.70658600	1.65655200	-1.73803500
C	0.66748000	1.26372100	-1.94452500
C	1.01979600	-0.17259200	-2.18438500
C	1.65597700	2.21918100	-1.86719700
C	1.37987900	3.59517300	-1.64206500
C	0.09186300	4.00134900	-1.43132800
C	-0.97571800	3.05030800	-1.39983900
C	-2.28182100	3.46809200	-1.14036300
C	-3.30705200	2.58367800	-0.76605000
C	-1.10791300	-0.02886700	0.97570500
C	-1.80899600	-1.21011600	0.66021900
C	-1.17933900	-2.44318800	0.62142800
C	0.19154600	-2.51162400	0.89794200
C	0.92224800	-1.36820200	1.20239400
C	0.29268300	-0.10975900	1.24743800
N	0.99965800	1.05847800	1.50396700
C	2.35305400	1.26276300	1.35573500
O	2.60247700	2.56882600	1.55797100
C	3.96782400	3.09090400	1.62831700
C	4.69067900	2.89182000	0.29466800
C	3.74484800	4.57604300	1.90486500
C	4.71237200	2.42325100	2.78593400
O	3.17935300	0.41544000	1.08814300
H	-3.34747900	2.48002800	2.62416700
H	-3.79764100	2.34420700	5.05598200
H	-5.22930000	0.51901900	5.96427500
H	-6.24969500	-1.15170500	4.42048900
H	-5.83007000	-0.98505400	1.96085800
H	-3.79367200	0.50864000	-1.79687600
H	0.38996200	-0.84500400	-1.59248500
H	0.88494600	-0.42789700	-3.24467800
H	2.06818400	-0.36438500	-1.91921900
H	2.69490200	1.89965900	-1.97514000
H	2.20046400	4.31454300	-1.61526300
H	-0.14194100	5.04677200	-1.21818300
H	-2.53786900	4.52063000	-1.29057300
H	-4.34881300	2.90155000	-0.84764700
H	-2.86774500	-1.16745800	0.42155400
H	-1.74315800	-3.33358500	0.34469700
H	0.70701100	-3.47288600	0.85524900
H	1.99196000	-1.42281200	1.38382400
H	0.42367700	1.88749600	1.65675500
H	4.13113400	3.37376800	-0.51971700
H	5.68635200	3.35818700	0.34597000
H	4.80878900	1.82603600	0.06600800
H	3.20004300	4.71504900	2.85033400
H	4.70992500	5.09960200	1.97355200
H	3.15309600	5.03008700	1.09618400
H	4.86394400	1.35383600	2.59240300
H	5.69522400	2.90050200	2.91859400
H	4.14434500	2.54013000	3.72163500
Cu	-2.13636900	-1.19573900	-3.23771200
O	-1.01880800	-0.97614900	-4.84964900
C	-0.10969600	-1.75786900	-5.22279800
C	0.52102400	-1.36342300	-6.57266900
F	-0.42176500	-1.16551100	-7.49325300
F	1.21174500	-0.22564800	-6.42023000
F	1.36411700	-2.29925800	-7.03223400
C	0.41907700	-2.85758000	-4.53241300
C	-0.02218900	-3.17085100	-3.23995500

C	0.76294400	-4.23398200	-2.44663700
F	1.36880900	-5.11712600	-3.25046500
F	1.71236800	-3.62202200	-1.71838700
F	-0.03230900	-4.90321900	-1.61199400
O	-0.94980000	-2.63150000	-2.59092800
O	-3.59724400	-0.27773000	-4.21415200
C	-4.83001400	-0.41139600	-4.01386800
C	-5.69901900	0.41849400	-4.97944100
F	-7.00709800	0.16337300	-4.83551000
F	-5.50526000	1.72246000	-4.74259600
F	-5.36349400	0.16538800	-6.24504500
C	-5.46670300	-1.16438800	-3.01753500
C	-4.72257500	-1.87027300	-2.06549500
C	-5.47641400	-2.68817800	-0.99903800
F	-4.88444900	-2.56631800	0.19931600
F	-6.75217700	-2.31601400	-0.86933600
F	-5.45268500	-3.98582000	-1.33351500
O	-3.47208100	-1.95216100	-1.97101300
H	1.22102300	-3.43700000	-4.98043700
H	-6.55034300	-1.16250200	-2.95387800

E1

O	0.69121600	-2.60421700	-2.82774700
C	0.43533900	-1.91970000	-1.86625600
C	1.62592400	-1.46572100	-0.94543200
C	1.54615600	-2.41812400	0.27376000
C	2.47559200	-2.28976100	1.43642100
C	3.68205400	-1.56931400	1.41999800
C	4.53756100	-1.60880900	2.52177600
C	4.18328600	-2.33482400	3.66022600
C	2.96768100	-3.02760700	3.69812400
C	2.12480000	-3.01431900	2.59127900
O	0.71277600	-3.29982400	0.24737400
C	1.58102800	-0.01620500	-0.56039600
N	1.36051300	0.92963300	-1.40666300
C	1.24316700	0.69527100	-2.80278800
C	0.14460800	1.20147700	-3.53256700
C	-1.14464800	1.59820900	-2.86976700
C	0.22164500	1.19165600	-4.92891700
C	1.33549000	0.67827100	-5.59720100
C	2.35457000	0.07172400	-4.87216800
C	2.30112200	0.01593400	-3.46808200
C	3.24055800	-0.86370000	-2.77840000
C	2.95685700	-1.58526300	-1.67560500
C	-0.99425700	-1.56109200	-1.54862500
C	-1.91842300	-2.01989900	-2.50886500
C	-3.28197000	-1.78168600	-2.39207800
C	-3.76505200	-1.06964600	-1.29016700
C	-2.87093400	-0.60863800	-0.32877600
C	-1.49320100	-0.84222700	-0.44074700
N	-0.65728200	-0.26539800	0.56929500
C	-0.77994600	-0.68095500	1.89342000
O	-0.30696600	0.29967800	2.68232800
C	-0.58857400	0.32600900	4.12225900
C	0.17653700	-0.79719500	4.81948100
C	-0.07781600	1.70034600	4.55097200
C	-2.09984800	0.23249400	4.34615100
O	-1.21713000	-1.74947900	2.24683600
H	3.96955500	-0.97407400	0.55396800
H	5.47300800	-1.04769500	2.49749500
H	4.85137100	-2.35397700	4.52467300
H	2.67970100	-3.58163300	4.59429500
H	1.17640100	-3.55256400	2.59044200
H	1.85087200	0.26262800	0.45729100
H	-1.87036500	0.77693800	-2.98197800
H	-1.02443600	1.77916200	-1.79981500
H	-1.57616300	2.48996600	-3.34028700
H	-0.61963200	1.59039700	-5.50092300
H	1.38061700	0.70651500	-6.68798900
H	3.18213300	-0.42101000	-5.38739000
H	4.19247000	-1.05189000	-3.28387600
H	3.68834500	-2.29948800	-1.29481600
H	-1.51758600	-2.57411000	-3.35745800
H	-3.96836200	-2.15108800	-3.15708900
H	-4.83350600	-0.87028300	-1.18115600
H	-3.22900600	-0.03767800	0.52898600
H	-0.59560000	0.75701800	0.51675300
H	1.24607600	-0.73588100	4.57505500
H	0.06096600	-0.70177500	5.91034000
H	-0.20091300	-1.77843200	4.50591200
H	-0.55143000	2.48723200	3.94780200
H	-0.31513600	1.87356400	5.61110600
H	1.00957100	1.76928400	4.42750700
H	-2.49040400	-0.74397900	4.03387100
H	-2.32111100	0.37346000	5.41480000
H	-2.61424900	1.02403100	3.78026300
Cu	1.49164500	2.82448400	-0.62705900
O	-0.45468200	2.67760200	0.40870200

C	-1.30394000	3.57168200	0.19392300
C	-2.41934100	3.63168800	1.25756200
F	-2.95428800	2.40797400	1.42970900
F	-1.90414300	4.01673800	2.43525700
F	-3.41119000	4.47203800	0.94726700
C	-1.34433300	4.48942100	-0.87052700
C	-0.41733400	4.50043700	-1.92410800
C	-0.67512000	5.46444100	-3.10152000
F	-1.66502600	6.33279600	-2.85543000
F	0.42593400	6.15875200	-3.38711600
F	-1.01652700	4.75691600	-4.19381100
O	0.60372700	3.78803300	-2.10169500
O	2.68116300	2.18773500	0.83077200
C	2.80969000	2.76186600	1.94706100
C	3.53498500	1.89523000	2.99091100
F	4.75509500	1.56614200	2.54867400
F	3.67349900	2.51057900	4.17148500
F	2.84480800	0.76552900	3.19724700
C	2.38776900	4.03876900	2.31706300
C	1.82492200	4.90067200	1.36296500
C	1.31350800	6.28302800	1.81137100
F	-0.02598200	6.29258200	1.72807600
F	1.65103800	6.56823000	3.07548200
F	1.79017100	7.24505400	1.02229300
O	1.65051500	4.66977400	0.14567700
H	-2.15814200	5.20761900	-0.90173500
H	2.52621900	4.37895100	3.33857100

TS$_{3a}$

O	0.93407400	-2.84016500	-2.85775700
C	0.57107900	-2.24845200	-1.87003600
C	1.59294000	-1.61559500	-0.88727700
C	1.80936900	-2.70357200	0.20995800
C	2.98150900	-2.64689500	1.13415000
C	3.82094200	-1.53174300	1.29916700
C	4.89051000	-1.58396800	2.19354500
C	5.13669300	-2.74482200	2.92960500
C	4.30098000	-3.85724200	2.78021900
C	3.22969600	-3.80561700	1.89380900
O	1.00459900	-3.61194200	0.28102000
C	1.01271300	-0.36868300	-0.22134000
N	0.76630600	0.81937800	-1.01573600
C	0.66282900	0.75129100	-2.42735500
C	-0.49673100	1.28434500	-3.04211000
C	-1.68260400	1.72441100	-2.22420100
C	-0.53883800	1.37895200	-4.43584900
C	0.53378700	0.96217700	-5.22656000
C	1.66158000	0.41684800	-4.62259600
C	1.73644900	0.28325100	-3.22708100
C	2.90103200	-0.39503600	-2.65183800
C	2.87132800	-1.24383300	-1.61201500
C	-0.86971000	-2.15964900	-1.49877900
C	-1.78653300	-2.90342600	-2.25806100
C	-3.14210800	-2.89769700	-1.95189600
C	-3.60249300	-2.13152900	-0.87516900
C	-2.71330300	-1.36943500	-0.11883000
C	-1.34927700	-1.39146600	-0.42818800
N	-0.44591700	-0.51239900	0.29888000
C	-0.55014900	-0.49351400	1.76789900
O	-1.55594900	0.27489600	2.10347200
C	-1.79648300	0.70226000	3.51700900
C	-0.59849500	1.53936000	3.95459900
C	-3.05697700	1.54597600	3.38794000
C	-2.02385500	-0.52767600	4.38961600
O	0.24603500	-1.07091000	2.44997600
H	3.64402000	-0.60622600	0.75356000
H	5.52274200	-0.70524400	2.32634600
H	5.97808100	-2.78113700	3.62584300
H	4.48698400	-4.76513300	3.35853100
H	2.56080800	-4.65740000	1.76514400
H	1.59116200	-0.11336700	0.66860800
H	-2.29400900	0.85615000	-1.92157200
H	-1.38435500	2.25211300	-1.30998700
H	-2.32807400	2.39607000	-2.80593100
H	-1.42695600	1.80461600	-4.90816300
H	0.48523400	1.06090200	-6.31307200
H	2.49893800	0.06730900	-5.23107300
H	3.84878100	-0.27244700	-3.18574400
H	3.78293400	-1.76778300	-1.32653500
H	-1.38635800	-3.48746600	-3.08792700
H	-3.84278200	-3.48532400	-2.54834600
H	-4.66601600	-2.11285700	-0.62744000
H	-3.07275600	-0.74270800	0.69490200
H	-0.45813100	0.55945000	-0.23884500
H	-0.40930400	2.33841900	3.22318100
H	-0.81673800	2.00162600	4.92863500
H	0.29693500	0.91670200	4.06401500
H	-3.90109700	0.93526700	3.03311700

H	-3.32206600	1.96486100	4.36955200
H	-2.89580300	2.37447900	2.68730700
H	-1.11801200	-1.14117200	4.46833300
H	-2.31273900	-0.19713300	5.39861900
H	-2.84189500	-1.14458200	3.98699200
Cu	1.50552500	2.54722800	-0.31556000
O	-0.27280500	3.28926000	0.87019500
C	-0.73330200	4.40315600	0.54731300
C	-1.54196600	5.10927300	1.65900100
F	-2.75602900	4.53116500	1.78338800
F	-0.91661600	4.98747200	2.83708100
F	-1.74216000	6.41083800	1.42865200
C	-0.63713000	5.06826300	-0.69192400
C	0.08108800	4.57853900	-1.78906200
C	-0.08366600	5.30950500	-3.13711300
F	-0.63878100	6.52358100	-3.00769800
F	1.08900600	5.45442900	-3.75221100
F	-0.88717100	4.58248000	-3.93772200
O	0.83182700	3.57398200	-1.87242500
O	2.38418800	1.71056200	1.28561700
C	2.68734300	2.35692300	2.32676700
C	3.13397300	1.49236100	3.52235700
F	4.46766800	1.31990200	3.47549500
F	2.84060200	2.07239200	4.69490200
F	2.56078900	0.28952700	3.50290700
C	2.73262900	3.73988700	2.50344100
C	2.56976300	4.61143700	1.41300400
C	2.61759200	6.13068200	1.66781600
F	1.37920100	6.63283500	1.52699400
F	3.04369400	6.43273500	2.90210900
F	3.41586700	6.73680000	0.79017200
O	2.35274800	4.30123600	0.22268700
H	-1.15299100	6.01701800	-0.80716200
H	2.95501900	4.14576600	3.48604100

3a

O	0.13981600	-2.83531400	-2.84313800
C	0.00312900	-2.24977300	-1.79611900
C	1.18962000	-1.82889100	-0.90554500
C	1.16028700	-2.79510200	0.32098800
C	1.94934000	-2.46215200	1.54887200
C	3.04787600	-1.58677700	1.56284200
C	3.70749800	-1.30089600	2.75892600
C	3.27886700	-1.88800700	3.95155300
C	2.19458300	-2.77376800	3.94496500
C	1.53722400	-3.06317500	2.75176100
O	0.45981900	-3.78394300	0.27544700
C	0.86616900	-0.41841200	-0.38176600
N	0.56075000	0.62527700	-1.38291200
C	1.19474700	0.78604300	-2.64974600
C	0.52455800	1.63545000	-3.56378200
C	-0.91559500	2.02605300	-3.35274700
C	1.20147400	2.06801000	-4.70442900
C	2.50559300	1.64711400	-4.97106400
C	3.10116500	0.71620000	-4.13206200
C	2.45118000	0.22599400	-2.97975200
C	3.07587700	-0.93822100	-2.34922000
C	2.54400800	-1.87785400	-1.54987200
C	-1.33161700	-1.84679400	-1.25378000
C	-2.47790400	-2.29219200	-1.92804400
C	-3.74862200	-1.94076400	-1.48795100
C	-3.87555900	-1.11060000	-0.36797100
C	-2.75308200	-0.64418400	0.31327200
C	-1.47016700	-1.02103700	-0.10880200
N	-0.29138600	-0.50402500	0.49041100
C	-0.16472600	0.14972800	1.71528600
O	-1.09011100	-0.19569700	2.58402300
C	-1.20936700	0.48198900	3.89994600
C	-1.58014700	1.94256400	3.66532800
C	-2.35257600	-0.28732400	4.55354200
C	0.08440900	0.31996400	4.69572300
O	0.76017800	0.91945700	1.91205800
H	3.40962200	-1.13738400	0.64021000
H	4.55769500	-0.61616800	2.75398700
H	3.79050800	-1.65697100	4.88882500
H	1.86132200	-3.23726900	4.87639600
H	0.68838000	-3.74800600	2.72397900
H	1.70958700	-0.07315200	0.20988900
H	-1.58190200	1.16669100	-3.54762400
H	-1.12424900	2.37515800	-2.33621000
H	-1.20200200	2.83517800	-4.03609300
H	0.69556700	2.75545700	-5.38545700
H	3.03554900	2.01501300	-5.85172500
H	4.08699400	0.31442300	-4.37598300
H	4.09648400	-1.12335600	-2.69572500
H	3.16115500	-2.74763600	-1.31314000
H	-2.32660100	-2.92331100	-2.80544700
H	-4.63527300	-2.29729600	-2.01574400

H	-4.86536800	-0.80028000	-0.02530100
H	-2.86869600	0.03289000	1.15266400
H	-0.45060400	0.68842700	-1.49178500
H	-2.53598400	2.01937400	3.13056100
H	-1.68863800	2.44944100	4.63582800
H	-0.80790700	2.46951600	3.09318300
H	-2.08541800	-1.34709300	4.67869700
H	-2.57187900	0.13979200	5.54287900
H	-3.26256200	-0.22798700	3.93857400
H	0.89075400	0.93681800	4.28530900
H	-0.09960600	0.62572500	5.73685500
H	0.40661700	-0.73114600	4.69424900
Cu	0.91136500	2.39672800	-0.13928300
O	-1.03891400	2.57725600	0.10173400
C	-1.69103100	3.63474200	-0.15215300
C	-3.05306500	3.70346200	0.56380400
F	-3.60346000	2.48473300	0.66807300
F	-2.89076000	4.18515200	1.80649600
F	-3.92848500	4.49472400	-0.06808600
C	-1.34128400	4.70528900	-0.96715700
C	-0.10950800	4.77447700	-1.67153200
C	0.04796600	5.90313900	-2.71576000
F	-0.83941200	6.89547000	-2.55151100
F	1.27076100	6.42711000	-2.67578800
F	-0.14576500	5.38497200	-3.94514600
O	0.84832400	3.99088700	-1.61142600
O	2.81145900	1.93643600	-0.19747800
C	3.62925600	2.21123000	0.72120900
C	5.04208600	1.64486900	0.47774600
F	4.98494800	0.44033500	-0.10653800
F	5.73190600	2.46015000	-0.32510500
F	5.73504900	1.50575600	1.62145600
C	3.42545400	2.97591400	1.87049700
C	2.20920700	3.64133100	2.11667900
C	2.05555500	4.39691000	3.45309100
F	1.26271800	3.68335300	4.27366500
F	3.22620000	4.58013500	4.07964800
F	1.49160700	5.58851600	3.26541100
O	1.19325100	3.68165700	1.39250300
H	-2.06075700	5.50818500	-1.10124800
H	4.23513500	3.07499100	2.58740900

F1

O	1.82913600	1.37900500	-3.10565900
C	2.10828100	1.65881000	-1.92701600
C	2.11378000	0.34664700	-1.30596000
Cu	0.24251200	-0.13211200	-0.61740400
O	-1.57139900	-0.52783500	0.07829100
C	-2.43110500	-1.21473400	-0.51361300
C	-3.83404900	-1.21019400	0.12980100
F	-3.79100600	-0.86389600	1.41237100
F	-4.61448200	-0.33105700	-0.51675200
F	-4.42005500	-2.41742100	0.03818800
C	-2.28553200	-1.93206000	-1.71393700
C	-1.08102200	-1.94685600	-2.41666600
C	-1.04777300	-2.73690000	-3.74061000
F	-1.78787000	-3.85491700	-3.66933100
F	-1.53861000	-1.98351100	-4.73328600
F	0.19868000	-3.09051600	-4.07079400
O	-0.00389200	-1.35626500	-2.13532400
O	0.62879600	0.83668200	1.04877500
C	0.09614800	1.91414400	1.43833500
C	0.42094500	2.20552800	2.91520200
F	-0.03471000	3.39410600	3.33159000
F	-0.11611500	1.26097900	3.70200100
F	1.74997900	2.18707800	3.11102800
C	-0.69008400	2.82279600	0.73974600
C	-1.03609700	2.66949100	-0.62701000
C	-1.96508900	3.76577700	-1.20544300
F	-1.96690400	3.74452400	-2.53921200
F	-3.22380600	3.57734400	-0.78287100
F	-1.58611600	4.99841400	-0.81330500
O	-0.71030800	1.76409500	-1.40653100
C	3.30851200	-0.47491600	-1.22725600
C	3.74420800	-1.46530100	-2.20343000
C	4.91068600	-2.20442300	-1.94016100
C	5.32097800	-3.17920700	-2.84355600
C	4.56952600	-3.41566700	-4.00449700
C	3.40808300	-2.68144300	-4.26306500
C	2.98943100	-1.70151600	-3.36380200
O	3.84124700	-0.19359500	-0.15166800
N	1.84638300	-3.11630900	0.03765400
C	1.82040300	-3.90671600	-1.01269000
C	0.69325600	-4.67658800	-1.39392600
C	-0.44851200	-4.59060000	-0.63096200
C	-0.46117000	-3.75986300	0.51901700
C	0.73694200	-3.03247600	0.82728500
C	0.78312900	-2.20494100	1.99797100

C	2.06515200	-1.53247300	2.39365900
C	-0.36550300	-2.08713600	2.76233200
C	-1.55248800	-2.78750200	2.44575500
C	-1.60262600	-3.62152300	1.35044300
C	2.20856900	2.94097300	-1.27896500
C	2.72031600	2.97086900	0.03667500
C	2.76032400	4.15394500	0.75347600
C	2.26527700	5.31987900	0.15111500
C	1.74354400	5.31979100	-1.13767300
C	1.70526400	4.13097700	-1.89411000
N	1.20296900	4.04262600	-3.17943100
C	0.45035700	4.91484800	-3.96296200
O	0.12751800	6.03534800	-3.31894200
C	-0.70031400	7.08098800	-3.94197300
C	-2.06217300	6.51168100	-4.33832300
C	-0.84825100	8.09818000	-2.81294700
C	0.04960400	7.67462400	-5.13356800
O	0.15186200	4.62027200	-5.09658200
H	-3.12486300	-2.51315200	-2.08821700
H	-1.03994500	3.70742800	1.26524200
H	5.46396500	-2.00831800	-1.02009500
H	6.22237300	-3.76365600	-2.64742800
H	4.89202800	-4.18611400	-4.70917700
H	2.81661900	-2.88158200	-5.15808100
H	2.07017600	-1.14360400	-3.53688000
H	2.72924100	-3.95095800	-1.62065300
H	0.73773300	-5.30115300	-2.28733800
H	-1.35096400	-5.14531000	-0.89961900
H	2.49092100	-0.94437300	1.57337600
H	1.90834300	-0.86680500	3.25227300
H	2.82267000	-2.28814300	2.65962300
H	-0.35089400	-1.43098700	3.63470500
H	-2.43448700	-2.65659000	3.07540800
H	-2.51375400	-4.16905500	1.09953100
H	3.06745000	2.03855400	0.48391700
H	3.14591200	4.16904500	1.77300200
H	2.27865600	6.26096900	0.70663700
H	1.35569700	6.23450400	-1.57102800
H	1.31010800	3.13647900	-3.63547700
H	-2.55207400	6.04962900	-3.47063900
H	-2.70063300	7.32969600	-4.70516700
H	-1.96201500	5.75796700	-5.12832000
H	0.13288000	8.50146800	-2.51884800
H	-1.48304500	8.93518700	-3.13878000
H	-1.31082400	7.62332900	-1.93550800
H	0.17086400	6.93061600	-5.93088200
H	-0.51349600	8.53217600	-5.53227000
H	1.04351800	8.03140300	-4.82220100

TS_{G1}

O	3.16047700	-0.30701400	-2.67007800
C	2.02505000	-0.64379500	-2.33494200
C	0.99224600	0.28118600	-2.80978600
C	1.14447800	1.78057300	-2.83947200
C	1.61443300	2.51842300	-1.62041800
C	2.48430000	2.00057700	-0.64779000
C	2.86122100	2.78843500	0.44163200
C	2.36191500	4.08430000	0.58476900
C	1.48810000	4.60398300	-0.37620500
C	1.13100000	3.83299200	-1.47896600
O	0.78880600	2.40223100	-3.82671600
Cu	-0.90443300	0.24315900	-1.67735000
O	-2.33553500	-1.05572700	-0.99351600
C	-3.19889900	-1.56489400	-1.73913900
C	-4.02084800	-2.70345100	-1.09826000
F	-4.22356800	-2.48876000	0.19611600
F	-5.20772400	-2.87298300	-1.69391200
F	-3.33705200	-3.86305600	-1.22932800
C	-3.45473200	-1.26783800	-3.08852600
C	-2.74910300	-0.27459900	-3.76926300
C	-3.19309400	0.03072900	-5.21545400
F	-4.24829700	0.85646000	-5.19523200
F	-2.22502000	0.60833700	-5.92123700
F	-3.56658400	-1.09115700	-5.85480900
O	-1.83157500	0.47400300	-3.33902300
O	0.09433400	0.02276400	-0.05239100
C	0.04961300	0.77954000	0.96370100
C	0.83903000	0.19326700	2.15006100
F	0.79896100	0.97062600	3.23571500
F	0.35780500	-1.00837000	2.47627800
F	2.13941500	0.03197500	1.82100000
C	-0.58495500	2.00104600	1.13115200
C	-1.35230100	2.59847800	0.10184000
C	-1.97427900	3.98546300	0.38465600
F	-1.84717200	4.78419400	-0.67809300
F	-3.27890700	3.84940600	0.65325700
F	-1.39942400	4.59881000	1.43190800
O	-1.62073000	2.12292400	-1.01279600

C	1.68662700	-1.83076100	-1.52998000
C	0.45550400	-2.47504700	-1.73214400
C	0.00541300	-3.45368600	-0.85322400
C	0.78091000	-3.78399900	0.26123600
C	2.01294400	-3.16460200	0.46766500
C	2.49273700	-2.20218500	-0.42668800
N	3.70325400	-1.53770200	-0.13800700
C	4.89435100	-1.64120300	-0.83551800
O	4.77522000	-2.51895200	-1.83498600
C	5.83868700	-2.71137600	-2.82118400
C	7.08017400	-3.28731600	-2.13860400
C	5.21672900	-3.72568300	-3.77636400
C	6.13309000	-1.39395300	-3.53999200
O	5.88775800	-1.02743400	-0.51607200
H	2.89180400	0.99752500	-0.73030200
H	3.54580700	2.37725800	1.18565700
H	2.64793100	4.68881300	1.44865800
H	1.07724700	5.60904200	-0.26215900
H	0.44638200	4.22034500	-2.23346400
H	-4.23394700	-1.81299200	-3.61433900
H	-0.45902000	2.53030900	2.07025000
H	-0.15563600	-2.18218100	-2.58644900
H	-0.95911600	-3.93377100	-1.01603200
H	0.42516000	-4.53110100	0.97419500
H	2.62411400	-3.41947900	1.33550500
H	3.79746400	-1.11605400	0.77906100
H	6.82073900	-4.20432400	-1.58719500
H	7.83303400	-3.54556600	-2.89933700
H	7.51380600	-2.56257600	-1.43884300
H	4.27932400	-3.32907200	-4.18730200
H	5.90661100	-3.94583900	-4.60492000
H	4.98758000	-4.66419700	-3.24982400
H	6.55419900	-0.65510200	-2.84667600
H	6.85802500	-1.57326600	-4.34895800
H	5.20993200	-0.98086200	-3.96904800
N	-0.08375800	-1.44947800	-4.75453600
C	0.54886300	-0.24665100	-4.78037600
C	1.84927800	-0.09172400	-5.38893600
C	2.51274700	-1.19860000	-5.83326300
C	1.89528100	-2.48342000	-5.73743500
C	0.56024100	-2.54542300	-5.18971900
C	-0.12036700	-3.81478500	-5.12371300
C	-1.53053500	-3.87780300	-4.61544300
C	0.54953500	-4.94381600	-5.55946000
C	1.86306900	-4.87879300	-6.08176400
C	2.52221000	-3.66637700	-6.18021000
H	-0.10564500	0.62701200	-4.76055400
H	2.27594800	0.90859600	-5.45924700
H	3.50557200	-1.11881100	-6.28269200
H	-2.17710800	-3.18391700	-5.17162600
H	-1.93544200	-4.89549300	-4.70272700
H	-1.59795100	-3.57187000	-3.56077700
H	0.04542300	-5.91211400	-5.50987500
H	2.35151400	-5.79559100	-6.41940800
H	3.52977900	-3.61123400	-6.59776100

G1

O	3.58477900	-0.59411800	-2.67421700
C	2.85873700	-0.37241000	-1.71306100
C	1.80778300	0.66964700	-1.88271200
C	1.56749100	1.77697600	-0.94793100
C	2.60126900	2.11700400	0.07487200
C	3.97418700	1.97855100	-0.18445800
C	4.91000500	2.33826000	0.78614100
C	4.48217900	2.82622800	2.02401900
C	3.11497900	2.97564000	2.28249000
C	2.17828800	2.63662300	1.30797300
O	0.51379100	2.41022900	-1.01918300
Cu	-1.39439000	-0.90155600	-2.85930700
O	-3.22020600	-1.45505700	-2.27835300
C	-4.22260500	-1.35815200	-3.02797500
C	-5.56018700	-1.36445600	-2.26322800
F	-5.58367300	-0.34830600	-1.39280300
F	-6.61718200	-1.24118200	-3.07546800
F	-5.69370200	-2.50519500	-1.57784900
C	-4.23990800	-1.25059500	-4.42638600
C	-3.05198700	-1.28569500	-5.16265700
C	-3.12277900	-1.32731000	-6.70015800
F	-2.42101700	-0.31813200	-7.22240100
F	-2.59416700	-2.47658600	-7.14293300
F	-4.37763200	-1.24497800	-7.15945300
O	-1.87019900	-1.31631900	-4.72896200
O	-1.00714200	-0.40584900	-0.97349100
C	-1.75215400	0.37452400	-0.30705000
C	-1.56299800	0.22717400	1.21436700
F	-2.52393600	0.82445700	1.92534100
F	-1.52934300	-1.06580500	1.56507600
F	-0.38536800	0.77005200	1.58627500

C	-2.66071900	1.32337500	-0.75890500
C	-2.82175100	1.63112900	-2.13034500
C	-3.94681900	2.61266100	-2.52294900
F	-3.48916300	3.55621000	-3.34874100
F	-4.91016200	1.92829900	-3.16806600
F	-4.51329100	3.21907900	-1.47060500
O	-2.18925200	1.16439500	-3.09369800
C	2.98115400	-1.03084400	-0.38842400
C	1.81807600	-1.20317600	0.37857200
C	1.88300600	-1.68096200	1.68569800
C	3.12820900	-1.96653100	2.25017300
C	4.29136000	-1.82455800	1.49118000
C	4.23511300	-1.38761400	0.16338800
N	5.43271500	-1.23833800	-0.56740700
C	5.82271900	-2.01410800	-1.64725900
O	4.91105600	-2.95869100	-1.89543800
C	4.99350400	-3.82339700	-3.07097300
C	6.21430300	-4.73656800	-2.94874700
C	3.69660300	-4.62393400	-2.98041100
C	5.02645900	-2.98502700	-4.34931400
O	6.87679700	-1.84967200	-2.21971300
H	4.32276000	1.60523400	-1.14874700
H	5.97718200	2.24165600	0.57222800
H	5.21551200	3.09778100	2.78717900
H	2.77992800	3.36042800	3.24837100
H	1.10935200	2.74834600	1.48920500
H	-5.19042000	-1.18548800	-4.94653900
H	-3.26313900	1.85584900	-0.02943600
H	0.85124400	-0.95585400	-0.06086700
H	0.96418100	-1.80750500	2.25983200
H	3.19703200	-2.31773900	3.28251200
H	5.26549500	-2.07185100	1.91876700
H	6.19705800	-0.73003400	-0.13737900
H	6.17934200	-5.29592900	-2.00105300
H	6.21954200	-5.46225700	-3.77675100
H	7.14161800	-4.15125400	-2.98224600
H	2.83441800	-3.94489000	-3.01292100
H	3.62099500	-5.32695400	-3.82323800
H	3.65892400	-5.19224400	-2.03925100
H	5.95306400	-2.40094300	-4.41057000
H	4.96588500	-3.65104900	-5.22360900
H	4.17517400	-2.29343300	-4.37351900
N	0.52548900	-0.69917100	-3.42044800
C	0.98867200	0.65021100	-3.16624300
C	1.75991300	1.29318500	-4.27530000
C	2.13292100	0.59217200	-5.36469000
C	1.83625100	-0.81309900	-5.46476300
C	1.02571200	-1.42064800	-4.42954500
C	0.74483400	-2.83604800	-4.48997300
C	-0.00100700	-3.51557700	-3.37723700
C	1.23267700	-3.55764800	-5.56560000
C	1.98646200	-2.95006800	-6.59073100
C	2.29106500	-1.59488200	-6.53212400
H	0.11125700	1.28201000	-2.93575400
H	2.00440600	2.35051000	-4.15498900
H	2.68348500	1.06830500	-6.17926400
H	-1.08487800	-3.32246500	-3.43894800
H	0.13549000	-4.60440700	-3.42709900
H	0.34369300	-3.16644200	-2.39209900
H	1.04250800	-4.63235300	-5.61001900
H	2.34772200	-3.55576000	-7.42439100
H	2.89239400	-1.12691800	-7.31440700

TS_{D1A}

O	3.45438700	-0.35851100	-2.77985200
C	2.32445100	-0.46079300	-2.34396800
C	1.25861100	0.51607200	-2.80881300
C	1.04676000	1.81706400	-2.13557900
C	1.90873600	2.29492500	-1.01023100
C	2.28994200	1.49668500	0.07692800
C	3.00399700	2.04747900	1.14206300
C	3.36309900	3.39665700	1.12385600
C	2.97900600	4.20291100	0.04630000
C	2.24251800	3.66077800	-1.00450800
O	0.17644700	2.57588100	-2.56909400
Cu	-2.40593000	0.16507100	-1.26309100
O	-2.85225200	-1.71736000	-1.00501000
C	-3.24962500	-2.50835600	-1.90550900
C	-3.48519100	-3.94390300	-1.39916200
F	-4.29266300	-3.94159600	-0.33900300
F	-4.02360300	-4.73155900	-2.33798600
F	-2.31189000	-4.48548700	-1.03313700
C	-3.43381600	-2.24149400	-3.26140500
C	-3.14529000	-0.97677400	-3.79255100
C	-3.27665000	-0.77349900	-5.31561000
F	-4.46074200	-0.21434600	-5.59262600
F	-2.31595700	0.02381500	-5.77724800
F	-3.20747400	-1.94058100	-5.97628200

O	-2.83098100	0.06280100	-3.16158400
O	-1.55115400	0.22515200	0.47059500
C	-1.04286200	1.26113500	0.99029000
C	-0.37418400	0.98156800	2.34938300
F	0.23980800	2.06104000	2.84830000
F	-1.29748400	0.58376500	3.23052600
F	0.53658900	0.00462800	2.23570600
C	-1.05536700	2.56207600	0.49473300
C	-1.75815500	2.87880800	-0.68122300
C	-1.83104500	4.36240100	-1.09522600
F	-2.02422000	4.50663200	-2.39564100
F	-2.86509300	4.93261700	-0.44758700
F	-0.72066200	5.02484200	-0.74225500
O	-2.42185200	2.09582200	-1.40402100
C	1.94027600	-1.42626200	-1.27063200
C	0.59134100	-1.78081100	-1.08518000
C	0.17133500	-2.51287600	0.02222700
C	1.10450100	-2.89154900	0.98764200
C	2.45023500	-2.56659100	0.81909600
C	2.89076700	-1.85676700	-0.30363500
N	4.25410300	-1.50167700	-0.37433000
C	5.17010700	-1.94270100	-1.30766700
O	4.62119900	-2.89137600	-2.07510300
C	5.32579700	-3.45541000	-3.22211100
C	6.57465200	-4.20384800	-2.75254100
C	4.29265100	-4.42244500	-3.79381900
C	5.66033600	-2.34967900	-4.22428700
O	6.31247400	-1.54429400	-1.34623100
H	1.97911000	0.45832600	0.12984600
H	3.25872000	1.42057400	1.99937700
H	3.92940000	3.82491600	1.95418600
H	3.24715600	5.26178000	0.03204600
H	1.90631900	4.28715900	-1.83143700
H	-3.74534500	-3.04216300	-3.92444400
H	-0.51939700	3.34438300	1.02403000
H	-0.13606900	-1.48353100	-1.83888500
H	-0.88297800	-2.75679800	0.13696800
H	0.78536700	-3.44300800	1.87477500
H	3.19283100	-2.86994600	1.55993400
H	4.65447400	-0.94278700	0.37020700
H	6.30556500	-4.95420900	-1.99293300
H	7.03325500	-4.72802700	-3.60545300
H	7.30750000	-3.51047600	-2.32244700
H	3.36855200	-3.88566000	-4.04264700

H	4.67671200	-4.90167500	-4.70657300
H	4.05152300	-5.20501200	-3.05877100
H	6.39246500	-1.64999300	-3.80232900
H	6.07918100	-2.79715400	-5.13906100
H	4.75309300	-1.78891200	-4.48385600
N	-0.03389100	-1.21601900	-4.09350700
C	0.22049300	0.16117400	-3.86679100
C	1.30644800	0.85717500	-4.59805000
C	2.15388200	0.10539100	-5.42092800
C	1.91366800	-1.30298700	-5.59608500
C	0.78934900	-1.90972400	-4.90660800
C	0.52985600	-3.30398800	-5.09642800
C	-0.61355200	-3.95594700	-4.38088600
C	1.34465200	-4.03973700	-5.97250100
C	2.40858500	-3.44460700	-6.64362800
C	2.69707000	-2.08184300	-6.44384000
H	-0.69604300	0.74509200	-3.72920700
H	1.26913200	1.94366700	-4.66294000
H	2.97765700	0.57973300	-5.95592300
H	-1.57009800	-3.59388800	-4.78579500
H	-0.58094600	-5.04850100	-4.49296700
H	-0.61209900	-3.70361800	-3.31075300
H	1.13974100	-5.10319900	-6.11456100
H	3.03386400	-4.03871400	-7.31328100
H	3.54466200	-1.62156300	-6.95648700

TD-DFT Computational data

TD-DFT calculations were carried out using the Gaussian 09 software at The Hong Kong University of Science and Technology. All computations were performed at the B3LYP level of theory[9] using 6-31G(d) base set for all atoms, with the resulting structures confirmed to be stationary points through vibrational frequency analysis.

Supplementary Tables

Supplementary Table 6. TD-DFT calculation results for **3a** using the optimized S0 structure.

Compd.	Excited State	Transition configuration	Transition Energy	Oscillator Strength
**3a**	S_1	HOMO->LUMO (94%)	405 nm, 3.06 eV	0.0037
	S_2	HOMO->L+1 (98%)	381 nm, 3.25 eV	0.0067
	S_3	H-5->LUMO (12%), H-1->LUMO (65%)	378 nm, 3.28 eV	0.0057

Supplementary Table 7. TD-DFT calculation results for **3e** using the optimized S0 structure.

Compd.	Excited State	Transition configuration	Transition Energy	Oscillator strength
**3e**	S_1	HOMO->LUMO (96%)	440 nm, 2.82 eV	0.0032
	S_2	H-1->LUMO (57%), HOMO->L+1 (30%)	362 nm, 3.43 eV	0.0049
	S_3	H-1->LUMO (25%), HOMO->L+1 (68%)	361 nm, 3.43 eV	0.0139

Supplementary Table 8. TD-DFT calculation results for **3g** using the optimized S0 structure.

Compd.	Excited State	Transition configuration	Transition Energy	Oscillator Strength
**3g**	S_1	HOMO->LUMO (94%)	419 nm, 2.96 eV	0.005
	S_2	HOMO->L+1 (97%)	361 nm, 3.43 eV	0.0072
	S_3	H-1->LUMO (75%)	349 nm, 3.28 eV	0.0167

Supplementary Table 9. TD-DFT calculation results for **3j** using the optimized S0 structure.

**Compd.**	**Excited State**	**Transition configuration**	**Transition Energy**	**Oscillator strength**
**3j**	S_1	HOMO->LUMO (93%)	399 nm, 3.11 eV	0.0038
	S_2	HOMO->L+1 (97%)	361 nm, 3.43 eV	0.0076
	S_3	H-3->L+1 (12%), H-1->LUMO (50%)	336 nm, 3.69 eV	0.005

Supplementary Table 10. TD-DFT calculation results for **3k** using the optimized S0 structure.

Compd.	Excited State	Transition configuration	Transition Energy	Oscillator strength
**3k**	S_1	HOMO->LUMO (92%)	386 nm, 3.21 eV	0.0049
	S_2	HOMO->L+1 (97%)	360 nm, 3.44 eV	0.0081
	S_3	H-4->L+1 (46%),<br>H-1->L+1 (35%)	334 nm, 3.71 eV	0.001

Supplementary Table 11. TD-DFT calculation results for **3r** using the optimized S0 structure.

Compd.	Excited State	Transition configuration	Transition Energy	Oscillator Strength
**3r**	S_1	HOMO->LUMO (92%)	395 nm, 3.14 eV	0.0045
	S_2	HOMO->L+1 (97%)	385 nm, 3.22 eV	0.0062
	S_3	H-3->L+1 (27%),<br>H-1->LUMO (30%),<br>H-1->L+1 (20%)	383 nm, 3.43 eV	0.0026

Supplementary Table 12. TD-DFT calculation results for **3ag** using the optimized S0 structure.

Compd.	Excited State	Transition configuration	Transition Energy	Oscillator strength
**3ag**	S_1	HOMO->LUMO (94%)	401 nm, 3.09 eV	0.0038
	S_2	HOMO->L+1 (98%)	355 nm, 3.49 eV	0.0091
	S_3	H-5->LUMO (20%), H-2->LUMO (27%), H-1->LUMO (30%)	337 nm, 3.28 eV	0.0033

Supplementary Table 13. TD-DFT calculation results for **3aj** using the optimized S0 structure.

Compd.	Excited State	Transition configuration	Transition Energy	Oscillator strength
**3aj**	S_1	HOMO->LUMO(91%)	390 nm, 3.18 eV	0.0034
	S_2	HOMO->L+1 (91%)	345 nm, 3.59 eV	0.0113
	S_3	H-2->LUMO (15%), H-1->LUMO (40%)	332 nm, 3.73 eV	0.0034

Supplementary Table 14. TD-DFT calculation results for **3an** using the optimized S0 structure.

Compd.	Excited State	Transition configuration	Transition Energy	Oscillator strength
**3an**	S_1	HOMO->LUMO (94%)	407 nm, 3.05 eV	0.0033
	S_2	HOMO->L+1 (98%)	360 nm, 3.44 eV	0.0093
	S_3	H-5->LUMO (15%), H-1->LUMO (64%)	340 nm, 3.65 eV	0.0073

Supplementary Table 15. TD-DFT calculation results for **3ao** using the optimized S0 structure.

Compd.	Excited State	Transition configuration	Transition Energy	Oscillator strength
**3ao**	S_1	HOMO->LUMO (96%)	424 nm, 2.92 eV	0.0019
	S_2	HOMO->L+1 (99%)	376 nm, 3.30 eV	0.0068
	S_3	H-5->LUMO (16%), H-1->LUMO (65%)	346 nm, 3.58 eV	0.0079

Supplementary References

(1) J. Wang, Y. Li, J. Sun, H. Wang, Z. Jin, Y. R. Chi, *ACS Catal.* **2018**, *8*, 9859.

(2) N. Thirupathi, Y. K. Kumar, R. Kant, M. S. Reddy, *Adv. Synth. & Catal.* **2014**, *356*, 1823.

(3) J. Liu, D. Ba, Y. Chen, S. Wen, G. Cheng, *Chem. Commun.*, **2020**, *56*, 4078.

(4) Z.-S. Chen, F. Yang, H. Ling, M. Li, J.-M. Gao, K. Ji, *Org. Lett.* **2016**, *18*, 5828.

(5) (a) C. T. Lee, W. T. Yang, R. G. Parr, *Phys. Rev. B* **1988**, *37*, 785; (b) A. D. Becke, *J. Chem. Phys.* **1993**, *98*, 5648.

(6) F. Weigend, R. Ahlrichs, *Phys. Chem. Chem. Phys.* **2005**, *7*, 3297.

(7) (a) S. Grimme, J. Antony, S. Ehrlich, H. Krieg, *J. Chem. Phys.* **2010**, *132*, 154104; (b) S. Grimme, S. Ehrlich, L. Goerigk, *J. Compt. Chem.* **2011**, *32*, 1456.

(8) A. V. Marenich, C. J. Cramer, D. G. Truhlar, *J. Phys. Chem. B* **2009**, *113*, 6378.

(9) M. J. Frisch, G. W. Trucks, H. B. Schlegel, G. E. Scuseria, M. A. Robb, J. R. Cheeseman, G. Scalmani, V. Barone, B. Mennucci, G. A. H. Petersson, M. Nakatsuji, X. Caricato, H. P. F. Li, A. Hratchian, J. Izmaylov, G. Bloino, J. L. Zheng, M. Sonnenberg, M. Hada, K. Ehara, R. Toyota, J. Fukuda, M. Hasegawa, T. Ishida, Y. Nakajima, O. Honda, H. Kitao, T. Nakai, J. A. Vreven, J. E. Montgomery Jr., F. Peralta, M. Ogliaro, J. J. Bearpark, E. Heyd, K. N. Brothers, V. N. Kudin, T. Staroverov, R. Keith, J. Kobayashi, K. Normand, A. Raghavachari, J. C. Rendell, S. S. Burant, J. Iyengar, M. Tomasi, N. Cossi, J. M. Rega, M. Millam, J. E. Klene, J. B. Knox, V. Cross, C. Bakken, J. Adamo, R. Jaramillo, R. E. Gomperts, O. Stratmann, A. J. Yazyev, R. Austin, C. Cammi, J. W. Pomelli, R. L. Ochterski, K. Martin, V. G. Morokuma, G. A. Zakrzewski, P. Voth, J. J. Salvador, S. Dannenberg, A. D. Dapprich, O. Daniels, J. B. Farkas, J. V. Foresman, J. Ortiz, J. Cioslowski, D. J. Fox, Gaussian 09 Revision C.01, 2010.
