# Supplementary material for: Copper-catalyzed alkyne oxidation/Büchner-type ring-expansion to access benzo[6,7]azepino[2,3-b]quinolines and pyridine-based diones
Source: Commun Chem. 2023 Feb 20;6:35. doi: 10.1038/s42004-023-00840-6 (PMC9941089; doi:10.1038/s42004-023-00840-6)
Supplement: Supplementary file 2 — Description of Additional Supplementary Files [file 42004_2023_840_MOESM2_ESM.pdf]

Description of Additional Supplementary Files

File name: Supplementary Data 1

Description: Cartesian coordinates for the optimized structures.

File name: Supplementary Data 2

Description: NMR spectra.

File name: Supplementary Data 3

Description: Cif file of 3a

File name: Supplementary Data 4

Description: Cif file of 3r

File name: Supplementary Data 5

Description: Cif file of 3aba

File name: Supplementary Data 6

Description: Cif file of 5p

File name: Supplementary Data 7

Description: Cif file of 8
